# First-in-class small molecule potentiators of cancer virotherapy

**DOI:** 10.1038/srep26786

**Published:** 2016-05-26

**Authors:** Mark H. Dornan, Ramya Krishnan, Andrew M. Macklin, Mohammed Selman, Nader El Sayes, Hwan Hee Son, Colin Davis, Andrew Chen, Kerkeslin Keillor, Penny J. Le, Christina Moi, Paula Ou, Christophe Pardin, Carlos R. Canez, Fabrice Le Boeuf, John C. Bell, Jeffrey C. Smith, Jean-Simon Diallo, Christopher N. Boddy

**Affiliations:** 1 Departments of Chemistry and Biomolecular Sciences, University of Ottawa, Ottawa, Ontario, Canada; 2 Centre for Innovative Cancer Research, Ottawa Hospital Research Institute, Ottawa, Ontario, Canada; 3 Department of Biochemistry, Microbiology and Immunology, University of Ottawa, Ontario, Canada; 4 Department of Chemistry and Institute of Biochemistry, Carleton University, Ottawa, Ontario, Canada

## Abstract

The use of engineered viral strains such as gene therapy vectors and oncolytic viruses (OV) to selectively destroy cancer cells is poised to make a major impact in the clinic and revolutionize cancer therapy. In particular, several studies have shown that OV therapy is safe and well tolerated in humans and can infect a broad range of cancers. Yet in clinical studies OV therapy has highly variable response rates. The heterogeneous nature of tumors is widely accepted to be a major obstacle for OV therapeutics and highlights a need for strategies to improve viral replication efficacy. Here, we describe the development of a new class of small molecules for selectively enhancing OV replication in cancer tissue. Medicinal chemistry studies led to the identification of compounds that enhance multiple OVs and gene therapy vectors. Lead compounds increase OV growth up to 2000-fold *in vitro* and demonstrate remarkable selectivity for cancer cells over normal tissue *ex vivo* and *in vivo*. These small molecules also demonstrate enhanced stability with reduced electrophilicity and are highly tolerated in animals. This pharmacoviral approach expands the scope of OVs to include resistant tumors, further potentiating this transformative therapy. It is easily foreseeable that this approach can be applied to therapeutically enhance other attenuated viral vectors.

Genetically attenuated viruses form the basis of a growing number of biotechnology and pharmaceutical platforms, including oncolytic viruses (OVs) and gene therapy vectors for the treatment of cancers. In particular, OV therapy has shown significant promise with strong clinical evidence demonstrating that OVs can lead to profound anti-tumor responses in patients^1^,^2^ with very mild side-effects, often described as acute flu-like symptoms^2^. OVs are based on a wide range of viral backbones from small RNA viruses like rhabdoviruses, to large DNA viruses such as herpesviruses (HSV) and are currently being evaluated in clinical trials to treat a range of cancer types^3^. OV therapeutics have been explored for over 60 years and in 2005, the oncolytic adenovirus H101 was approved in China for the treatment of head and neck cancers. Oncolytic HSV-1 expressing granulocyte macrophage colony stimulating factor has been approved in North America based on favourable phase III clinical trial data in melanoma (NCT00769704).

OVs have been selectively engineered to take advantage of several hallmarks of cancer in order to preferentially replicate in tumor cells. The increased metabolism of cancers, their resistance to apoptosis, and their often defective innate antiviral response provide fertile ground for most viruses^4^. Genetic attenuation of specific virulence genes generates viruses that are selectively attenuated in normal host cells but still capable of infecting cancer cells. When effective, OVs lead to durable cures by direct lysis of cancer cells, vascular shutdown, and through generation of a strong anti-tumor immune response^4^.

Heterogeneity in the clinical response to OVs remains a major hurdle to overcome, as demonstrated in several human clinical trials^2^,^3^,^5^. Because an estimated 30–35% of tumors have effective antiviral defenses^4^,^6^,^7^, it is no surprise that OVs show tremendous effects in some models and patients but have minimal impact in others. Given that innate cellular antiviral responses can compromise the therapeutic efficacy of OVs, developing pharmaceuticals that target this complex cellular defense mechanism will have important clinical ramifications, improving current OV therapies and enabling the development of new therapeutic strategies^3^,^8^,^9^.

We recently identified compounds from a high-throughput screen that sensitize resistant cancer cells to infection with the rhabdovirus-based OV named VSV∆51^10^. VSV∆51 is an engineered mutant of vesicular stomatitis virus (VSV) that is highly sensitive to interferon (IFN) and its antiviral effects. Much like HSV-1 and many other OVs, VSVΔ51 faces a roadblock in tumors that retain effective cellular antiviral responses. The most active compound from this screen (**1**, Figs 1a and 2), was shown to enhance VSVΔ51 oncolysis *in vitro* and *in vivo*, where virus output was increased by as much as 1,000-fold in cancer cells^10^. **1** has been shown to dampen the activation of antiviral responses in cancer cells, including the transcriptional response to type I IFN^10^. Given the impact of tumor heterogeneity on the clinical response to OVs, there is an unmet need and an opportunity to develop small molecule potentiators such as **1** to improve OV therapy. The electrophilic nature of **1** prompted us to investigate the scaffold to identify active analogs with more favourable physiochemical properties and explore structure-activity relationships. Herein we report the development of first-in-class small molecules with favourable pharmacological properties and demonstrate that they significantly enhance OV propagation selectively in resistant cancers *in vitro*, *ex vivo*, and *in vivo*, completing proof-of-concept studies for a pharmacoviral combination approach to enhancing OV therapy.

## Results

### 1 suffers from rapid degradation

**Figure 1:**
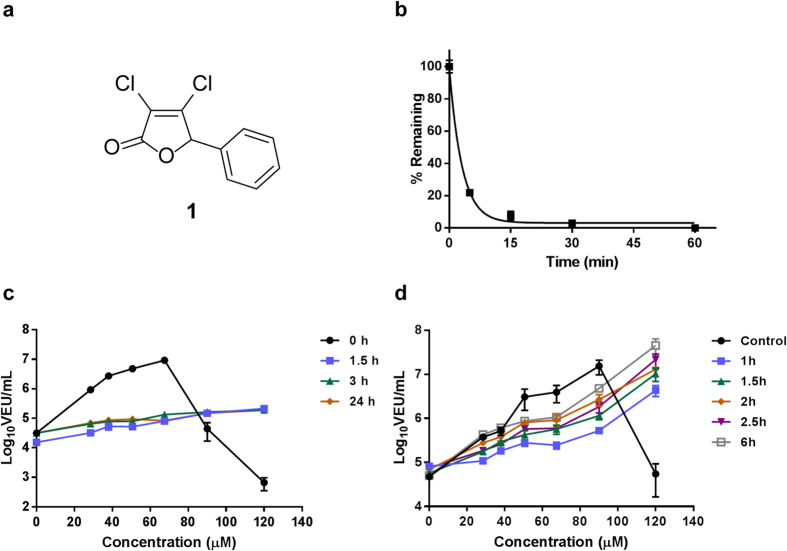
Compound 1 acts quickly but suffers from rapid degradation. (**a**) Structure of **1** (3,4-dichloro-5-phenyl-5H-furan-2-one). (**b**) Stability of **1** in mouse serum over time measured by HPLC (**c**) **1** was incubated in sterile water for 0 h, 1.5 h, 3 h or 24 h before being used to treat 786-0 cells at different concentrations. 4 hours post-treatment, cells were infected with VSVΔ51 expressing firefly luciferase (VSVΔ51-FLuc) at a multiplicity of infection (MOI) of 0.005. 40 hours later, virus output in viral expression units (VEUs) per millilitre was measured with a previously describe luciferase reporter assay^12^. (**d**) 786-0 cells were treated with **1** at various doses. **1** was removed and replaced with fresh media after 1 h, 1.5 h, 2 h, 2.5 h and 6 h. **1** was not removed in the control condition. 4 hours post-treatment, cells were infected with VSVΔ51 expressing firefly luciferase VSVΔ51-FLuc at an MOI of 0.005. For the condition where **1** was replaced with fresh media 6 h after treatment, infection was performed immediately following media replacement. 40 hours later, virus output in viral expression units (VEUs) per millilitre was measured with a previously describe luciferase reporter assay^12^.

To gain a better understanding of the physiochemical nature of **1**, we measured the compound’s stability in mouse serum with an LC/MRM assay (Fig. 1b). Degradation occurred very quickly (t_1/2_ = 2.1 min). **1** showed similar degradation behaviour in a simple aqueous medium (data not shown), which resulted in reduced biological activity. Pre-incubation of **1** in aqueous media for as little as 90 min was sufficient to eliminate the robust enhancement of VSVΔ51 titers otherwise observed (Fig. 1c). However, the effect of **1** on viral growth is rapid and sustained. Treatment of cells with **1** for as little as 60 min followed by its complete removal from cell culture media and subsequent infection with VSVΔ51 of drug treatment resulted in substantial enhancement of viral titers (Fig. 1d).

### Derivatives with improved properties and activity

These observations provided a rationale to derive novel compounds based on **1** with increased stability. Taking advantage of the versatile reactions that mucochloric acid can undergo^11^, a diverse set of analogs (**1**–**10**) was synthesized to reveal structure-activity relationships (Supplementary Table 1). Analogs were screened for their ability to augment VSVΔ51 activity in OV resistant 786-0 renal cancer cells using a previously described high-throughput luciferase reporter-based titration assay^12^ and their maximal viral enhancing activity was compared to that of **1**. Cytotoxicity in the presence and absence of virus was also assessed using an alamarBlue^®^ metabolic dye. Analog stability was measured with a plasma stability assay as well as a glutathione stability assay, which indicated physiochemical susceptibility to act as a Michael-acceptor.

Substitution of the β-chlorine with an alkyl amine resulted in compounds with dramatically increased stability but loss of viral enhancement activity (**3** and **4**). Removal of the aryl group (**5**) or replacement with a methoxide group (**6**) resulted in active compounds with poor stability similar to **1** (Supplementary Table 1). Encouragingly, compounds with a pyrrole-based scaffold (**9** and **10**) enhanced viral growth and showed remarkably improved stability by both the GSH and plasma stability assays. The LC/MS trace observed in the GSH stability assay also showed that **10**, contrary to **1**, cleanly reacted with the nucleophile to form the glutathione adduct as the sole detectable product (Fig. 3a,b). Similar to **1**, the impact of **10** on viral growth was found to be rapid and sustained (Fig. 3c).

Given these results, we decided to further explore the pyrrole scaffold from **10** (Supplementary Table 1). Analogs with alterations to the hydroxyl group (**11–15**) abolished activity, as did alterations to the dichloro- moiety (**16**–**21**). We then decided to investigate various substitutions on the pyrrole amine (Supplementary Table 1). Cyclopropyl and morpholine containing analogs (**25** and **28**) displayed retained activity and remarkably improved *in vitro* toxicity and stability profiles. Examination of arylamine containing analogs showed that the spacer length between the amine and phenyl ring was optimal at three carbons (**10**, **29**, **30** and **31**). Testing a large set of substituted benzylamine derivatives (**36–51**) demonstrated that the 4-trifluoromethyl substituted system (**40**) possessed improved activity (2,000 fold enhancement, 105% of **1**) and improved stability. Overall the activity and stability of the analogs could be readily tuned.

While initial analog screening studies were carried out in highly virus-resistant human 786-0 renal cancer cells, mouse cancer cell lines originating from skin (B16-F10), colon (CT26) and breast (4T1), were also sensitized to VSVΔ51 by pyrrole-based analogs (Supplementary Figs 1–3). **1**, **10** and several other pyrrole analogs increased the oncolytic activity of Maraba MG-1 virus^13^ (Supplementary Fig. 3) as well as spread of oncolytic HSV-1 expressing GFP^14^ as observed by fluorescence microscopy and standard plaque assay (Fig. 3d,e, and Supplementary Fig. 4), suggesting the compounds have a broader scope of application for virus-based therapies. Luciferase transgene expression delivered to human A549 lung cancer cells by non-replicating adenovirus and adeno-associated virus (AAV) vectors (Supplementary Fig. 5a,b respectively) was also enhanced by the compounds, suggesting the potentiating effect of the compounds is not limited to replicating viruses.

### Selective viral enhancement in *ex vivo* tumor specimens

**Figure 2:**
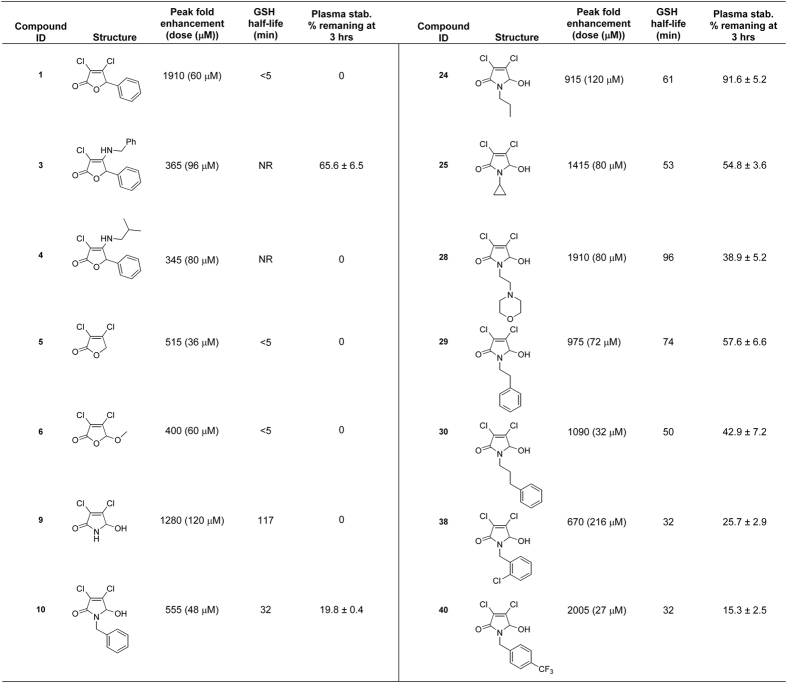
The structure of the analogs investigated in this study, their ability to enhance VSVΔ51 activity, their half-life in a glutathione challenge assay, and their stability in serum.

To facilitate evaluation of a larger number of compounds prior to *in vivo* testing, we chose to test a subset of analogs (**1**, **10**, **28**–**30** and **40**, Fig. 2) for their ability to enhance VSVΔ51 oncolysis in *ex vivo* tissue samples using an established method^15^. Tissue samples from VSVΔ51 resistant CT26 murine colon cancer tumors^10^,^16^,^17^ as well as normal mouse brain, lung and spleens were cored. Viable cores were selected for subsequent treatment with each compound and VSVΔ51 expressing green fluorescent protein (VSVΔ51-GFP). Figure 4a shows representative images of infected cores that were pre-treated with an optimized dose of compound. Corresponding viral titers as determined by plaque assay are shown in Fig. 4b–g and Supplementary Fig. 6. **1** and analogs robustly enhanced VSVΔ51-GFP titers in CT26 colon cancer specimens. There was little to no enhancement of VSVΔ51 in normal tissue specimens, indicating that the specificity of VSVΔ51 towards tumour tissue is maintained following treatment with **1** and its derivatives.

### Analogs are well-tolerated and enhance tumor specific OV replication *in vivo*

We proceeded to evaluate the *in vivo* tolerability of a subset of analogs, selected based on desirable physiochemical characteristics, *in vitro* activity and *ex vivo* activity. Compounds were administered intraperitoneally to non-tumor bearing Balb/c mice and body weight was monitored over several days. Mice were sacrificed when they reached the endpoint of 20% loss of body weight or showed significant outward signs of toxicity. Figure 5 shows that **1** leads to toxicity starting at a dose of 10 mg/kg. In contrast **10** was well tolerated up to a dose of 50 mg/kg and **24** and **28** up to 100 mg/kg.

Because it was highly active *ex vivo* and very well tolerated in mice, we proceeded to evaluate **28** for its ability to increase the infection of tumors with VSVΔ51 expressing luciferase (VSVΔ51-FLuc) *in vivo*. Balb/c mice were subcutaneously engrafted with CT26 cells and treated intra-tumorally with VSVΔ51-FLuc alone or in combination with **28**. We used an *in vivo* imaging system (IVIS) to measure luciferase activity associated to virus replication 24 h post treatment. Figure 6a–c shows that compared to VSVΔ51-FLuc alone, **28** significantly enhanced virus replication-associated luciferase expression specifically in the tumor. A similar treatment schedule was used to evaluate therapeutic efficacy in the human HT29 colon cancer xenograft model. The combination of **28** and VSVΔ51-FLuc significantly delayed tumour progression and improved survival compared to the mono-therapies (Fig. 6d,e). This demonstrates the feasibility and potential of using small molecules, such as **28**, in combination with OV therapy *in vivo*.

## Discussion

In this study, we identified a new class of pyrrole-based potentiators of tumor specific OV infection. Compared to the parent molecule **1**, these have substantially improved stability, reduced electrophilicity, and retained or improved ability to enhance growth of OVs in resistant cancer cell lines *in vitro* and *in vivo*.

**Figure 3:**
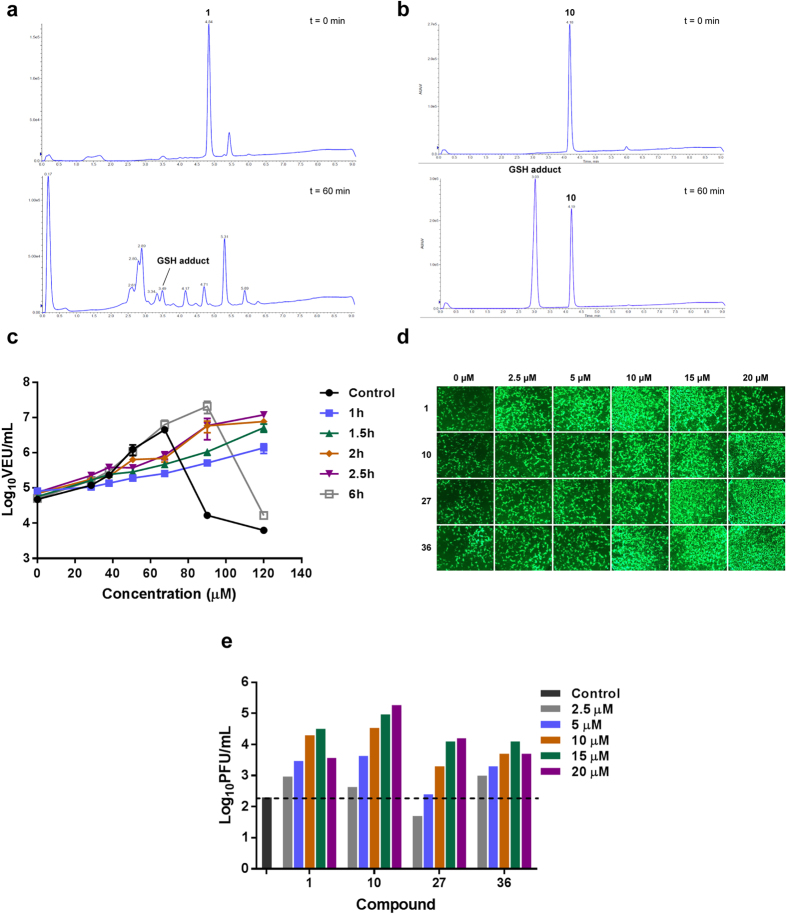
Pyrrole-based derivatives of 1 have improved stability but retain rapid, robust activity with oncolytic VSV and HSV-1 . (**a**) 250 μL of 40 mM DMSO stock solution of **1** was added to L-glutathione (15.4 mg, 5 mol equiv.) suspended in 250 μL DMSO. The resulting mixture was placed in a 37 °C shaker. 10 μL aliquots were removed and quenched in 990 μL water (containing 0.5% formic acid) at various time points (t = 0 minutes and t = 60 minutes shown). Analysis by ESI-LC-MS allowed for the identification and quantification of **1** and the glutathione adduct by UV-Vis at 254 nm. (**b**) 250 μL of 40 mM DMSO stock solution of **10** was added to L-glutathione (15.4 mg, 5 mol equiv.) suspended in 250 μL DMSO. The resulting mixture was placed in a 37 °C shaker. 10 μL aliquots were removed and quenched in 990 μL water (containing 0.5% formic acid) at various time points (t = 0 minutes and t = 60 minutes shown). Analysis by ESI-LC-MS allowed for the identification and quantification of **10** and the glutathione adduct by UV-Vis at 254 nm. (**c**) 786-0 cells were treated with **10** at various doses. **10** was removed and replaced with fresh media after 1 h, 1.5 h, 2 h, 2.5 h and 6 h. **10** was not removed in the control condition. 4 hours post-treatment, cells were infected with VSVΔ51 expressing firefly luciferase (VSVΔ51-FLuc at a multiplicity of infection (MOI) of 0.005). For the condition where **10** was replaced with fresh media 6 h after treatment, infection was performed immediately following media replacement. 40 hours later, virus output in viral expression units (VEUs) per millilitre was measured with a previously describe luciferase reporter assay^12^. (**d**) Mouse mammary carcinoma (4T1) cells were left untreated or, treated with **1**, **10**, **27**, or **36** for 4 h at various concentrations: 2.5 μM, 5 μM, 10 μM, 15 μM or 20 μM. ICP0-null HSV-N212eGFP was then added at MOI 0.005. eGFP fluorescence was detected 48 h after HSV infection. (**e**) HSV titers were determined 48 h after infection.

**Figure 4:**
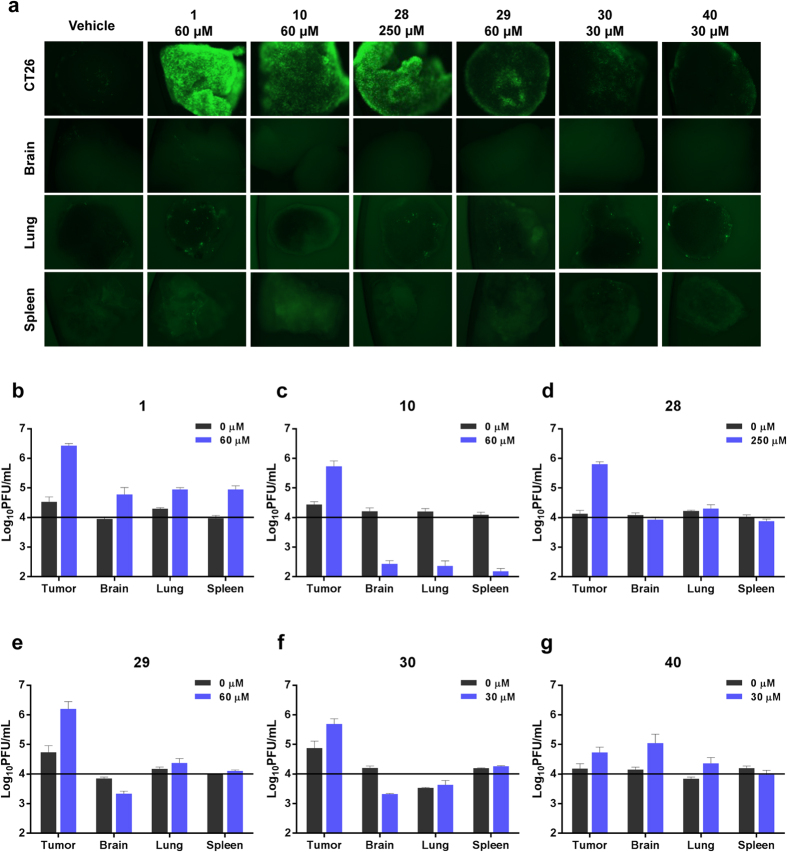
1 and its analogs selectively enhance the replication of oncolytic VSV in *ex vivo* tumor tissues. (**a**) CT26 (murine colon carcinoma) tumors were grown subcutaneously in Balb/c mice for 24 days and subsequently excised and cored, along with normal brain, lung and spleen tissues. Tissue samples were treated in triplicate with various concentrations of compounds for 4 hours prior to infection with 1 × 10^4^ plaque-forming units of vesicular stomatitis virus expressing GFP (VSVΔ51-GFP). Virus replication was assessed by fluorescence microscopy 24 hours post- infection. Representative images from each triplicate set for the most effective concentration are shown. (**b–g**) Infected cores and corresponding supernatants were collected 36 hours post-infection. VSVΔ51-GFP infection was quantified by standard plaque assay. Cores were homogenized prior to titering. Graphs show the sum of infectious titer from core and supernatant for each compound in each tissue type. Doses shown here are those that are depicted in panel (**a**). The horizontal black line on each graph at 1 × 10^4 ^PFU/mL represents the amount of VSVΔ51-GFP used to initially infect each core.

**Figure 5:**
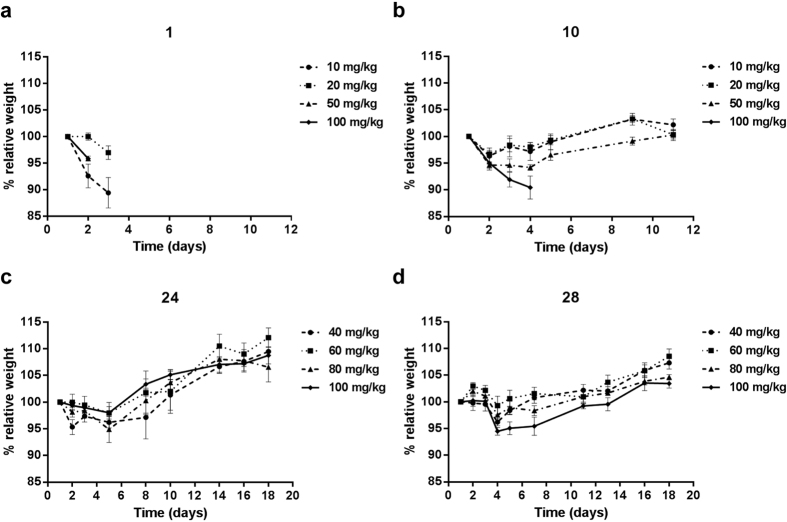
Pyrrole-based derivatives of 1 are substantially better tolerated in mice. Balb/c mice were given (**a**) **1**, (**b**) **10**, (**c**) **24**, or (**d**) **28** dissolved in DMSO via intraperitoneal administration. Five mice were assigned to each dose group for each compound. The dose was adjusted for individual mice based on weight. Graphs stop when the first mouse in the group was euthanized. (**a,b**) Mice were injected on Day 1 and weights were recorded over a 10 day period. (**c,d**) Mice were injected on Day 1, 3 and 5. Weights were recorded over an 18 day period. For all groups, weights are reported relative to initial weight on Day 1.

There is a growing list of OV clinical candidates for the treatment of cancer including VSV, vaccinia virus, reovirus, poliovirus, adenovirus, and herpes simplex virus-based platforms^3^. Talimogene laherparepvec (T-Vec, Amgen), an intra-tumorally delivered HSV-1-based oncolytic virus expressing granulocyte-macrophage colony-stimulating factor (GM-CSF), has been recently approved for treatment of melanoma in North America. Nevertheless, it is well-recognized in the field that combination therapies will be necessary to overcome the heterogeneous response observed with these potentially curative biologics. The first-in-class agents we have developed in this study robustly increase growth of oncolytic VSV, (Fig. 2 and Supplementary Table 1), Maraba MG-1 (Supplementary Fig. 3), and HSV-1 (Fig. 3e) in otherwise resistant cancer cells and therefore cater to an unmet need in this therapeutic area. In this regard, we consider that pyrrole-based molecules have significant clinical potential due to their *in vivo* activity and high tolerability. Indeed, the combination of **28** and oncolytic VSV delivered intra-tumorally robustly improved survival in the human HT-29 colon cancer model. Enhanced therapeutic effect of the combination treatment was also observed when **10** was delivered by intra-peritoneal route in the mouse CT26 colon cancer model (Supplementary Fig. 7), suggesting systemic administration is feasible.

Further to this, we have observed that the pyrrole-based compounds derived in this study can also enhance transgene expression levels from replication-defective gene therapy vectors such as AAV and adenovirus (Supplementary Fig. 5a,b). This extends the potential of using these small molecules for co-administration with cancer gene therapies. There are numerous registered clinical trials employing gene therapy vectors expressing transgenes for the treatment of various malignancies. It is easily foreseeable that the pharmaco-viral approach described here can be applied to therapeutically enhance other attenuated viral vectors as well.

**Figure 6:**
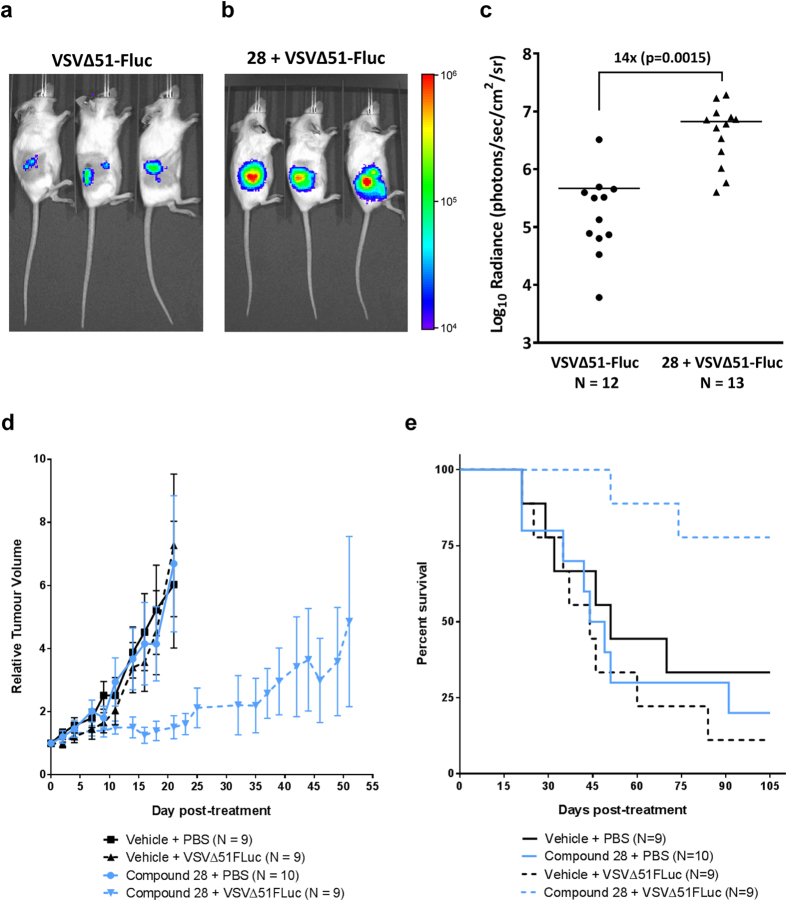
Pyrrole-based compound 28 enhances VSVΔ51 oncolytic activity in resistant syngeneic and xenograft tumor models. (**a,b**) VSVΔ51-resistant CT26 cells were subcutaneously engrafted into female Balb/c mice. After 11 days, mice were given 30 μL of vehicle (DMSO), or 40 mg/kg of **28** by intratumoral injection. Four hours later, mice were treated with 1 × 10^8^ plaque-forming units of VSVΔ51-FLuc. Virus replication was monitored twenty-four hours later by measuring luminescence using an IVIS (representative images are shown, color scale bar represents photons) and (**c**) tumor radiance was quantified. (**d**) HT29 cells were subcutaneously engrafted into female CD1 nude mice. When tumors reached 5 mm × 5 mm in size, mice were given 30 μL of vehicle (DMSO), or 40 mg/kg of **28** by intratumoral injection. Four hours later, mice were treated with 1 × 10^8^ plaque-forming units of VSVΔ51-FLuc. Tumor volumes were monitored every other day and average tumor volumes for each treatment group are shown. Tumor volume curves are terminated when the first mouse in each group is euthanized. Error bars correspond to standard error. (**e**) Survival was monitored over time. Log-rank tests indicated that treatment with **28** and virus significantly improved survival compared to vehicle control (p = 0.03), **28** alone (p = 0.006) or virus alone (p = 0.002). Surviving mice had static tumors that neither shrank nor grew for at least 2 weeks.

In this study, we have observed that similarly to **1**, pyrrole derivatives inhibit the production of IFNβ and various interferon-stimulated genes (ISGs) (Supplementary Fig. 8) and are able to block the antiviral effects of IFNβ (Supplementary Fig. 9). As such, it remains unclear whether this could also impact the adaptive immune response. This is relevant since in addition to viral oncolysis, generation of anti-tumor immunity is thought to contribute to the therapeutic effect of OVs^3^. Interestingly, other small molecules that dampen IFN response such as HDAC inhibitors can skew the immune response favourably in some contexts, where oncolytic virotherapy is coupled to a cancer vaccine approach^18^. Non-replicating gene therapy vectors such as adenovirus or AAV have been known to induce IFN^19^,^20^. Thus, dampening innate immunity is likely to be an advantage for cancer gene therapy applications where the objective is to express a therapeutic transgene, for example a pro-drug converting enzyme^21^,^22^. Clearly, the pyrrole derivatives described in this study lead to robust enhancement of luciferase transgene expressed by oncolytic VSV *in vivo* (Fig. 6) but also *in vitro* using non-replicating vectors (Supplementary Fig. 5a,b).

In addition to the broad potential therapeutic applications in combination with OVs and other virus-based therapies, the novel pyrrole-based molecules provide an arsenal of new probes to explore innate immunity. While the pyrrole-compounds described here clearly impact the antiviral IFN response as per **1** (Supplementary Figs 8,9), the precise molecular target remains elusive. The enhanced stability of the pyrrole-based analogs will provide opportunities to pursue target identification and/or activity based protein profiling. For example, the propargyl-based compound, **27**, synthesized in this study was found to be active and is amenable to click chemistry for inhibitor affinity capture or other relevant target identification strategies.

Relating to mechanism, one interesting property of the viral potentiators described in this study (including **1**) is the observed rapid and sustained activity. As little as 1 hour pre-treatment with the compounds, prior to their complete removal from cell culture media, was sufficient to observe enhanced OV titers in cancer cells for up to 40 h (Figs 1d and 3c) . This suggests the possibility that these compounds irreversibly inhibit their putative target and/or change the state of the cell, increasing its sensitivity to infection. This may notably explain why we have previously observed *in vivo* activity with **1,** even though we find it is rapidly degraded in serum (Fig. 1b)^10^. While we have not been able to detect **1** in tumors (data not shown), we could detect pyrrole-based **10** and **24** by LC-MRM following intratumoral injection for up to 3 hours (Supplementary Fig. 10). Following intratumoural injection, **28** was detectable in the tumor and serum for up to 10 hours (Supplementary Fig. 11).

Importantly, the selectivity of OVs for cancer cells is generally maintained using these novel compounds. It is unclear whether this is due to the decreased susceptibility of normal cells to the small molecules, or rather, a reflection of the inherent tropism of the viruses. In particular, cancer cells have generally elevated metabolic rates and produce more virus per cell even with non-attenuated viruses^4^,^23^. Hence, in the context of cancer cells, dampened IFN-response may have a more significant impact as has been suggested from mathematical modeling studies^23^,^24^.

One of the properties that has been successfully improved over parent compound **1** is the reduction of electrophilicity as determined by GSH reactivity. Highly electrophilic compounds such as **1** are susceptible to nucleophilic attack and generally less desirable from a pharmacological standpoint. While completely eliminating the electrophilicity of the compounds leads to inactive molecules (Supplementary Table 1), reducing it by employing the pyrrole scaffold does lead to biologically active molecules with substantially improved tolerability. Importantly, similar electrophilic compounds are used clinically for cancer and other applications (e.g. afatinib, mitomycin C, exemestane, esomeprazole, and orlistat)^25^.

In summary, we have developed a new class of well-tolerated compounds that sensitize cancer cells to infection with attenuated viruses *in vitro* and *in vivo*. As such, these have high clinical potential for use in combination with OV and gene therapy strategies for treatment of cancer.

## Experimental Section

### Cell lines

786-0 (human renal carcinoma), A549 (human lung adenocarcinoma), Vero (monkey kidney), CT26 (murine colon carcinoma), 4T1 cells (murine mammary carcinoma), B16F10-LacZ (murine melanoma) cells, and HT29 (human colon carcinoma) were obtained from the American Type Culture Collection (Manassas, VA) and maintained in Dulbecco’s Modified Eagle’s medium (Corning, Manassas, VA) supplemented with 10% fetal bovine serum (Sigma-Aldrich, St Louis, MO) and buffered with 30 mM Hepes (Thermo Fisher Scientific, Waltham, MA). All cell lines were incubated at 37 °C with 5% CO_2_ in a humidified incubator.

### Viruses

#### Oncolytic Rhabodviruses

VSVΔ51 is a recombinant variant of the Indiana serotype of VSV harbouring a deletion of the 51^st^ methionine in the M protein. VSVΔ51 expressing green fluorescent protein (GFP) or firefly luciferase (FLuc) are recombinant derivatives of VSVΔ51 that have been previously described^7^. Maraba MG-1 as described^13^ was obtained from Dr David F. Stojdl. All virus stocks were propagated in Vero cells, purified on Optiprep gradient and titered on Vero cells as previously described^23^.

#### Oncolytic Herpes simplex-1

HSV-1 N212 (an ICPO-deleted oncolytic strain) expressing GFP was obtained from Dr. Karen Mossman and has been described previously^26^. HSV-1 samples were titered on Vero cells. Vero cells (2.5 × 10^5^ cells) were infected with serial dilutions of virus containing samples in 12-well dishes. Cell were incubated at 37 °C for 1 h, after which the inoculum was removed and replaced with fresh culture media. After a 48 h incubation at 37 °C, GFP positive plaques were visualized and counted.

#### Non-replicating vectors

AAV2-luciferase (adeno-associated virus serotype 2 expressing luciferase) was obtained from Dr. Sarah Wootton (University of Guelph) and Ad5-luciferase (adenovirus serotype 5 expressing luciferase) was obtained from Dr. Jack Gauldie (McMaster University).

### Luciferase reporter-based viral titration assay

This assay has previously been described in detail^12^. Briefly, 786-0 cells were seeded in 96-well plates at a density of 3 × 10^4^ cells/100 μL/ well and allowed to adhere over a 24-hour period. Cells were then pre-treated for 4 hours with control vehicle (DMSO) or compound at various concentrations and subsequently infected with VSVΔ51-FLuc at a multiplicity of infection (MOI) of 0.005. Forty hours later, 25 μL of 786-0 supernatant from each well was transferred into corresponding wells containing a confluent monolayer of Vero cells. At the same time, known amounts of virus (starting at 1 × 10^8^ plaque forming units (pfu) and decreasing by 1 log unit to 10 pfu) were added to Vero cells to generate a standard curve. Plates were centrifuged at 1400 rpm for 5 minutes and then incubated for 5 hours at 37 °C. Luciferase expression was then measured and bioluminescence was expressed in mean relative light units (mRLU; SynergyMx Microplate Reader, BioTek). To generate the standard curve, mRLU was plotted against known input pfu. Four-parameter non-linear regression analysis generated a Hill plot from which unknown input pfu (estimate of viral titer) was interpolated. Data transformation was conducted in R. These estimated titers are termed “viral expression units” (VEU). After supernatant transfer as described, cytotoxicity of compounds was assessed by incubating 786-0 cells with alamarBlue^®^ (AbD Serotec) as per the manufacturer’s instructions. After 2.5 hours, fluorescence was measured (530 nm excitation and 590 nm emission) on a Fluoroskan Ascent Microplate Fluorometer (Thermo Scientific, Hudson, NH). Emission values were normalized to that of untreated controls.

### Glutathione stability experiment

Glutathione stability was assessed using an assay adapted from a recently reported method^27^. 250 μL of a 40 mM DMSO stock solution of each compound was added to L-glutathione (15.4 mg, 5 mol equiv.) suspended in 250 μL of DMSO. The resulting mixture was placed in a 37 °C shaker. 10 μL aliquots were removed and quenched in 990 μL of water (containing 0.5% formic acid) at various time points, including at t = 0 min, for analysis by ESI-LC-MS. All ESI-LC-MS analyses were collected on an API2000 LC/MS/MS System (Applied Biosystems) equipped with a turbo-ion spray ESI probe interfaced with a Prominence UFLC (Shimadzu) equipped with a reverse phase BDS Hypersil C18 50 × 2.1 mm column, particle size 3 μm (Thermo Scientific). HPLC/LCMS UV absorption was monitored at 254 nm and 210 nm. Both the compound and the glutathione adduct were identified by MS. Area of the UV peak was recorded for each time point.

### Plasma stability assay

10 mM methanol stock solutions of each analog were prepared and diluted to 1 μM with aqueous 0.1% formic acid. 5 μL of the diluted solution was inserted into a Proxeon nanoelectrospray emitter (Thermo Scientific, Odense, Denmark) and analyzed in positive ion mode via nanoESI MS on a QStarXL hybrid quadrupole time-of-flight mass spectrometer (AB Sciex, Framingham, MA, USA). Product ion spectra were collected for each compound at varying CID collision energies using an ESI voltage of 1000 V, a declustering potential of 30 V and a focusing potential of 120 V. Two fragments were chosen as multiple reaction monitoring (MRM) transitions for each compound with optimized collision energies. The quantitative transition was used to determine the relative quantities of each compound and the confirmatory transition was used to validate the ion signal observed for the first transition (see Supporting Information).

1 mM methanol stock solutions of each analog were prepared and mixed in experimental triplicate with Balb/c mouse plasma (Innovative Research, Novi, MI, USA) that was buffered 1:1 with phosphate buffered saline (PBS, pH = 7.4). The compounds were multiplexed into sets of three and added to a final concentration of 10 μM in a total volume of 400 μL. Immediately upon mixing, 200 μL of the sample mixture was quenched with 300 μL of aqueous formic acid (5%) to prevent further analog degradation. The remaining 200 μL of sample was incubated at 37 °C for 3 hours and quenched in an identical fashion^28^. The quenched samples were passed through 3 kDa Amicon molecular weight cut off filters (Millipore, Billerica, MA, USA) by centrifugation at 14,000 rpm for 15 minutes. 20 μL samples of the filtrates were subjected to LC-MRM analysis using a Qtrap 4000 (AB Sciex, Framingham, MA, USA) hybrid triple quadrupole linear ion trap mass spectrometer with an ion spray voltage of 5000 V and a declustering potential of 25 V. The MS was equipped with a Turbo V ion spray source coupled to a Dionex Ultimate3000 HPLC (Thermo Fisher Scientific, Waltham, MA, USA). Fritted fused silica columns (200 μm ID) (Molex, Lisle, IL, USA) were packed with 5 μm Magic C18 (MICHROM Bioresources Inc., Auburn, CA, USA) reversed-phase beads to a length of 5 cm using an in-house high-pressure vessel. Chromatographic separation employed a linear gradient using reversed phase solvents (water and acetonitrile both containing 0.1% formic acid) over 10 minutes (see Supporting Information). Automatic quantitation was achieved using MultiQuant software (AB Sciex, Framingham, MA, USA) by integrating the peak areas of the quantitative MRM transition extracted ion chromatogram. The plasma stability of each compound was calculated as a percentage of the compound ion signal detected after 3 hours of plasma incubation relative to the original amount.

### ELISA

786-0 cells were seeded at 3.0 × 10^5^ cells per well in 12-well plates. The following day, the cells were pre-treated with 60, 50, 50 and 95 μM of compound **1**, **2**, **10** and **28** respectively. Two hours following pre-treatments the cells were infected with VSV∆51-GFP at MOI 3. Supernatants were collected 16 hours post infection, and an ELISA was performed using VeriKine™ Human IFN Beta ELISA Kit (PBL assay science, Piscataway, NJ).

### Quantitative real-time PCR

786-0 cells were seeded at 1.0 × 10^6^ cells/well in 6-well plates. The following day, the cells were pre-treated with 60, 50, 50 and 95 μM of compound **1**, **2**, **10** and **28** respectively. Two hours following pre-treatments the cells were infected with VSV∆51-GFP at MOI 3. 16 hours post infection the cells were lysed and RNA extraction was performed using RNeasy^®^ Mini Kit (Qiagen, Valencia, CA). RNA was converted to cDNA with RevertAid H Minus First Strand cDNA Synthesis Kit (Thermo Fisher Scientific, Vilnius, Lithuania). Real-time PCR reactions were performed with QuantiTect^®^ SYBR^®^ Green PCR Kit (Qiagen, Valencia, CA) on a 7500 Fast Real-Time PCR system (Applied Biosystems, Foster City, CA). Gene expression was normalized to GAPDH and fold induction was calculated relative to the untreated/uninfected samples for each gene using the Pfaffl method^29^.

### *Ex vivo* studies

Balb/c mice were implanted with CT26-WT (murine colon carcinoma) cells. Mice were sacrificed 24 days later, after tumors had reached at least 10 mm × 10 mm in size. Tumor, lung, spleen, brain, and abdominal muscle tissue were extracted from the mice, cut into 2 mm thick slices and cored into 2 mm × 2 mm pieces via punch biopsy. Each tissue core was incubated in 1 mL of Dulbecco’s Modified Eagle’s Medium (DMEM) supplemented with 10% fetal bovine serum, 30 mM HEPES and 2.5 mg/L amphotericin B, in a 37 °C, 5% CO_2_ humidified incubator. In order to assess the viability of each core, alamarBlue^®^ was added to each well for a 4-hour incubation period. Viable cores were selected and treated with various concentrations of **1** and analogs. The cores were then infected with VSV∆51 expressing a GFP transgene (VSV∆51-GFP) four hours post treatment. GFP pictures were taken for each core 24 hours post infection. Cores and supernatants were collected 30 hours post infection and titered by plaque assay.

### *In vivo* studies

#### Dose escalation studies

Nine-week-old Balb/c mice were intraperitoneally administered various doses of compounds **1**, **10**, **24**, or **28** dissolved in DMSO (approximately 50 μL). Weight loss and other outward signs of toxicity (piloerection, malaise, quiet behaviour) were recorded over a 10 (**1**, **10**) or 18-day (**24**, **28**) period.

#### *In vivo* enhancement of virus replication

Nine-week-old female Balb/c mice were given subcutaneous tumors by injecting 3 × 10^5^ syngeneic CT26 cells suspended in 100 μl serum-free DMEM. Eleven days post-implantation (when tumors were approximately 5 mm × 5 mm), mice were treated with 40 mg/kg of compound **28** dissolved in DMSO or vehicle control administered intratumorally (approximately 30 μL). Four hours later, mice were treated with an intratumoral injection of VSVΔ51-FLuc (1 × 10^8^ plaque-forming units). For *in vivo* imaging, an IVIS (Perkin Elmer, Waltham, MA) was used as described previously^30^. Briefly, 200 μl of a 10 mg/mL D-Luciferin (Biotium Hayward, CA) solution in PBS (Corning, Manassas, VA) was administered to mice intraperitoneally. Five minutes later, mice were anaesthetized using 3% isoflurane and imaged according to the manufacturer’s instructions. For quantification of luminescence described in Fig. 6c, bioluminescent signal intensities were measured using Living Image^®^ v2.50.1 software. Background intensities were measured using the software and subtracted from user-defined regions of interest (ROIs) that were manually delineated around the tumor for each mouse.

#### CT26 tumor model

Six-week-old female Balb/c mice were given subcutaneous tumors by injecting 3 × 10^5^ syngeneic CT26 cells suspended in 100 μl serum-free DMEM. Eleven days post-implantation (when tumors were approximately 5 mm × 5 mm), mice were treated with 50 mg/kg of compound **10** dissolved in DMSO or vehicle control administered intraperitoneally (approximately 30 μL). Four hours later, mice were treated with an intratumoral injection of VSVΔ51-FLuc (1 × 10^8^ plaque-forming units). **10** or vehicle was readministered on day 13 and 15 post-implantation. Tumor dimensions were measured with electronic calipers. Tumor volumes were calculated as (width^2^ × length)/2. Mice were euthanized when tumor volume exceeded 1600 mm^3^. Initial tumor sizes measured on day 11 were used to calculate relative tumor size.

#### HT29 tumour model

Six-week-old CD1 nude mice were given subcutaneous tumors by injecting 1 × 10^6^ syngeneic HT29 cells suspended in 100 μl serum-free DMEM. When tumors grew to approximately 5 mm × 5 mm (between 18–25 days post-implantation), mice were treated with 40 mg/kg of compound **28** dissolved in DMSO or vehicle control administered intraperitoneally (approximately 30 μL). Four hours later, mice were treated with an intratumoral injection of VSVΔ51-FLuc (1 × 10^8^ plaque-forming units). Tumor dimensions were measured every other day with electronic calipers. Tumor volumes were calculated as (width^2^ × length)/2. Mice were euthanized when tumor volume exceeded 1600 mm^3^. Initial tumor sizes measured on the day of treatment were used to calculate relative tumor size.

#### Pharmacokinetics

##### Short time course

Nine-week-old female Balb/c mice were given subcutaneous tumors by injecting ~3 × 10^5^ syngeneic CT26 cells suspended in 100 μl serum-free DMEM. Nineteen days post-implantation, mice were intratumorally administered 50 mg/kg of compound **10** or **24** dissolved in DMSO (approximately 50 μL). Tumors were excised after 0 h, 15 minutes, 1 h and 3 h and homogenized immediately at 30 Hz for 5 minutes with a TissueLyser II (Qiagen). Samples were the centrifuged (20,000 rpm, 30 s, 4 °C) and homogenized again in 500 μL PBS. After another round of centrifugation, the supernatant was passed through Amicon Ultra−0.5 mL 3 kDa molecular weight cut off filters (EMD Millipore) by centrifugation, and the filtrate was quantified by LC-MRM.

##### Long time course

Nine-week-old female Balb/c mice were given subcutaneous tumors by injecting ~3 × 10^5^ syngeneic CT26 cells suspended in 100 μl serum-free DMEM. Nineteen days post-implantation, mice were intratumorally administered vehicle control (DMSO), or **28** dissolved in DMSO (approximately 30 μL). Tumors treated with **28** were excised after 1 h, 3 h, 10 h and 24 h. Tumors treated with vehicle alone were excised after 3 h and 24 h. Tumor homogenization and centrifugation was conducted in an identical fashion to that described in the short time course (above). At the time of tumor excision, peripheral blood was also collected and allowed to clot at room temperature for at least 30 minutes. Samples were centrifuged at 20,000 rpm for 10 minutes at 4 °C, and the supernatant (serum) was collected. Serum was diluted 5x with 0.1% formic acid in water and centrifuged through Amicon Ultra−0.5 mL 3 kDa molecular weight cut off filters (EMD Millipore). For both tumor and serum samples, 25 μL of the filtrate was mixed with 5 μL of a 6 μM solution of caffeine in water (as a standard to allow relative quantitation) and quantified by LC-MRM.

All experiments were reviewed and approved by the University of Ottawa Animal Care Committee (ACC) and were performed in accordance with the University of Ottawa Animal Care and Veterinary Services guidelines for animal care under protocols OGHRI-58 and OHRI-2265.

## Additional Information

**How to cite this article**: Dornan, M. H. *et al.* First-in-class small molecule potentiators of cancer virotherapy. *Sci. Rep.* **6**, 26786; doi: 10.1038/srep26786 (2016).

## Supplementary Material

Supplementary Information
