# Supplementary material for: First-in-class small molecule potentiators of cancer virotherapy
Source: Sci Rep. 2016 May 26;6:26786. doi: 10.1038/srep26786 (PMC4880900; doi:10.1038/srep26786)
Supplement: Supplementary Information [file srep26786-s1.pdf]

SUPPLEMENTARY INFORMATION

First-in-class small molecule potentiators of cancer virotherapy

Mark H. Dornan,[1,5] Ramya Krishnan,[2,3,5] Andrew M. Macklin,[4,5] Mohammed Selman,[2,3] Nader El Sayes,[2,3] Hwan Hee Son,[2,3] Colin Davis,[2,3] Andrew Chen,[2] Kerkeslin Keillor,[1] Penny Le,[1] Christina Moi,[1] Paula Ou,[2,3] Christophe Pardin,[1] Carlos R. Canez,[4] Fabrice Le Boeuf,[2] John C. Bell,[2,3] Jeffrey C. Smith,*[4] Jean-Simon Diallo,*[2,3] Christopher N. Boddy *[1]

[1] Departments of Chemistry and Biomolecular Sciences, University of Ottawa, Ottawa, Ontario, Canada

[2] Centre for Innovative Cancer Research, Ottawa Hospital Research Institute, Ottawa, Ontario, Canada

[3] Department of Biochemistry, Microbiology and Immunology, University of Ottawa, Ontario, Canada

[4] Department of Chemistry and Institute of Biochemistry, Carleton University, Ottawa, Ontario, Canada

[5] These authors contributed equally to this work

* email: jeff.smith@carleton.ca; jsdiallo@ohri.ca; cboddy@uottawa.ca

Table of Contents

Supplementary Figures 2

Figure S1 Analogs of 1 enhance oncolytic VSV spread in murine melanoma cells 3

Figure S2 Analogs of 1 enhance oncolytic VSV spread in murine colon cancer cells 4

Figure S3 Analogs of 1 enhance oncolytic VSV activity in murine breast cancer cells 5

Figure S4 Analogs of 1 enhance oncolytic HSV-1 spread in human renal carcinoma cells 6

Figure S5 1 and Pyrrole-based analog 10 enhance transduction of non-replicating gene therapy vectors in human lung carcinoma cells 7

Figure S6 VSV Titers for all concentrations tested in *ex vivo* experiment 8

Figure S7 Compound 10 enhances VSVΔ51 oncolytic activity in a resistant syngeneic tumor model 9

Figure S8 Compound 1 and analogs inhibit the production of IFNβ and interferon-stimulated genes .. 10

Figure S9 Interferon-induced antiviral response is overcome by 1 and its analogs. 11

Figure S10 Compound 10 and 24 can be detected in the tumor following intratumoral injection 12

Figure S11. Compounds 28 can be detected in the tumor and serum following intratumoral injection. 13

Supplemental Table S1: Analog activity, toxicity and stability 14

Synthetic methods and characterization 20

Spectra and titers: ^{1}H NMR, ^{13}C NMR, MRM transitions, VEU titer 35

MRM Transition Data 87

References 89

Supplementary Figures

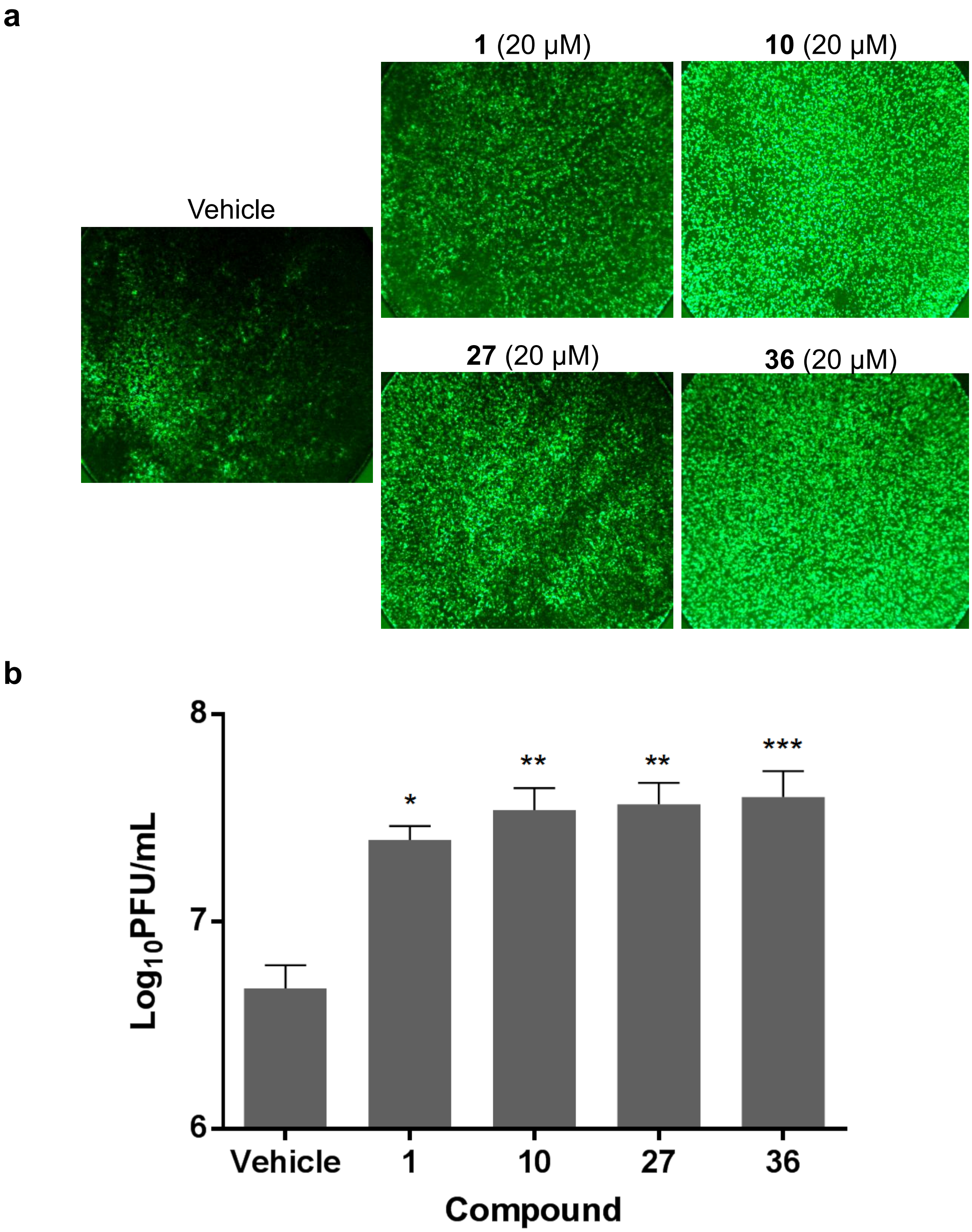

Supplementary Figure S1. Analogs of 1 enhance oncolytic VSV spread in murine melanoma cells. Murine melanoma (B16F10-LacZ) cells were treated with vehicle, **1**, **10**, **27**, or **36** for 4h at the specified concentrations. 4 hours later, cells were infected with VSVΔ51-GFP at MOI 0.001. (A) Virus replication was assessed by fluorescence microscopy 24 hours post-infection. (B) Samples were titered 48 hours post-infection. *p = 0.0392, **p = 0.0027 for 10 vs vehicle and 0.0015 for 27 vs vehicle, ***p = 0.0007 for 36 vs vehicle (one-way ANOVA with Dunnett's multiple comparisons test), Error bars represent standard error.

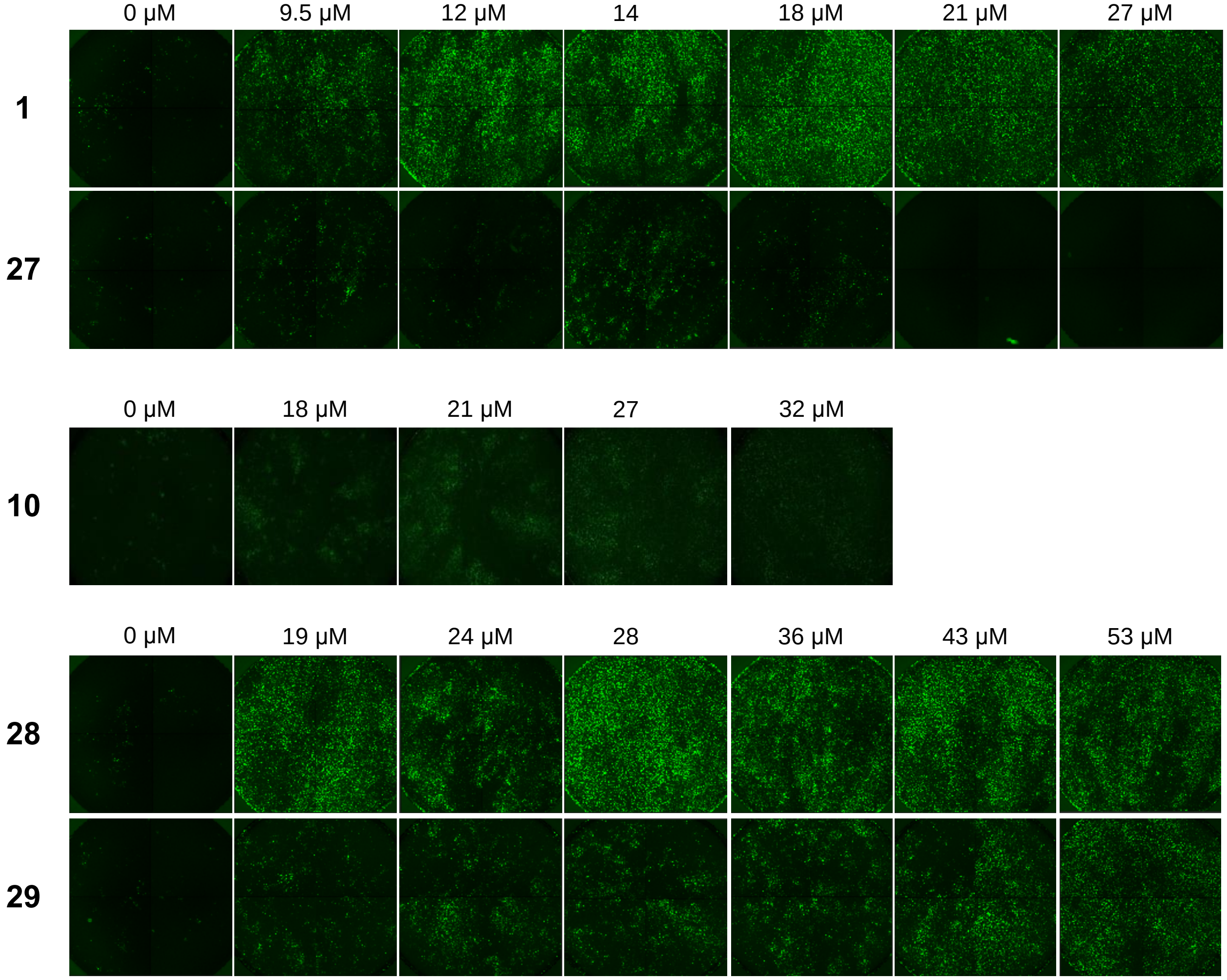

Supplementary Figure S2. Analogs of 1 enhance oncolytic VSV spread in murine colon cancer cells. Murine colon carcinoma (CT26-WT) cells were treated with vehicle, **1**, **10**, **27**, **28**, or **29** for 4h at the specified concentrations. 4 hours later, cells were infected with VSVΔ51-GFP at MOI 0.005. Virus replication was assessed by fluorescence microscopy 24 hours post-infection.

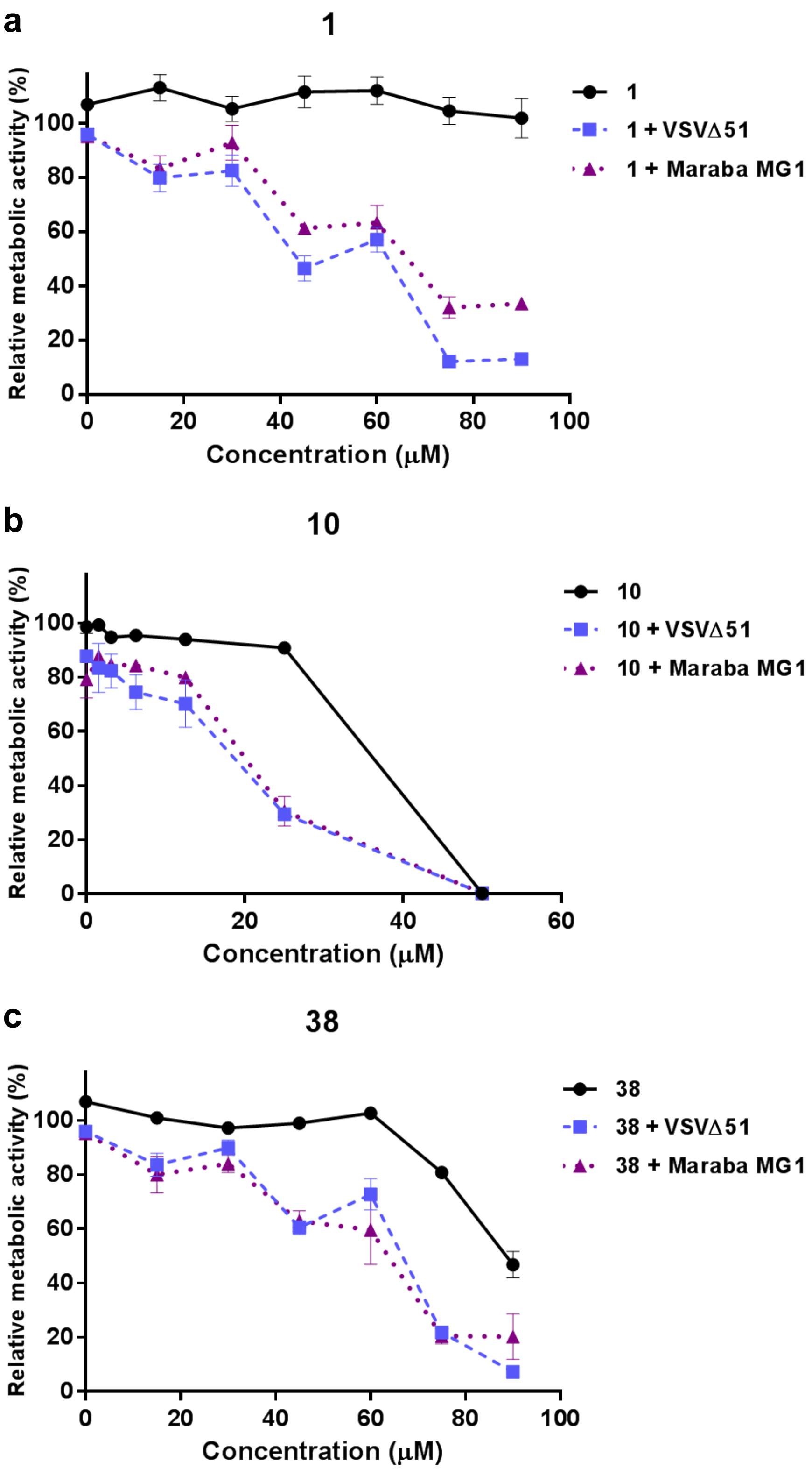

Supplementary Figure S3. Analogs of 1 enhance oncolytic VSV activity in murine breast cancer cells. Murine mammary carcinoma (4T1) cells were treated with vehicle, (A) **1**, (B) **10**, or (C) **38** for 4h at the specified concentrations. 4 hours later, cells were infected with VSVΔ51 or Maraba MG1 at MOI 0.005. 40 hours post-infection, cytotoxicity was assessed by incubating samples with alamarBlue® for 2 h at 37°C before measuring fluorescence (530nm excitation, 590 nm emission). Values were normalized to that of untreated controls.

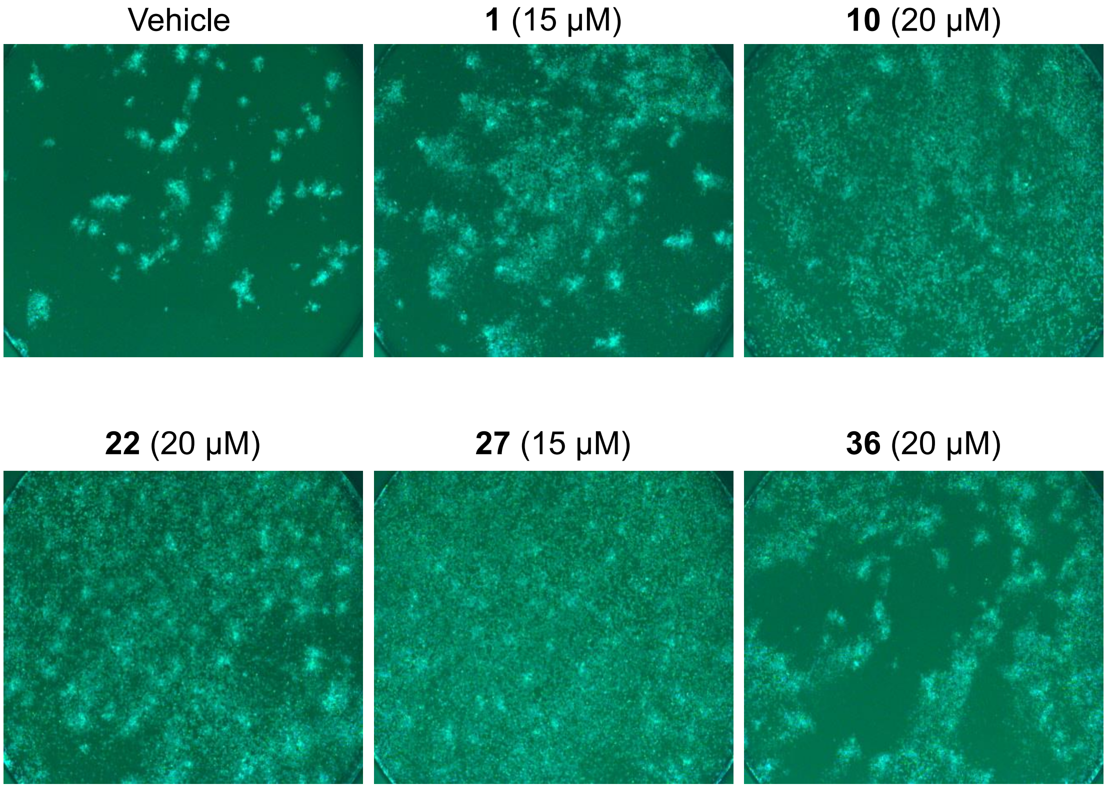

Supplementary Figure S4. Analogs of 1 enhance oncolytic HSV-1 spread in human renal carcinoma cells. Human renal carcinoma (786-0) cells were treated with vehicle, **1**, **10**, **22**, **27**, or **36** for 4h at the specified concentrations. 4 hours later, cells were infected with ICP0-null HSV-N212eGFP at MOI 0.005. eGFP fluorescence was detected 48h after HSV infection.

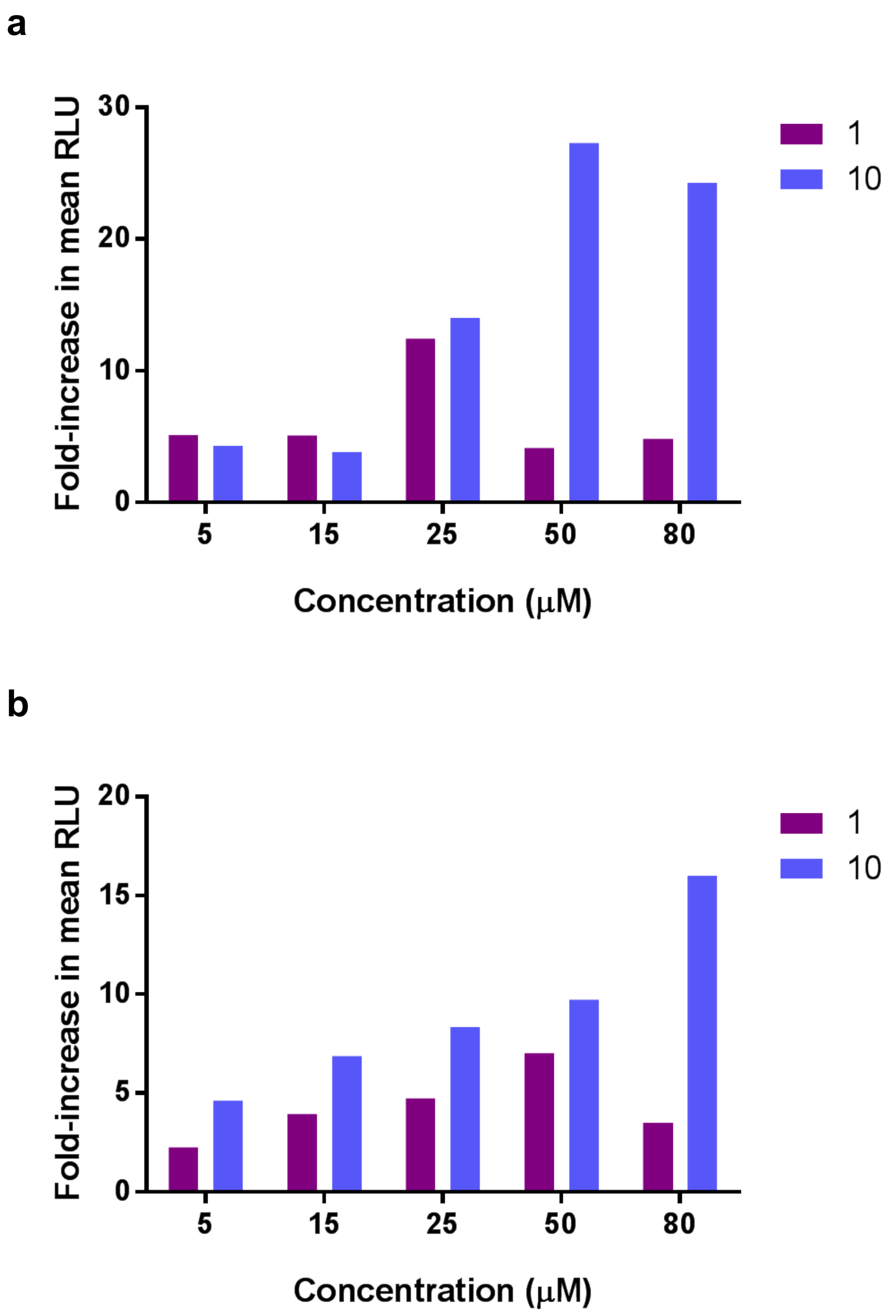

Supplementary Figure S5. 1 and Pyrrole-based analog 10 enhance transduction of non-replicating gene therapy vectors in human lung carcinoma cells. Human lung carcinoma (A549) cells were treated with **1** or **10** analogs at various concentrations. 4 hours later, cells were infected with A) replication defective adeno-associated virus expressing firefly luciferase (AAV2-luc) or B) replication defective adenovirus expressing firefly luciferase (Ad5-luc) at an MOI of 1. 24 hours later, luciferase activity was measured. Data is represented as the fold increase in mean relative light units of treated samples versus untreated controls

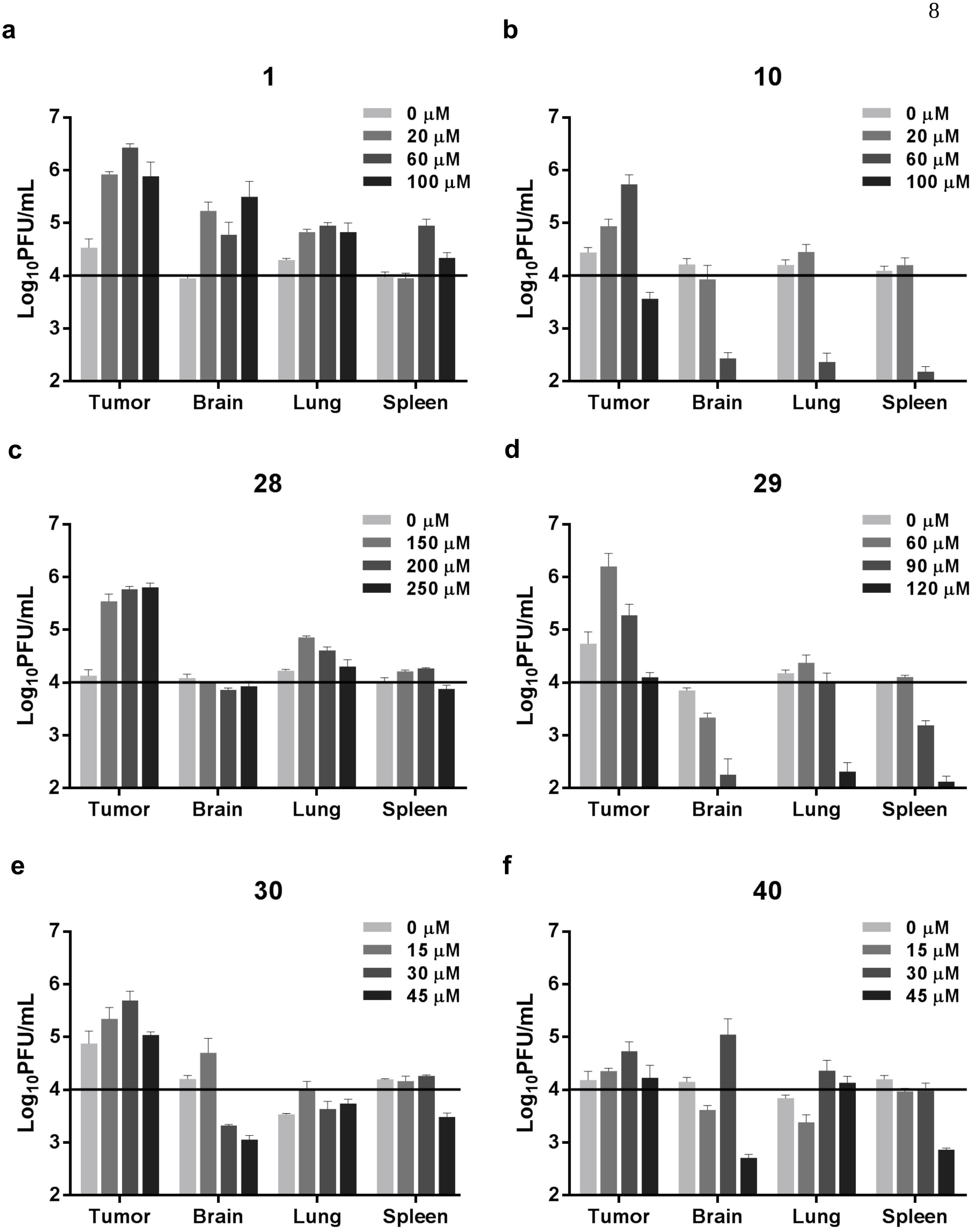

Supplementary Figure S6. VSV Titers for all concentrations tested in *ex vivo* experiment. Increasing doses of 1 and its analogs were tested as described in Figure 3, which depicts the most effective concentration in tumor tissues for each analog. See Methods section for more information.

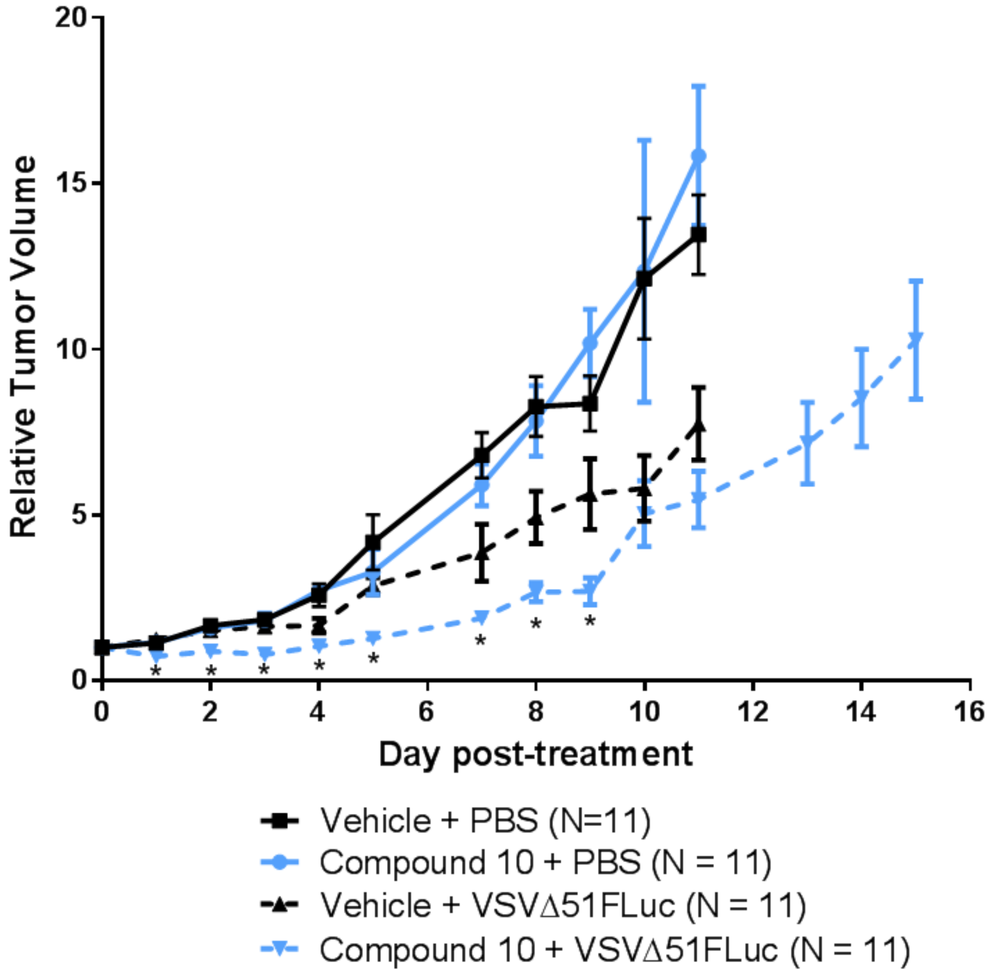

Supplementary Figure S7. Compound 10 enhances VSVΔ51 oncolytic activity in a resistant syngeneic tumor model. VSVΔ51-resistant CT26 cells were subcutaneously engrafted into female Balb/c mice. After 11 days, mice were given 50 μL of vehicle (DMSO), or 50 mg/kg of **10** by intraperitoneal injection. Four hours later, mice were treated with $1x10^8$ plaque-forming units of VSVΔ51-Fluc by intratumoral injection. Vehicle or or **10** was readministered on day 2 and 4 post-initial treatment. Pooled results from two separate experiments are shown. Average tumor volumes for each treatment group are shown and error bars correspond to standard error. Tumor volume curves are terminated when the first mouse in each group is euthanized. Average tumor volumes of vehicle + VSVΔ51-Fluc and **10** + VSVΔ51-Fluc treated groups were compared with a Student's t-test for each day. * $P < 0.05$.

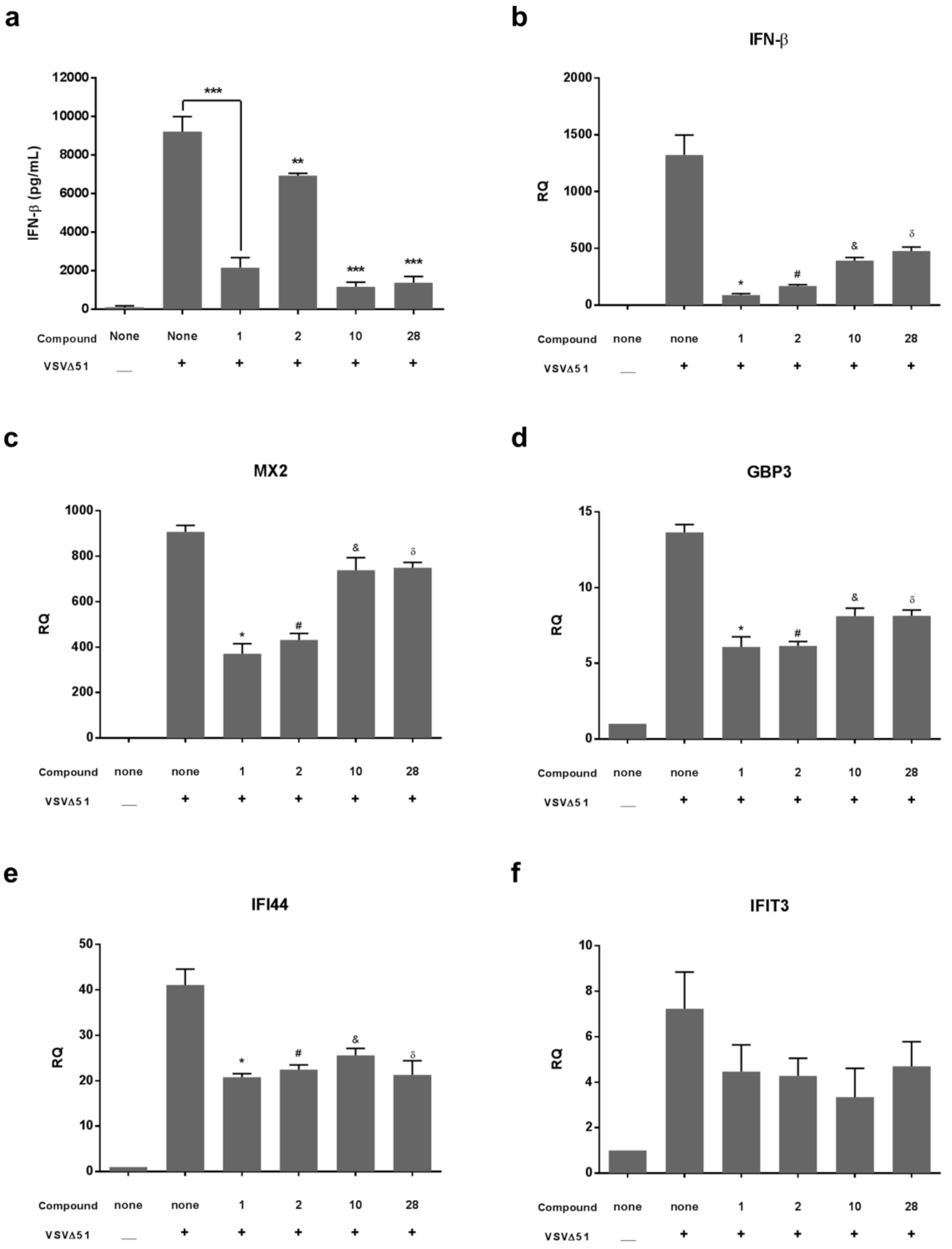

Supplementary Figure S8. Compound 1 and its analogs inhibit the production of IFNβ and interferon-stimulated genes (ISGs). (a) 786-0 cells were pre-treated with compounds 1, 2, 10 and 28 before infection with VSVΔ51-GFP for 16 hours. Amount of IFN□ in the supernatant was measured by sandwich ELISA. *** $P < 0.0001$, ** $P = 0.0091$ (One-Way ANOVA), n=3 (biological replicates). (b-f) Quantitative RT-PCR for IFN□ and ISG expression in 786-0 cells pre-treated with 1, 2, 10 and 28 before infection with VSVΔ51-GFP for 16 hours. (A/IFNb) * $P < 0.0001$, # $P < 0.0001$, & $P < 0.0001$, □ $P < 0.0001$. (B/MX2) * $P < 0.0001$, # $P < 0.0001$, & $P < 0.0195$, □ $P < 0.0290$. (C/GBP3) * $P < 0.0001$, # $P < 0.0001$, & $P < 0.0001$, □ $P < 0.0001$. (D/IFI44) * $P < 0.0001$, # $P < 0.0001$, & $P < 0.0001$, □ $P = 0.0003$ (One-Way ANOVA), n=3 (biological duplicates were pooled and the RT-PCR was done in triplicate). This experiment was performed twice with similar results. Representative results from one experiment are shown.

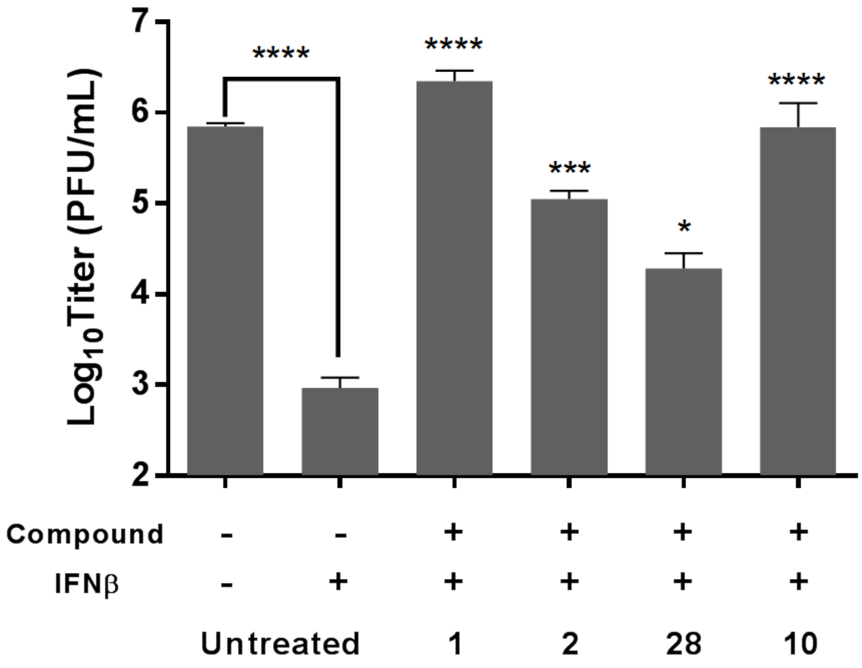

Supplementary Figure S9. Interferon-induced antiviral response is overcome by 1 and its analogs. Human renal carcinoma (786-0) cells were co-treated with compound and IFNβ for 4 hours and then challenged with VSVΔ51-GFP at MOI 0.01. Samples were titered 48 post-infection. *p = 0.0109, ***p = 0.001, ****p = <0.0001 (one-way ANOVA with Dunnett's multiple comparisons test), Error bars represent standard error.

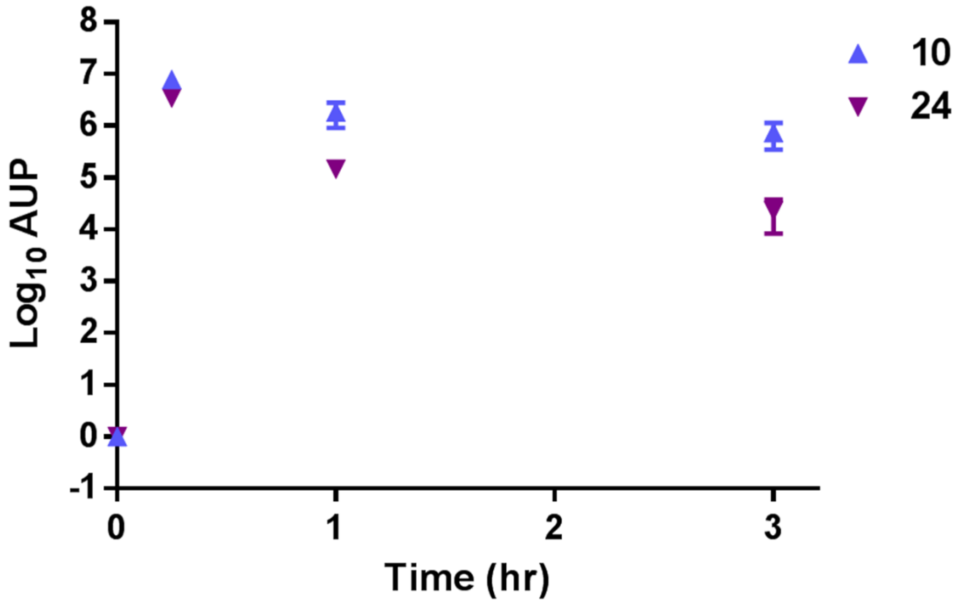

Supplementary Figure S10. Compounds 10, and 24 can be detected in the tumor following intratumoral injection. Mice (N = 1 per timepoint) bearing subcutaneous CT26 tumors were injected intratumorally with 50 mg/kg of 10 or 24. Tumors were excised at various times post-injection and analysed for the presence of compound by LC-MRM. Area under the peak (AUP) for the parent compound(s) are shown. Error bars represent the standard deviation of 3 technical replicates.

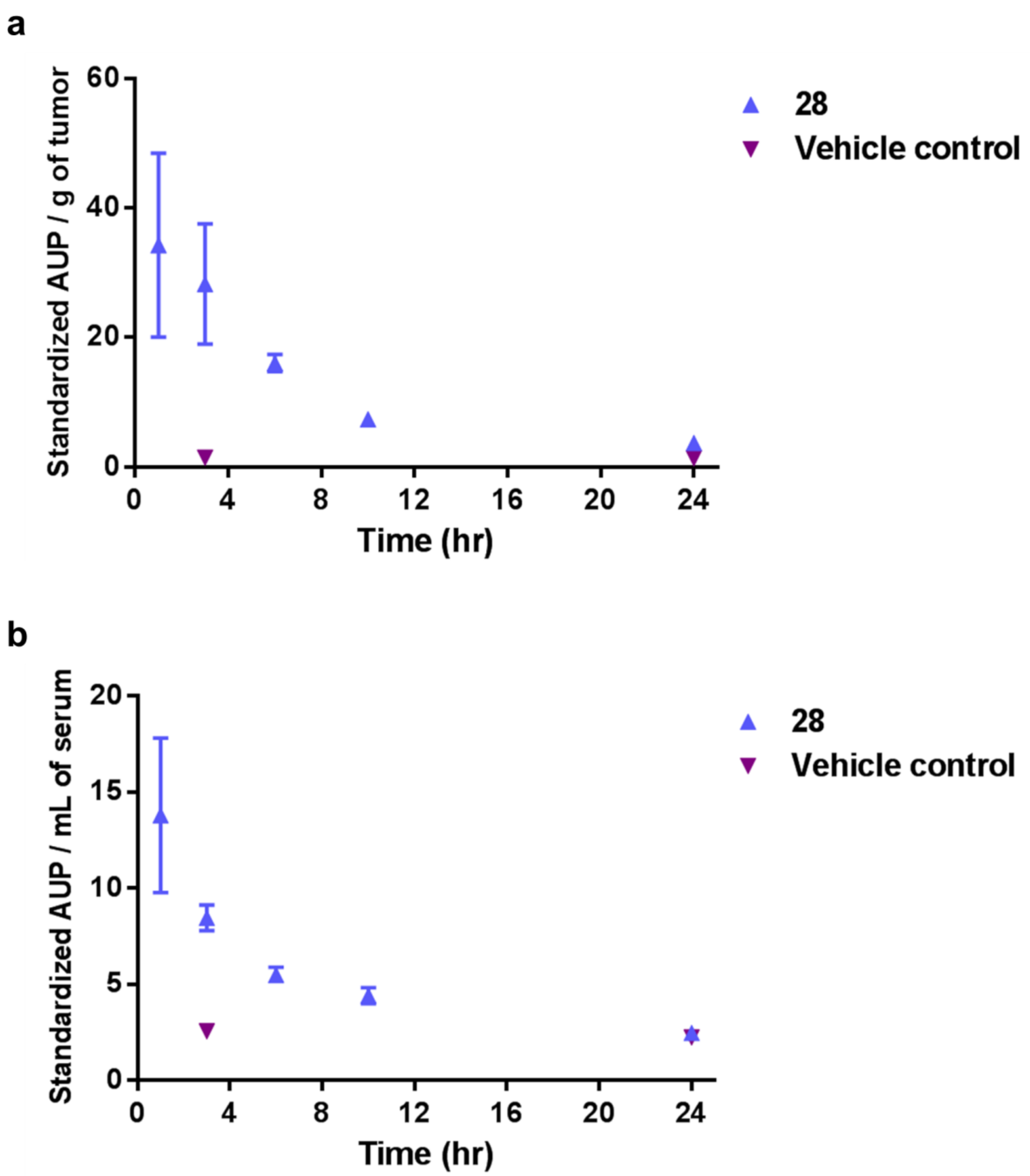

Supplementary Figure S11. Compounds 28 can be detected in the tumor and serum following intratumoral injection. Mice bearing subcutaneous CT26 tumors were injected intratumorally with 40 mg/kg of 28 (N = 3 per timepoint) or vehicle control (N =1 for 3h and 24h timepoints). (a) Tumors and were excised and (b) serum was collected at various times post-injection and analysed for the presence of compound by LC-MRM. Standardized area under the peak (AUP) for the parent compound are shown. Error bars represent the standard error for 3 biological replicates.

Supplementary Table S1. Analog activity, toxicity and stability.

ID	Structure	Peak fold enhancement[a]	PFE dose (μM)[b]	LD_{50} (μM)	LD_{50} with virus (μM)	GSH half-life (min.)	Plasma stability at 3 hours (% remaining)
1	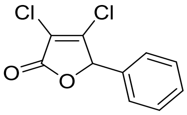	1910	60	79	16	< 5	0
2	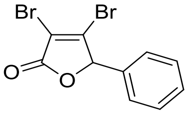	705	72	87	50	< 5	0
3	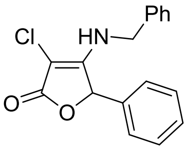	365	96	140	140	NR[c]	65.6 ± 6.5
4	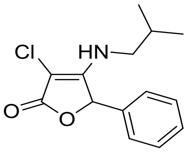	345	80	90	90	NR	0
5	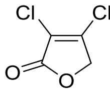	515	36	41	27	< 5	0
6	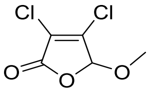	400	60	73	51	< 5	0
7	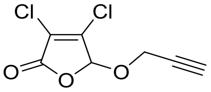	575	60	52	17	< 5	0
8	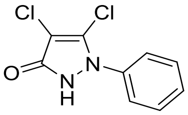	NE[d]	-	>180	>180	NR	88.3 ± 9.3
9	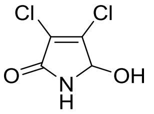	1280	120	148	87	117	0
10	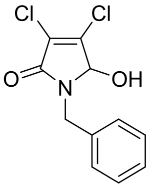	555	48	67	51	32	19.8 ± 0.4
11	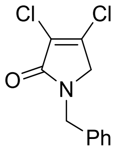	55	240	332	332	64	42.5 ± 9.6

12	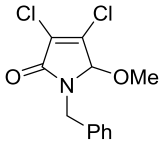	535	180	206	203	118	47.6 ± 1.4
13	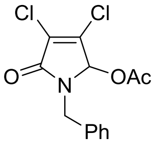	305	60	61	45	21	0
14	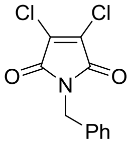	75	96	91	85	< 5	70.2 ± 8.4
15	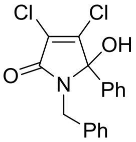	NE	-	66	66	340	14.9 ± 7.1
16	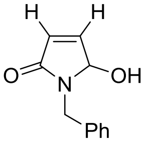	NE	-	> 360	> 360	NR	98.2 ± 3.7
17	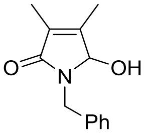	NE	-	> 360	> 360	NR	82.0 ± 10.2
18	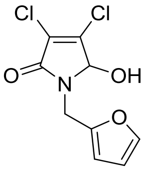	475	60	67	28	45	54.1 ± 5.1
19	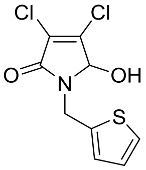	975	60	64	27	40	50.1 ± 10.5
20	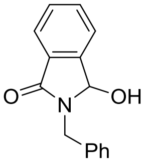	NE	-	>360	>360	NR	102.9 ± 1.6
21	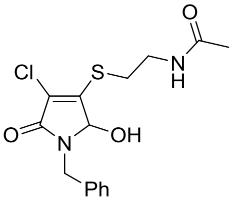	55	504	630	567	NR	102.7 ± 10.8

22	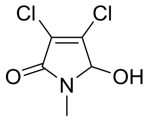	895	96	119	76	68	72.0 ± 3.0
23	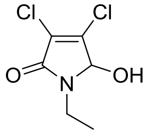	1105	120	171	89	-	ND
24	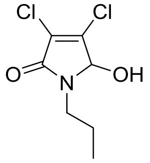	915	120	174	96	61	91.6 ± 5.2
25	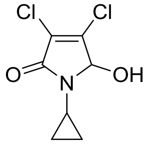	1415	80	127	51	53	54.8 ± 3.6
26	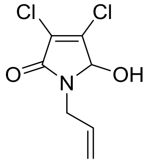	995	96	110	66	46	64.8 ± 7.7
27	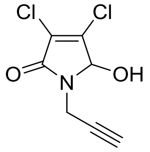	40	48	100	87	21	9.0 ± 1.4
28	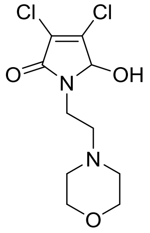	1910	80	153	55	96	38.9 ± 5.2
29	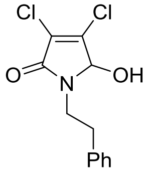	975	72	74	27	74	57.6 ± 6.6
30	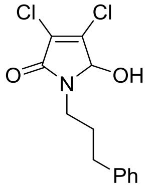	1090	32	36	20	50	42.9 ± 7.2

31	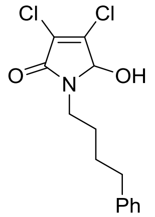	495	40	40	34	72	40.1 ± 9.8
32	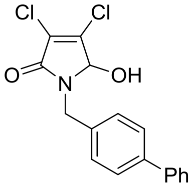	210	27	28	5	24	ND[e]
33	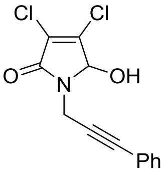	575	18	18	12	24	0
34	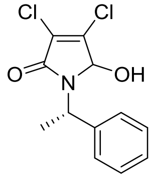	630	72	74	6	31	48.0 ± 16.5
35	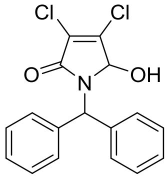	265	27	36	23	43	63.8 ± 3.2
36	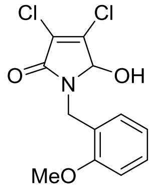	55	72	56	56	34	28.2 ± 2.6
37	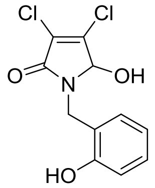	1070	48	58	38	41	0.7 ± 0.1
38	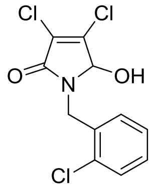	670	216	215	107	32	25.7 ± 2.9

39	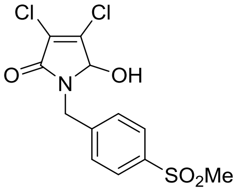	975	60	> 90	25	34	41.4 ± 5.4
40	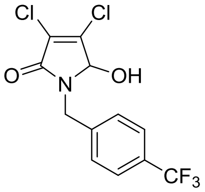	2005	27	36	13	32	15.3 ± 2.5
41	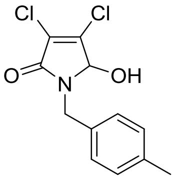	365	40	39	30	35	51.4 ± 8.2
42	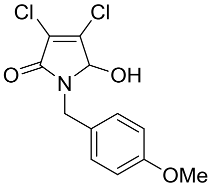	190	40	55	17	40	49.1 ± 12.4
43	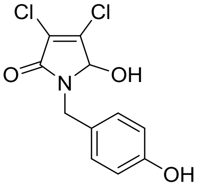	285	60	> 90	45	69	58.3 ± 0.6
44	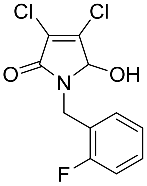	155	60	63	39	31	45.9 ± 8.1
45	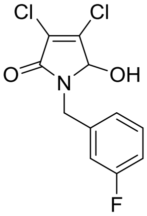	115	48	43	37	31	54.2 ± 4.2
46	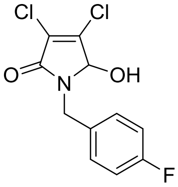	170	40	42	36	32	23.1 ± 0.8

47	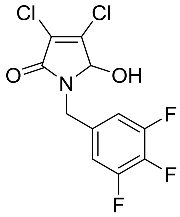	190	40	36	35	35	22.7 ± 8.4
48	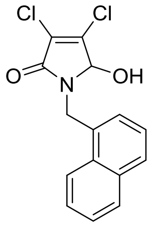	1240	32	38	24	14	36.5 ± 7.6
49	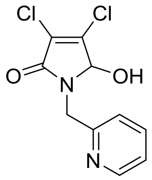	590	96	131	67	64	44.6 ± 1.2
50	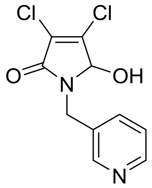	800	60	85	29	54	39.6 ± 2.6
51	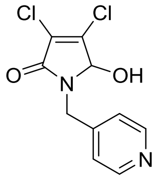	590	72	89	28	53	44.0 ± 1.0

[a] Peak fold enhancement is the peak fold change in viral expression units (VEU) relative to vehicle-treated infected samples. [b] PFE dose is the drug concentration used to achieve the peak fold change in VEU. [c] No reaction with glutathione observed. [d] No enhancement of VEU. [e] No data.

**Gene**	**Forward primer**	**Reverse primer**	**Product size (bp)**
IFIT3	5'-GCACAGACCTAACAGCACCC-3'	5'-TTGGTGACCTCACTCATGATGGC-3'	134
IFI 44	5'-CCCATCGCTGAAGGACAGAA-3'	5'-CACATGTACCACACCAGCGT-3'	136
GBP3	5'-ACTGGTGGCGAATCCAGAAG-3'	5'-GCCCAGAGAGAAGCCCTTATT-3'	145

MX2	5'-GAACGTGCAGCGAGCTTGTC-3'	5'-AAGGCTTGTGGGCCTTAGAC-3'	105

Supplementary Table S2. RT-qPCR Primers

Synthetic methods and characterization

General information

All reactions were performed in oven-dried glass round-bottom flasks equipped with magnetic stir bars. Purification of reaction products was carried out by flash column chromatography using silica gel. Analytical thin-layer chromatography (TLC) was performed using silica gel 60 F_{254} plates from EMD. ^{1}H and ^{13}C NMR spectra were recorded on a Bruker 400 MHz and 100 MHz, respectively, at ambient temperature. Spectra are recorded in parts per million using residual solvent as the internal standard ($(CD_3)_2SO$ at 2.50 ppm, $CDCl_3$ at 7.26 ppm and CD_3OD at 3.31 ppm for ^{1}H NMR and $(CD_3)_2SO$ at 39.52 ppm, $CDCl_3$ at 77.16 ppm and CD_3OD at 49.00 ppm for ^{13}C NMR.) ^{1}H NMR data was reported as: multiplicity (br = broad, s = singlet, d = doublet, t = triplet, q = quartet, quin. = quintet, sext. = sextuplet, sept = septuplet, m = multiplet), integration and coupling constant(s) in Hz. High-resolution mass spectrometry was performed in positive ion mode via nanoESI MS using a QStarXL hybrid quadrupole time-of-flight mass spectrometer.

Materials

Unless otherwise noted, all commercially available materials were purchased from commercial sources and used without further purification.

3,4,5-Trichloro-5*H*-furan-2-one

Mucochloric acid (5 g, 26.7 mmol) was added to 17.8 mL of thionyl chloride. A catalytic amount of dimethylformamide (3 drops) was added and the mixture was heated at 70 °C for 5 hours. The mixture was cooled and excess thionyl chloride was evaporated under vacuum. Toluene (100 mL) was added to the flask and evaporated under vacuum 3 times to azeotrope any residual thionyl chloride. The crude product was isolated as a yellow oil was used in subsequent reactions without further purification.

General procedure A

An amine (2.2 equiv.) was added to dioxane (0.3 M). 3,4,5-Trichloro-5*H*-furan-2-one (1.0 equiv.) was added dropwise and the mixture was allowed to stir at room temperature for 16 hours. The reaction mixture was quenched with $NH_4Cl_{(aq)}$ and extracted 3 × with EtOAc. The organic fractions were washed with brine, dried over Na_2SO_4 and concentrated. The desired compound was purified by silica column chromatography.

General procedure B

3,4-dichloro-5-phenyl-2,5-dihydrofuran-2-one (1 equiv.) was added to DMF (0.05 M) and an amine (3 equiv.) was added. The reaction was allowed to stir at room temperature for 16 hours. $NH_4Cl_{(aq)}$ was added to quench the reaction and the mixture was extracted 3 × with EtOAc. The organic fractions were combined and washed with brine, dried over Na_2SO_4 and concentrated. The desired compound was purified with silica column chromatography.

3,4-dichloro-5-phenyl-2,5-dihydrofuran-2-one, Compound 1

Mucochloric acid (2.00 g, 1 equiv.) was dissolved in benzene (10 mL) in a round-bottom flask equipped with a stir bar. $AlCl_3$ (2.37 g, 1.5 equiv.) was added and the solution was allowed to stir for 3 days. The mixture was poured over an HCl / ice mixture (5.7 mL conc. HCl). The mixture was extracted 3 × with Et_2O and the combined organic fractions were washed with brine, dried over Na_2SO_4 and concentrated. The desired compound was recrystallized from ethanol. ^{1}H NMR (400 MHz, DMSO) δ 7.51 – 7.41 (m, 5H), 6.40 (s, 1H). ^{13}C NMR (100 MHz, DMSO) δ 165.25, 152.50, 132.05, 130.34, 129.21, 127.83, 119.91, 83.10. HRMS (ESI): Exact mass calculated for $C_{10}H_6Cl_2O_2$ $[M + H]^+$: 228.9823. Found: 228.9944

3,4-dibromo-5-phenyl-2,5-dihydrofuran-2-one, Compound 2

Mucobromic acid (2.00 g, 1 equiv.) was dissolved in benzene (10 mL) in a round-bottom flask equipped with a stir bar. $AlCl_3$ (1.56 g, 1.5 equiv.) was added and the solution was allowed to stir for 3 days. The mixture was poured over a HCl / ice mixture (5.7 mL conc. HCl). The mixture was extracted 3 × with Et_2O and the combined organic fractions were washed with brine, dried over Na_2SO_4 and concentrated. The desired compound was purified with silica column chromatography. ^{1}H NMR (400 MHz, DMSO) δ 7.50 – 7.43 (m, 3H), 7.42 – 7.34 (m, 2H), 6.37

(s, 1H). ^{13}C NMR (100 MHz, DMSO) δ 166.54, 149.41, 132.64, 130.18, 129.15, 127.73, 113.95, 85.91. HRMS (ESI): Exact mass calculated for $C_{10}H_6Br_2O_2$ $[M + H]^+$: 316.8812. Found: 316.8970

4-(benzylamino)-3-chloro-5-phenyl-2,5-dihydrofuran-2-one, Compound 3

General procedure B. ^{1}H NMR (400 MHz, DMSO) δ 7.35 (s, 1H), 7.33 – 7.29 (m, *J* = 7.4 Hz, 5H), 7.18 – 7.05 (m, 5H), 6.57 (s, 1H), 4.32 (d, J = 15.7 Hz, 1H), 4.04 (d, J = 15.6 Hz, 1H). ^{13}C NMR (100 MHz, DMSO) δ 167.11, 154.95, 137.85, 136.03, 128.66, 128.40, 127.77, 127.74, 126.53, 126.12, 121.95, 92.07, 42.65. HRMS (ESI): Exact mass calculated for $C_{17}H_{14}ClNO_2$ $[M + H]^+$: 300.0791. Found: 300.0780

3-chloro-4-[(2-methylpropyl)amino]-5-phenyl-2,5-dihydrofuran-2-one, Compound 4

General procedure B. ^{1}H NMR (400 MHz, DMSO) δ 7.43 – 7.32 (m, 5H), 7.23 (s, 1H), 6.51 (s, 1H), 3.04 (dd, *J* = 13.9, 7.4 Hz, 1H), 2.55 (dd, *J* = 13.9, 7.6 Hz, 1H), 1.65 – 1.50 (m, 1H), 0.69 (dd, *J* = 6.6, 4.0 Hz, 6H). ^{13}C NMR (100 MHz, DMSO) δ 167.31, 154.53, 136.51, 128.73, 128.53, 125.92, 122.03, 91.95, 46.64, 27.08, 20.27, 20.22. HRMS (ESI): Exact mass calculated for $C_{14}H_{16}ClNO_2$ $[M + H]^+$: 266.0947. Found: 266.1802

3,4-dichloro-2,5-dihydrofuran-2-one, Compound 5

Compound 5 was synthesized as described by Bellina *et al.*[1] ^{1}H NMR (400 MHz, $CDCl_3$) δ 4.87 (s, 2H). ^{13}C NMR (100 MHz, $CDCl_3$) δ 165.93, 149.05, 121.26, 71.09. HRMS (ESI): Exact mass calculated for $C_4H_2Cl_2O_2$ $[M + H]^+$: 152.9510. Found: 152.9637

3,4-dichloro-5-methoxy-2,5-dihydrofuran-2-one, Compound 6

Mucochloric acid (250 mg, 1.47 mmol) was dissolved in 15 mL of methanol. A catalytic amount of H_2SO_4 was added and the mixture was allowed to stir overnight. The solution was quenched with a saturated solution of $NaHCO_3$ and extracted 3 × with EtOAc. The organic fractions were combined and washed with brine, dried over Na_2SO_4 and concentrated. The desired compound (119 mg, 45%) was purified by silica column chromatography (10 % EtOAc in hexanes). ^{1}H NMR (400 MHz, $CDCl_3$) δ 5.77 (s, 1H), 3.59 (s, 3H). ^{13}C NMR (100 MHz, $CDCl_3$) δ 163.22,

147.36, 124.73, 101.58, 56.53. HRMS (ESI): Exact mass calculated for $C_5H_4Cl_2O_3$ $[M + H]^+$: 182.9615. Found: 183.2005

3,4-dichloro-5-(prop-2-yn-1-yloxy)-2,5-dihydrofuran-2-one, Compound 7

Mucochloric acid (200 mg, 1.47 mmol) was dissolved in 2 mL of toluene. Propargyl alcohol (119 mg, 1.5 equiv.) and a catalytic amount of H_2SO_4 was added. The flask was equipped with a Dean-stark trap and the solution was refluxed overnight. The solution was quenched with a saturated solution of $NaHCO_3$ and extracted 3 × with EtOAc. The organic fractions were combined and washed with brine, dried over Na_2SO_4 and concentrated. The desired compound (232 mg, 76 %) was purified from the crude mixture by silica column chromatography (10 % EtOAc in hexanes). ^1H NMR (400 MHz, CDCl3) δ 6.05 (s, 1H), 4.50 (dd, *J* = 4.0, 2.4 Hz, 2H), 2.64 (t, *J* = 2.4 Hz, 1H). ^{13}C NMR (100 MHz, $CDCl_3$) δ 163.11, 147.51, 124.61, 98.59, 77.74, 76.75, 57.41. HRMS (ESI): Exact mass calculated for $C_7H_4Cl_2O_3$ $[M + H]^+$: 206.9615. Found: 206.9595

4,5-dichloro-1-phenyl-*1H*-pyrazol-3(2H)-one, Compound 8

Commercial compound used without further purification. HRMS (ESI): Exact mass calculated for $C_9H_6Cl_2N_2O$ $[M + H]^+$: 228.9935. Found: 228.9950

3,4-dichloro-5-hydroxy-2,5-dihydro-*1H*-pyrrol-2-one, Compound 9

Compound 9 (238 mg, 18 %) was synthesized as described by Ratts *et al*.[2] ^1H NMR (400 MHz, DMSO) δ 9.18 (s, 1H), 6.79 (d, *J* = 9.6 Hz, 1H), 5.46 (dd, *J* = 9.6, 1.6 Hz, 1H). ^{13}C NMR (100 MHz, DMSO) δ 163.41, 145.70, 124.94, 79.05. HRMS (ESI): Exact mass calculated for $C_4H_3Cl_2NO_2$ $[M + H]^+$: 167.9619. Found: 167.9630

1-benzyl-3,4-dichloro-5-hydroxy-2,5-dihydro-*1H*-pyrrol-2-one, Compound 10

General procedure A. ^1H NMR (400 MHz, DMSO) δ 7.41 – 7.17 (m, 6H), 5.34 (d, *J* = 9.3 Hz, 1H), 4.72 (d, *J* = 15.6 Hz, 1H), 4.33 (d, *J* = 15.6 Hz, 1H). ^{13}C NMR (100 MHz, DMSO) δ 161.65, 144.47, 136.93, 128.51, 127.69, 127.29, 124.67, 81.46, 43.26.

1-benzyl-3,4-dichloro-*1H*-pyrrol-2(*5H*)-one, Compound 11

Compound 11 (249 mg, 35 %) was synthesized as described by Zhang *et al*.[3] ^{1}H NMR (400 MHz, $CDCl_3$) δ 7.38 – 7.22 (m, 5H), 4.65 (s, 2H), 3.90 (s, 2H). ^{13}C NMR (100 MHz, $CDCl_3$) δ 164.37, 139.96, 136.00, 129.16, 128.37, 128.29, 125.69, 52.93, 47.10. HRMS (ESI): Exact mass calculated for $C_{11}H_9Cl_2NO$ $[M + H]^+$: 242.0139. Found: 242.0674

1-benzyl-3,4-dichloro-5-methoxy-*1H*-pyrrol-2(*5H*)-one, Compound 12

1-benzyl-3,4-dichloro-5-hydroxy-2,5-dihydro-1H-pyrrol-2-one (150 mg, 0.58 mmol) was dissolved in 5 mL of methanol. A catalytic amount of H_2SO_4 was added and the mixture was allowed to stir overnight. The solution was quenched with a saturated solution of $NaHCO_3$ and extracted 3 × with EtOAc. The organic fractions were combined and washed with brine, dried over Na_2SO_4 and concentrated. The desired compound (104 mg, 66 %) was purified from the crude mixture by silica column chromatography (10 to 15 % EtOAc in hexanes).^{1}H NMR (400 MHz, DMSO) δ 7.38 – 7.25 (m, 5H), 5.55 (s, 1H), 4.67 (d, J = 15.4 Hz, 1H), 4.35 (d, J = 15.4 Hz, 1H), 2.98 (s, 3H). ^{13}C NMR (100 MHz, DMSO) δ 162.07, 141.34, 136.42, 128.57, 128.01, 127.53, 126.51, 86.84, 50.49, 44.14. HRMS (ESI): Exact mass calculated for $C_{12}H_{11}Cl_2NO_2$ $[M + H]^+$: 272.0245. Found: 272.0350

1-benzyl-3,4-dichloro-5-oxo-2,5-dihydro-*1H*-pyrrol-2-yl acetate, Compound 13

1-benzyl-3,4-dichloro-5-hydroxy-2,5-dihydro-1H-pyrrol-2-one (150 mg, 0.58 mmol) was dissolved in 3 mL of acetic anhydride. Pyridine (1.16 mol, 2.0 equiv.) was added and the mixture was allowed to stir for 6 hours at room temperature. The reaction was quenched with 10% $HCl_{(aq)}$ and extracted 3 × with EtOAc. The organic fractions were combined and washed with brine, dried over Na_2SO_4 and concentrated. The desired compound (159 mg, 91 %) was purified from the crude mixture by silica column chromatography (5 to 15 % EtOAc in hexanes). ^{1}H NMR (400 MHz, DMSO) δ 7.41 – 7.18 (m, 5H), 6.71 (s, 1H), 4.61 (d, J = 15.7 Hz, 1H), 4.48 (d, J = 15.7 Hz, 1H), 1.88 (s, 3H). ^{13}C NMR (100 MHz, DMSO) δ 169.51, 162.47, 140.89, 136.22, 128.51, 127.68, 127.49, 126.48, 80.42, 44.95, 20.04. HRMS (ESI): Exact mass calculated for $C_{13}H_{11}Cl_2NO_3$ $[M + H]^+$: 300.0194. Found: 300.0236

1-benzyl-3,4-dichloro-*1H*-pyrrole-2,5-dione, Compound 14

Compound 14 was synthesized as described by Holz *et al*.[4] ^{1}H NMR (400 MHz, DMSO) δ 7.44 – 7.17 (m, 5H), 4.67 (s, 2H). ^{13}C NMR (100 MHz, DMSO) δ 163.04, 135.76, 132.56, 128.49, 127.54, 127.42, 42.02. HRMS (ESI): Exact mass calculated for $C_{11}H_7Cl_2NO_2$ $[M + H]^+$: 255.9932. Found: 256.0033

1-benzyl-3,4-dichloro-5-hydroxy-5-phenyl-*1H*-pyrrol-2(*5H*)-one, Compound 15

1-benzyl-3,4-dichloro-1H-pyrrole-2,5-dione (100 mg, 0.39 mmol) was dissolved in 1.95 mL of THF and the solution was cooled to -78 ° C. Phenyllithium (0.39 mmol, 1.0 equivalent, 1.9 M in dibutyl ether) was added dropwise and the solution was allowed to stir for 1 hour before warming to room temperature. The reaction was quenched with a saturated $NH_4Cl_{(aq)}$ solution and extracted 3 × with EtOAc. The organic fractions were combined and washed with brine, dried over Na_2SO_4 and concentrated. The desired compound (64 mg, 49 %) was purified from the crude mixture by silica column chromatography (5 to 15 % EtOAc in hexanes). ^{1}H NMR (400 MHz, DMSO) δ 7.62 (s, 1H), 7.34 (s, 5H), 7.19 – 7.06 (m, 5H), 4.37 (d, *J* = 15.6 Hz, 1H), 4.12 (d, *J* = 15.6 Hz, 1H). ^{13}C NMR (100 MHz, DMSO) δ 162.22, 147.86, 137.09, 135.26, 129.04, 128.61, 127.89, 127.88, 126.81, 126.18, 124.16, 90.83, 43.32. HRMS (ESI): Exact mass calculated for $C_{17}H_{13}Cl_2NO_2$ $[M + H]^+$: 334.0401. Found: 334.0520

1-benzyl-5-hydroxy-*1H*-pyrrol-2(*5H*)-one, Compound 16

Compound 16 (104 mg, 55 %) was synthesized as described by Yamashita *et al*.[5] ^{1}H NMR (400 MHz, DMSO) δ 7.37 – 7.19 (m, 5H), 7.09 (dd, *J* = 6.0, 1.5 Hz, 1H), 6.42 (d, *J* = 8.7 Hz, 1H), 6.18 (d, *J* = 6.0 Hz, 1H), 5.26 (d, *J* = 8.7 Hz, 1H), 4.71 (d, *J* = 15.4 Hz, 1H), 4.20 (d, *J* = 15.4 Hz, 1H). ^{13}C NMR (100 MHz, DMSO) δ 168.80, 147.76, 138.10, 128.43, 127.49, 127.06, 126.99, 81.87, 41.73. HRMS (ESI): Exact mass calculated for $C_{11}H_{11}NO_2$ $[M + H]^+$: 190.0868. Found: 190.0870

1-benzyl-5-hydroxy-3,4-dimethyl-*1H*-pyrrol-2(*5H*)-one, Compound 17

2,3-Dimethylmaleic anhydride (630 mg, 5 mmol) was dissolved in acetic acid (7.5 mL). Benzylamine (535 mg, 5 mmol) was added drop wise and the mixture was refluxed for 2 hours. The solution was concentrated and *1-benzyl-3,4-dimethyl-1H-pyrrole-2,5-dione* (956 mg, 89 %)

was isolated by silica column chromatography (10% EtOAc in hexanes). 1-benzyl-3,4-dimethyl-1H-pyrrole-2,5-dione (215 mg, 1 mmol) was dissolved in THF (3 mL). The solution was cooled to -15 °C and a solution of $LiAlH(O^tBu)_3$ (305 mg, 1.2 equiv.) in THF (2 mL) was added dropwise and the resulting mixture was allowed to react for 1 hour. The solution was warmed to room temperature and stirred for another hour. The reaction was subsequently quenched with 10 % $HCl_{(aq)}$ and extracted 3 × with EtOAc. The organic fractions were combined and washed with brine, dried over Na_2SO_4 and concentrated. The desired compound (147 mg, 68 %) was purified from the crude mixture by silica column chromatography (50 % EtOAc in hexanes). ^{1}H NMR (400 MHz, DMSO) δ 7.38 – 7.17 (m, 5H), 6.25 (d, *J* = 8.7 Hz, 1H), 4.92 (d, *J* = 8.6 Hz, 1H), 4.71 (d, *J* = 15.4 Hz, 1H), 4.19 (d, *J* = 15.4 Hz, 1H), 1.86 (s, *J* = 0.5 Hz, 3H), 1.70 (s, *J* = 1.1 Hz, 3H). ^{13}C NMR (100 MHz, DMSO) δ 169.94, 149.58, 138.33, 128.39, 127.56, 127.27, 126.91, 82.19, 42.04, 11.18, 8.25. HRMS (ESI): Exact mass calculated for $C_{13}H_{15}NO_2$ $[M + H]^+$: 218.1181. Found: 218.1077

3,4-dichloro-1-(furan-2-ylmethyl)-5-hydroxy-*1H*-pyrrol-2(*5H*)-one, Compound 18

General procedure A. ^{1}H NMR (400 MHz, DMSO) δ 7.59 (d, *J* = 1.1 Hz, 1H), 7.21 (d, *J* = 9.3 Hz, 1H), 6.40 (dd, *J* = 3.1, 1.9 Hz, 1H), 6.36 (d, *J* = 3.1 Hz, 1H), 5.34 (d, *J* = 8.9 Hz, 1H), 4.71 (d, *J* = 16.0 Hz, 1H), 4.32 (d, *J* = 16.0 Hz, 1H). ^{13}C NMR (100 MHz, DMSO) δ 161.29, 149.86, 144.73, 142.77, 124.56, 110.55, 108.22, 81.37, 36.17. HRMS (ESI): Exact mass calculated for $C_9H_7Cl_2NO_3$ $[M + H]^+$: 247.9881. Found: 247.9958

3,4-dichloro-5-hydroxy-1-(thiophen-2-ylmethyl)-*1H*-pyrrol-2(*5H*)-one, Compound 19

General procedure A. ^{1}H NMR (400 MHz, DMSO) δ 7.45 (dd, *J* = 5.1, 1.1 Hz, 1H), 7.28 (d, *J* = 9.3 Hz, 1H), 7.05 (d, *J* = 2.8 Hz, 1H), 6.98 (dd, *J* = 5.0, 3.5 Hz, 1H), 5.36 (d, *J* = 9.3 Hz, 1H), 4.84 (d, *J* = 15.8 Hz, 1H), 4.52 (d, *J* = 15.8 Hz, 1H). ^{13}C NMR (101 MHz, DMSO) δ 155.26, 138.69, 132.99, 120.95, 120.94, 120.05, 118.54, 75.12, 31.88. HRMS (ESI): Exact mass calculated for $C_9H_7Cl_2NO_2S$ $[M + H]^+$: 263.9652. Found: 263.9713

2-benzyl-3-hydroxyisoindolin-1-one, Compound 20

Phthalic anhydride (1.48 g, 10 mmol) was dissolved in acetic acid (15 mL). Benzylamine (1.70 g, 10 mmol) was added dropwise and the mixture was refluxed for 2 hours. The solution was

concentrated and *2-benzylisoindoline-1,3-dione* (2.07 g, 96 %) was isolated by silica column chromatography (10% EtOAc in hexanes). 2-benzylisoindoline-1,3-dione (237 mg, 1 mmol) was dissolved in THF (3 mL). The solution was cooled to -15 °C and a solution of $LiAlH(O^tBu)_3$ (305 mg, 1.2 equiv.) in THF (2 mL) was added dropwise and the resulting mixture was allowed to react for 1 hour. The solution was warmed to room temperature and stirred for another hour. The reaction was subsequently quenched with 10 % $HCl_{(aq)}$ and extracted 3 × with EtOAc. The organic fractions were combined and washed with brine, dried over Na_2SO_4 and concentrated. The desired compound (161 mg, 67 %) was purified from the crude mixture by silica column chromatography (40 % EtOAc in hexanes)^{1}H NMR (400 MHz, DMSO) δ 7.75 – 7.50 (m, 4H), 7.39 – 7.21 (m, 5H), 6.75 (d, *J* = 8.8 Hz, 1H), 5.67 (d, *J* = 8.8 Hz, 1H), 4.92 (d, *J* = 15.3 Hz, 1H), 4.37 (d, *J* = 15.3 Hz, 1H). ^{13}C NMR (100 MHz, DMSO) δ 166.14, 144.88, 137.73, 132.07, 131.41, 129.40, 128.48, 127.67, 127.09, 123.74, 122.45, 80.28, 42.11. HRMS (ESI): Exact mass calculated for $C_{15}H_{13}NO_2$ $[M + H]^+$: 240.1024. Found: 240.1022

_N_-(2-(1-benzyl-4-chloro-2-hydroxy-5-oxo-2,5-dihydro-_1H_-pyrrol-3-ylthio)ethyl)acetamide, Compound 21

1-benzyl-3,4-dichloro-5-hydroxy-2,5-dihydro-1H-pyrrol-2-one (100 mg, 0.39 mmol) and N-acetylcysteamine (52 mg, 0.43 mmol) were added to 1 ml of DMSO. The mixture was allowed to stir overnight at room temperature. The desired compound (73 mg, 55 %) was purified from the mixture by silica column chromatography (70 % EtOAc in hexanes). ^{1}H NMR (400 MHz, DMSO) δ 8.11 (s, 1H), 7.38 – 7.20 (m, 5H), 7.04 (d, *J* = 9.9 Hz, 1H), 5.48 (d, *J* = 9.9 Hz, 1H), 4.69 (d, *J* = 15.6 Hz, 1H), 4.29 (d, *J* = 15.6 Hz, 1H), 3.32 – 3.14 (m, 4H), 1.75 (s, 3H). ^{13}C NMR (100 MHz, DMSO) δ 169.46, 162.55, 151.04, 137.36, 128.48, 127.62, 127.18, 119.59, 81.14, 43.07, 38.77, 29.61, 22.42. HRMS (ESI): Exact mass calculated for $C_{15}H_{17}ClN_2O_3S$ $[M + H]^+$: 341.0726. Found: 341.0738

3,4-dichloro-5-hydroxy-1-methyl-_1H_-pyrrol-2(_5H_)-one, Compound 22

General procedure A. ^{1}H NMR (400 MHz, MeOD) δ 5.28 (s, 1H), 2.97 (s, 3H). ^{13}C NMR (100 MHz, MeOD) δ 164.28, 145.42, 126.72, 84.67, 26.90. HRMS (ESI): Exact mass calculated for $C_5H_5Cl_2NO_2$ $[M + H]^+$: 181.9775. Found: 181.9820

3,4-dichloro-1-ethyl-5-hydroxy-1H-pyrrol-2(*5H*)-one, Compound 23

General procedure A. ^{1}H NMR (400 MHz, $CDCl_3$) δ 5.29 (s, 1H), 4.67 (s, 1H), 3.61 (dq, *J* = 14.5, 7.3 Hz, 1H), 3.35 (dq, *J* = 14.2, 7.1 Hz, 1H), 1.18 (t, *J* = 7.2 Hz, 3H). ^{13}C NMR (100 MHz, $CDCl_3$) δ 162.97, 143.68, 126.38, 81.98, 35.43, 13.63. HRMS (ESI): Exact Mass for $[M + H]^+$: Not determined

3,4-dichloro-5-hydroxy-1-propyl-*1H*-pyrrol-2(*5H*)-one, Compound 24

General procedure A. ^{1}H NMR (400 MHz, $CDCl_3$) δ 5.26 (s, 1H), 3.89 (s, 1H), 3.52 (ddd, *J* = 14.0, 8.5, 7.2 Hz, 1H), 3.28 (ddd, *J* = 14.0, 8.5, 5.6 Hz, 1H), 1.72 – 1.51 (m, 2H), 0.92 (t, *J* = 7.4 Hz, 3H). ^{13}C NMR (100 MHz, $CDCl_3$) δ 163.06, 143.44, 126.71, 82.35, 42.18, 21.71, 11.42. HRMS (ESI): Exact mass calculated for $C_7H_9Cl_2NO_2$ $[M + H]^+$: 210.0088. Found: 210.0150

3,4-dichloro-1-cyclopropyl-5-hydroxy-*1H*-pyrrol-2(*5H*)-one, Compound 25

General procedure A. ^{1}H NMR (400 MHz, MeOD) δ 5.32 (s, 1H), 2.65 – 2.58 (m, 1H), 1.04 – 0.96 (m, 1H), 0.89 – 0.71 (m, 4H). ^{13}C NMR (100 MHz, MeOD) δ 165.39, 145.51, 126.40, 84.48, 24.26, 5.85, 5.34. HRMS (ESI): Exact mass calculated for $C_7H_7Cl_2NO_2$ $[M + H]^+$: 207.9932. Found: 207.9984

1-allyl-3,4-dichloro-5-hydroxy-*1H*-pyrrol-2(*5H*)-one, Compound 26

General procedure A. ^{1}H NMR (400 MHz, DMSO) δ 7.07 (d, *J* = 9.3 Hz, 1H), 5.79 (dddd, *J* = 16.6, 10.3, 6.3, 4.9 Hz, 1H), 5.39 (d, *J* = 9.1 Hz, 1H), 5.16 (ddq, *J* = 14.6, 10.2, 1.5 Hz, 2H), 4.11 (ddt, *J* = 16.1, 4.7, 1.6 Hz, 1H), 3.78 (ddt, *J* = 16.1, 6.3, 1.3 Hz, 1H). ^{13}C NMR (100 MHz, DMSO) δ 161.29, 144.36, 133.01, 124.65, 117.21, 81.34, 42.01. HRMS (ESI): Exact mass calculated for $C_7H_7Cl_2NO_2$ $[M + H]^+$: 207.9932. Found: 207.9976

3,4-dichloro-5-hydroxy-1-(prop-2-ynyl)-*1H*-pyrrol-2(*5H*)-one, Compound 27

General procedure A. ^{1}H NMR (400 MHz, DMSO) δ 7.24 (d, *J* = 9.3 Hz, 1H), 5.47 (d, *J* = 9.0 Hz, 1H), 4.33 (dd, *J* = 17.9, 2.5 Hz, 1H), 3.95 (dd, *J* = 17.9, 2.5 Hz, 1H), 3.27 (t, *J* = 2.5 Hz, 1H). ^{13}C NMR (100 MHz, DMSO) δ 161.10, 145.01, 124.42, 81.19, 78.48, 74.51, 29.11. HRMS (ESI): Exact mass calculated for $C_7H_5Cl_2NO_2$ $[M + H]^+$: 205.9775. Found: 205.9814

3,4-dichloro-5-hydroxy-1-(2-morpholinoethyl)-*1H*-pyrrol-2(*5H*)-one, Compound 28

General procedure A. ^{1}H NMR (400 MHz, $CDCl_3$) δ 9.10 (s, 1H), 5.22 (s, 1H), 4.14 (dt, *J* = 15.3, 3.0 Hz, 1H), 3.77 (t, *J* = 4.6 Hz, 4H), 3.22 (ddd, *J* = 15.3, 11.6, 2.1 Hz, 1H), 2.80 – 2.71 (m, 2H), 2.67 (ddd, *J* = 14.2, 11.6, 2.7 Hz, 1H), 2.53 – 2.44 (m, 3H). ^{13}C NMR (100 MHz, $CDCl_3$) δ 163.02, 144.37, 125.84, 82.91, 66.27, 58.20, 53.36, 39.37. HRMS (ESI): Exact mass calculated for $C_{10}H_{14}Cl_2N_2O_3$ $[M + H]^+$: 281.0459. Found: 281.0402

3,4-dichloro-5-hydroxy-1-phenethyl-*1H*-pyrrol-2(*5H*)-one, Compound 29

General procedure A. ^{1}H NMR (400 MHz, MeOD) δ 7.38 – 7.17 (m, 5H), 5.13 (s, 1H), 3.85 – 3.73 (m, 1H), 3.61 – 3.44 (m, 1H), 3.04 – 2.81 (m, 2H). ^{13}C NMR (100 MHz, MeOD) δ 164.13, 145.56, 139.92, 129.78, 129.67, 127.64, 83.66, 42.95, 35.43. HRMS (ESI): Exact mass calculated for $C_{12}H_{11}Cl_2NO_2$ $[M + H]^+$: 272.0245. Found: 272.0350

3,4-dichloro-5-hydroxy-1-(3-phenylpropyl)-*1H*-pyrrol-2(*5H*)-one, Compound 30

General procedure A. ^{1}H NMR (400 MHz, DMSO) δ 7.21 (qdd, *J* = 8.5, 5.1, 1.5 Hz, 5H), 7.03 (d, *J* = 9.6 Hz, 1H), 5.45 (d, *J* = 9.5 Hz, 1H), 3.46 (dt, *J* = 14.9, 7.5 Hz, 1H), 3.25 (ddd, *J* = 13.8, 7.9, 5.8 Hz, 1H), 2.58 (t, *J* = 7.8 Hz, 2H), 1.93 – 1.74 (m, 2H). ^{13}C NMR (100 MHz, DMSO) δ 161.49, 144.02, 141.43, 128.27, 128.25, 125.77, 124.67, 81.55, 39.65, 32.47, 29.53. HRMS (ESI): Exact mass calculated for $C_{13}H_{13}Cl_2NO_2$ $[M + H]^+$: 286.0401. Found: 286.0529

3,4-dichloro-5-hydroxy-1-(4-phenylbutyl)-*1H*-pyrrol-2(*5H*)-one, Compound 31

General procedure A. ^{1}H NMR (400 MHz, DMSO) δ 7.32 – 7.23 (m, 2H), 7.22 – 7.13 (m, 3H), 7.03 (d, *J* = 9.4 Hz, 1H), 5.41 (d, *J* = 9.3 Hz, 1H), 3.44 (dd, *J* = 13.8, 7.2 Hz, 1H), 3.30 – 3.19 (m, 1H), 2.64 – 2.53 (m, 2H), 1.63 – 1.48 (m, 4H). ^{13}C NMR (100 MHz, DMSO) δ 161.45, 143.98, 142.01, 128.28, 128.22, 125.66, 124.67, 81.50, 39.60, 34.70, 28.25, 27.44. HRMS (ESI): Exact mass calculated for $C_{14}H_{15}Cl_2NO_2$ $[M + H]^+$: 300.0558. Found: 300.0699

1-(biphenyl-4-ylmethyl)-3,4-dichloro-5-hydroxy-*1H*-pyrrol-2(*5H*)-one, Compound 32

General procedure A. ^{1}H NMR (400 MHz, DMSO) δ 7.68 – 7.60 (m, 4H), 7.50 – 7.32 (m, 5H), 7.27 (d, *J* = 9.2 Hz, 1H), 5.40 (d, *J* = 9.2 Hz, 1H), 4.75 (d, *J* = 15.6 Hz, 1H), 4.39 (d, *J* = 15.6 Hz, 1H). ^{13}C NMR (100 MHz, DMSO) δ 161.68, 144.50, 139.82, 139.24, 136.18, 128.92, 128.36,

127.42, 126.82, 126.61, 124.68, 81.53, 43.03. HRMS (ESI): Exact Mass for $[M + H]^+$: Not determined

3,4-dichloro-5-hydroxy-1-(3-phenylprop-2-ynyl)-*1H*-pyrrol-2(*5H*)-one, Compound 33

General procedure A. ^{1}H NMR (400 MHz, DMSO) δ 7.45 (dt, *J* = 8.6, 3.7 Hz, 2H), 7.41 – 7.35 (m, 3H), 7.28 (d, *J* = 9.3 Hz, 1H), 5.60 (d, *J* = 9.0 Hz, 1H), 4.60 (d, *J* = 18.0 Hz, 1H), 4.22 (d, *J* = 18.0 Hz, 1H). ^{13}C NMR (100 MHz, DMSO) δ 161.12, 145.11, 131.53, 128.78, 128.61, 124.43, 121.83, 84.26, 82.88, 81.24, 29.81. HRMS (ESI): Exact mass calculated for $C_{13}H_9Cl_2NO_2$ $[M + H]^+$: 282.0088. Found: 282.0056

3,4-dichloro-5-hydroxy-1-(1-phenylethyl)-*1H*-pyrrol-2(*5H*)-one, Compound 34

General procedure A. Compound isolated as a 1:1 mixture of diastereoisomers. ^{1}H NMR (400 MHz, DMSO) δ 7.44 – 7.22 (m, 5H+5H), 7.19 (d, *J* = 9.4 Hz, 1H), 7.11 (d, *J* = 9.8 Hz, 1H), 5.62 (d, *J* = 9.4 Hz, 1H), 5.29 (d, *J* = 9.7 Hz, 1H), 5.12 (q, *J* = 7.2 Hz, 1H), 4.97 (q, *J* = 7.3 Hz, 1H), 1.68 (d, *J* = 7.4 Hz, 3H), 1.65 (d, *J* = 7.3 Hz, 3H). ^{13}C NMR (100 MHz, DMSO) δ 161.46, 161.21, 144.22, 144.14, 142.59, 140.49, 128.38, 128.13, 127.32, 127.09, 126.86, 126.69, 124.46, 124.41, 81.49, 81.31, 52.00, 51.30, 18.72, 18.14. HRMS (ESI): Exact mass calculated for $C_{12}H_{11}Cl_2NO_2$ $[M + H]^+$: 272.0245. Found: 272.0298

1-benzhydryl-3,4-dichloro-5-hydroxy-*1H*-pyrrol-2(*5H*)-one, Compound 35

General procedure A. ^{1}H NMR (400 MHz, DMSO) δ 7.43 – 7.19 (m, 11H), 6.12 (s, 1H), 5.26 (s, 1H). ^{13}C NMR (100 MHz, DMSO) δ 161.42, 144.45, 139.63, 138.62, 129.12, 128.18, 128.10, 127.49, 127.21, 124.40, 81.91, 60.56. HRMS (ESI): Exact mass calculated for $C_{17}H_{13}Cl_2NO_2$ $[M + H]^+$: 334.0401. Found: 334.0435

3,4-dichloro-5-hydroxy-1-(2-methoxybenzyl)-*1H*-pyrrol-2(*5H*)-one, Compound 36

General procedure A. ^{1}H NMR (400 MHz, DMSO) δ 7.26 (td, *J* = 8.2, 1.6 Hz, 1H), 7.14 (dd, *J* = 7.5, 1.4 Hz, 1H), 6.99 (d, *J* = 7.8 Hz, 1H), 6.90 (td, *J* = 7.4, 0.8 Hz, 1H), 5.35 (s, 1H), 4.58 (d, *J* = 16.1 Hz, 1H), 4.40 (d, *J* = 16.1 Hz, 1H), 3.80 (s, 3H). ^{13}C NMR (100 MHz, DMSO) δ 161.71, 156.65, 144.48, 128.59, 128.30, 124.68, 124.38, 120.21, 110.64, 81.75, 55.38, 38.51. HRMS (ESI): Exact mass calculated for $C_{12}H_{11}Cl_2NO_3$ $[M + Na]^+$: 310.0276. Found: 310.0014

3,4-dichloro-5-hydroxy-1-(2-hydroxybenzyl)-*1H*-pyrrol-2(*5H*)-one, Compound 37

General procedure A. ^{1}H NMR (400 MHz, DMSO) δ 9.61 (s, 1H), 7.15 – 7.03 (m, 3H), 6.81 (dd, *J* = 7.9, 0.7 Hz, 1H), 6.74 (td, *J* = 7.5, 1.0 Hz, 1H), 5.34 (d, *J* = 8.8 Hz, 1H), 4.57 (d, *J* = 15.9 Hz, 1H), 4.36 (d, *J* = 15.9 Hz, 1H). ^{13}C NMR (100 MHz, DMSO) δ 161.68, 154.85, 144.38, 128.70, 128.29, 124.70, 122.78, 118.88, 114.96, 81.67, 38.66. HRMS (ESI): Exact mass calculated for $C_{11}H_9Cl_2NO_3$ $[M + H]^+$: 274.0037. Found: 274.0120

3,4-dichloro-1-(2-chlorobenzyl)-5-hydroxy-*1H*-pyrrol-2(*5H*)-one, Compound 38

General procedure A. ^{1}H NMR (400 MHz, DMSO) δ 7.51 – 7.42 (m, 1H), 7.37 – 7.26 (m, 3H), 7.21 (d, *J* = 9.3 Hz, 1H), 5.41 (d, *J* = 9.3 Hz, 1H), 4.70 (d, *J* = 16.4 Hz, 1H), 4.52 (d, *J* = 16.4 Hz, 1H). ^{13}C NMR (100 MHz, DMSO) δ 161.88, 144.77, 133.98, 131.85, 129.27, 129.19, 129.07, 127.33, 124.60, 81.93, 41.29. HRMS (ESI): Exact mass calculated for $C_{11}H_8Cl_3NO_2$ $[M + H]^+$: 291.9698. Found: 291.9922

3,4-dichloro-5-hydroxy-1-(4-(methylsulfonyl)benzyl)-*1H*-pyrrol-2(*5H*)-one, Compound 39

General procedure A. ^{1}H NMR (400 MHz, DMSO) δ 7.89 (d, *J* = 8.3 Hz, 2H), 7.56 (d, *J* = 8.3 Hz, 2H), 7.25 (d, *J* = 9.3 Hz, 1H), 5.44 (d, *J* = 9.2 Hz, 1H), 4.74 (d, *J* = 16.2 Hz, 1H), 4.52 (d, *J* = 16.3 Hz, 1H), 3.20 (s, 3H). ^{13}C NMR (100 MHz, DMSO) δ 161.87, 144.69, 143.09, 139.72, 128.36, 127.15, 124.59, 81.92, 43.52, 43.17. HRMS (ESI): Exact mass calculated for $C_{12}H_{11}Cl_2NO_4S$ $[M + H]^+$: 335.9864. Found: 335.9834

3,4-dichloro-5-hydroxy-1-(4-(trifluoromethyl)benzyl)-*1H*-pyrrol-2(*5H*)-one, Compound 40

General procedure A. ^{1}H NMR (400 MHz, DMSO) δ 7.70 (d, *J* = 8.2 Hz, 2H), 7.52 (d, *J* = 8.1 Hz, 2H), 7.24 (d, *J* = 9.3 Hz, 1H), 5.43 (d, *J* = 9.3 Hz, 1H), 4.74 (d, *J* = 16.1 Hz, 1H), 4.49 (d, *J* = 16.1 Hz, 1H). ^{13}C NMR (100 MHz, DMSO) δ 161.85, 144.67, 141.91 (d, $J_{(C,F)}$ = 1.1 Hz), 128.38, 127.40 (q, $J_{(C,F)}$ = 31.7 Hz), 125.32 (q, $J_{(C,F)}$ = 3.8 Hz), 124.60, 124.27 (q, $J_{(C,F)}$ = 272.0 Hz), 81.84, 43.13. HRMS (ESI): Exact mass calculated for $C_{12}H_8Cl_2F_3NO_2$ $[M + H]^+$: 325.9962. Found: 325.9960

3,4-dichloro-5-hydroxy-1-(4-methylbenzyl)-*1H*-pyrrol-2(*5H*)-one, Compound 41

General procedure A. ^{1}H NMR (400 MHz, DMSO) δ 7.23 – 7.11 (m, 5H), 5.30 (d, *J* = 9.3 Hz, 1H), 4.68 (d, *J* = 15.4 Hz, 1H), 4.26 (d, *J* = 15.4 Hz, 1H), 2.27 (s, 3H). ^{13}C NMR (100 MHz, DMSO) δ 161.54, 144.40, 136.46, 133.86, 129.05, 127.73, 124.66, 81.30, 42.95, 20.66. HRMS (ESI): Exact mass calculated for $C_{12}H_{11}Cl_2NO_2$ $[M + H]^+$: 272.0245. Found: 272.0455

3,4-dichloro-5-hydroxy-1-(4-methoxybenzyl)-*1H*-pyrrol-2(*5H*)-one, Compound 42

General procedure A. ^{1}H NMR (400 MHz, DMSO) δ 7.30 – 7.10 (m, 3H), 6.90 (t, *J* = 5.7 Hz, 2H), 5.29 (d, *J* = 9.3 Hz, 1H), 4.66 (d, *J* = 15.2 Hz, 1H), 4.23 (d, *J* = 15.2 Hz, 1H), 3.73 (s, 3H). ^{13}C NMR (100 MHz, DMSO) δ 155.48, 152.58, 138.37, 123.23, 122.82, 118.65, 107.91, 75.21, 49.08, 36.67. HRMS (ESI): Exact mass calculated for $C_{12}H_{11}Cl_2NO_3$ $[M + H]^+$: 288.0194. Found: 288.0409

3,4-dichloro-5-hydroxy-1-(4-hydroxybenzyl)-*1H*-pyrrol-2(*5H*)-one, Compound 43

General procedure A. ^{1}H NMR (400 MHz, DMSO) δ 9.37 (s, 1H), 7.18 (d, *J* = 9.1 Hz, 1H), 7.08 (d, *J* = 8.5 Hz, 2H), 6.71 (d, *J* = 8.5 Hz, 2H), 5.26 (d, *J* = 8.3 Hz, 1H), 4.63 (d, *J* = 15.1 Hz, 1H), 4.15 (d, *J* = 15.2 Hz, 1H). ^{13}C NMR (100 MHz, DMSO) δ 161.41, 156.71, 144.33, 129.24, 127.00, 124.66, 115.25, 81.07, 42.68. HRMS (ESI): Exact mass calculated for $C_{11}H_9Cl_2NO_3$ $[M + H]^+$: 274.0037. Found: 274.0144

3,4-dichloro-1-(2-fluorobenzyl)-5-hydroxy-*1H*-pyrrol-2(*5H*)-one, Compound 44

General procedure A. ^{1}H NMR (400 MHz, DMSO) δ 7.43 – 7.27 (m, 2H), 7.25 – 7.11 (m, 3H), 5.36 (d, *J* = 9.4 Hz, 1H), 4.70 (d, *J* = 15.8 Hz, 1H), 4.46 (d, *J* = 15.8 Hz, 1H). ^{13}C NMR (100 MHz, DMSO) δ 161.66, 160.04 (d, $J_{(C,F)}$ = 245.1 Hz), 144.68, 130.13 (d, $J_{(C,F)}$ = 4.0 Hz), 129.51 (d, $J_{(C,F)}$ = 8.1 Hz), 124.55, 124.47 (d, $J_{(C,F)}$ = 3.5 Hz), 123.63 (d, $J_{(C,F)}$ = 14.7 Hz), 115.26 (d, $J_{(C,F)}$ = 21.1 Hz), 81.71, 37.18, 37.16 (d, $J_{(C,F)}$ = 4.5 Hz). HRMS (ESI): Exact mass calculated for $C_{11}H_8Cl_2FNO_2$ $[M + H]^+$: 275.9994. Found: 276.0044

3,4-dichloro-1-(3-fluorobenzyl)-5-hydroxy-*1H*-pyrrol-2(*5H*)-one, Compound 45

General procedure A. ^{1}H NMR (400 MHz, DMSO) δ 7.43 – 7.33 (m, 1H), 7.23 (d, *J* = 9.3 Hz, 1H), 7.11 (ddd, *J* = 16.9, 8.8, 2.7 Hz, 3H), 5.41 (d, *J* = 9.2 Hz, 1H), 4.68 (d, *J* = 15.9 Hz, 1H), 4.40 (d, *J* = 15.9 Hz, 1H). ^{13}C NMR (100 MHz, DMSO) δ 162.24 (d, $J_{(C,F)}$ = 243.6 Hz), 161.79,

144.60, 139.98 (d, $J_{(C,F)}$ = 7.2 Hz), 130.42 (d, $J_{(C,F)}$ = 8.3 Hz), 124.62, 123.62 (d, $J_{(C,F)}$ = 2.7 Hz), 114.36 (d, $J_{(C,F)}$ = 21.8 Hz), 114.05 (d, $J_{(C,F)}$ = 20.9 Hz), 81.73, 42.94. HRMS (ESI): Exact mass calculated for $C_{11}H_8Cl_2FNO_2$ $[M + H]^+$: 275.9994. Found: 275.9965

3,4-dichloro-1-(4-fluorobenzyl)-5-hydroxy-*1H*-pyrrol-2(*5H*)-one, Compound 46

General procedure A. ^{1}H NMR (400 MHz, DMSO) δ 7.38 – 7.29 (m, 2H), 7.22 (d, *J* = 9.3 Hz, 1H), 7.19 – 7.12 (m, 2H), 5.35 (d, *J* = 9.2 Hz, 1H), 4.67 (d, *J* = 15.5 Hz, 1H), 4.34 (d, *J* = 15.6 Hz, 1H). ^{13}C NMR (100 MHz, DMSO) δ 162.65, 161.46 (d, $J_{(C,F)}$ = 243.0 Hz), 144.49, 133.18 (d, $J_{(C,F)}$ = 3.0 Hz), 129.83 (d, $J_{(C,F)}$ = 8.2 Hz), 124.64, 115.22 (d, $J_{(C,F)}$ = 23.4 Hz), 81.51, 42.66. HRMS (ESI): Exact mass calculated for $C_{11}H_8Cl_2FNO_2$ $[M + H]^+$: 275.9994. Found: 276.0105

3,4-dichloro-5-hydroxy-1-(3,4,5-trifluorobenzyl)-*1H*-pyrrol-2(*5H*)-one, Compound 47

General procedure A. ^{1}H NMR (400 MHz, DMSO) δ 7.31 – 7.23 (m, 2H), 7.19 (d, *J* = 9.5 Hz, 1H), 5.47 – 5.42 (m, 1H), 4.62 (d, *J* = 16.2 Hz, 1H), 4.41 (d, *J* = 16.2 Hz, 1H). ^{13}C NMR (100 MHz, DMSO) δ 161.94, 151.37 (m), 148.91 (m), 144.72, 134.60 (m), 124.56, 112.09 (dd, $J_{(C,F)}$ = 15.8 Hz, 5.4 Hz), 81.89, 42.47. HRMS (ESI): Exact mass calculated for $C_{11}H_6Cl_2F_3NO_2$ $[M + H]^+$: 311.9805. Found: 311.9908

3,4-dichloro-5-hydroxy-1-(naphthalen-1-ylmethyl)-*1H*-pyrrol-2(*5H*)-one, Compound 48

General procedure A. ^{1}H NMR (400 MHz, DMSO) δ 8.17 (d, *J* = 8.2 Hz, 1H), 7.95 (dd, *J* = 9.6, 8.4 Hz, 1H), 7.89 (d, *J* = 7.7 Hz, 1H), 7.64 – 7.40 (m, 4H), 7.25 (d, *J* = 9.4 Hz, 1H), 5.27 (d, *J* = 10.6 Hz, 1H), 5.23 (d, *J* = 4.1 Hz, 1H), 4.75 (d, *J* = 15.7 Hz, 1H). ^{13}C NMR (100 MHz, DMSO) δ 161.51, 144.71, 133.35, 131.90, 130.71, 128.61, 128.15, 126.53, 126.31, 125.96, 125.45, 124.55, 123.14, 81.39, 40.88. HRMS (ESI): Exact mass calculated for $C_{15}H_{11}Cl_2NO_2$ $[M + H]^+$: 308.0245. Found: 308.0365

3,4-dichloro-5-hydroxy-1-(pyridin-2-ylmethyl)-*1H*-pyrrol-2(*5H*)-one, Compound 49

General procedure A. ^{1}H NMR (400 MHz, $CDCl_3$) δ 8.46 (d, *J* = 5.0 Hz, 1H), 7.80 (td, *J* = 7.7, 1.7 Hz, 1H), 7.47 (d, *J* = 7.8 Hz, 1H), 7.32 (dd, *J* = 7.4, 5.1 Hz, 1H), 5.50 (s, 1H), 5.04 (d, *J* = 16.0 Hz, 1H), 4.64 (d, *J* = 16.0 Hz, 1H). ^{13}C NMR (100 MHz, $CDCl_3$) δ 163.17, 155.25, 147.93,

145.39, 138.88, 125.81, 123.57, 123.47, 83.45, 46.46. HRMS (ESI): Exact mass calculated for $C_{10}H_8Cl_2N_2O_2$ [M + H]$^+$: 259.0041. Found: 259.0025

3,4-dichloro-5-hydroxy-1-(pyridin-3-ylmethyl)-*1H*-pyrrol-2(*5H*)-one, Compound 50
General procedure A. ^{1}H NMR (400 MHz, DMSO) δ 8.54 (s, 1H), 8.48 (d, *J* = 3.9 Hz, 1H), 7.71 (d, *J* = 7.9 Hz, 1H), 7.36 (dd, *J* = 7.8, 4.8 Hz, 1H), 7.25 (d, *J* = 9.2 Hz, 1H), 5.43 (d, *J* = 8.8 Hz, 1H), 4.67 (d, *J* = 15.8 Hz, 1H), 4.44 (d, *J* = 15.8 Hz, 1H). ^{13}C NMR (100 MHz, DMSO) δ 161.80, 149.03, 148.46, 144.61, 135.68, 132.71, 124.58, 123.57, 81.84, 41.28. HRMS (ESI): Exact mass calculated for $C_{10}H_8Cl_2N_2O_2$ [M + H]$^+$: 259.0041. Found: 259.0009

3,4-dichloro-5-hydroxy-1-(pyridin-4-ylmethyl)-*1H*-pyrrol-2(*5H*)-one, Compound 51
General procedure A. ^{1}H NMR (400 MHz, DMSO) δ 8.53 (d, *J* = 6.0 Hz, 2H), 7.34 (d, *J* = 5.7 Hz, 2H), 7.29 (s, 1H), 5.47 (s, 1H), 4.67 (d, *J* = 16.7 Hz, 1H), 4.48 (d, *J* = 16.7 Hz, 1H). ^{13}C NMR (100 MHz, DMSO) δ 161.99, 149.17, 146.80, 144.77, 124.60, 122.58, 82.05, 42.67. HRMS (ESI): Exact mass calculated for $C_{10}H_8Cl_2N_2O_2$ [M + H]$^+$: 259.0041. Found: 259.0214

Spectra and titers:
^{1}H NMR, ^{13}C NMR, MRM transitions, VEU titer

Compound ID: 1

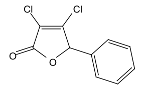

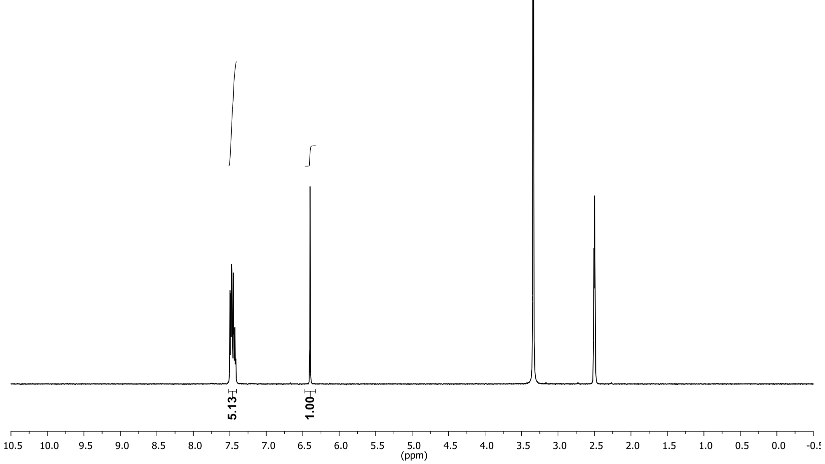

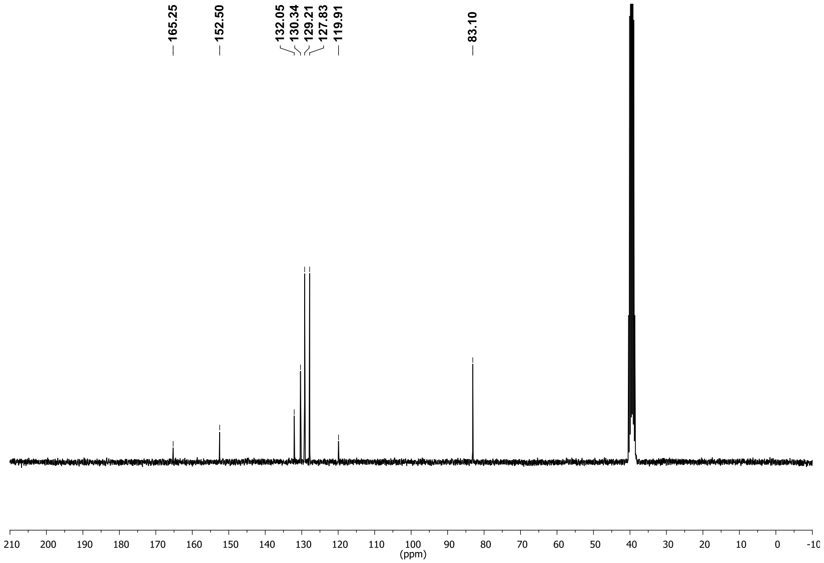

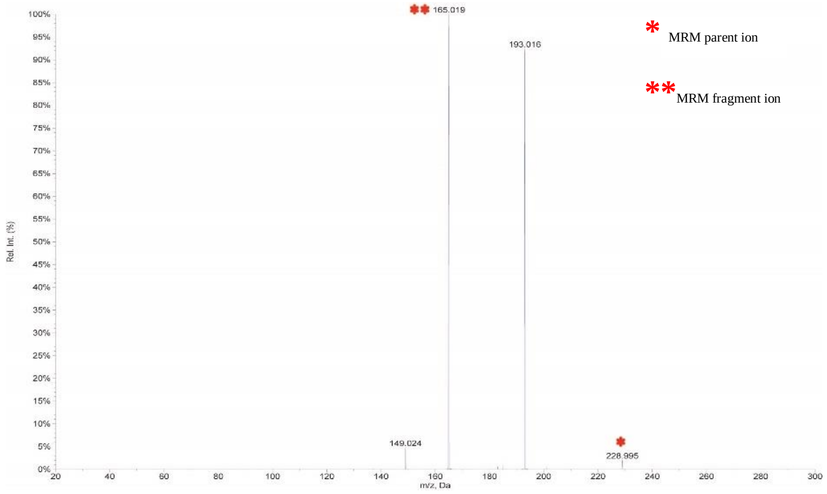

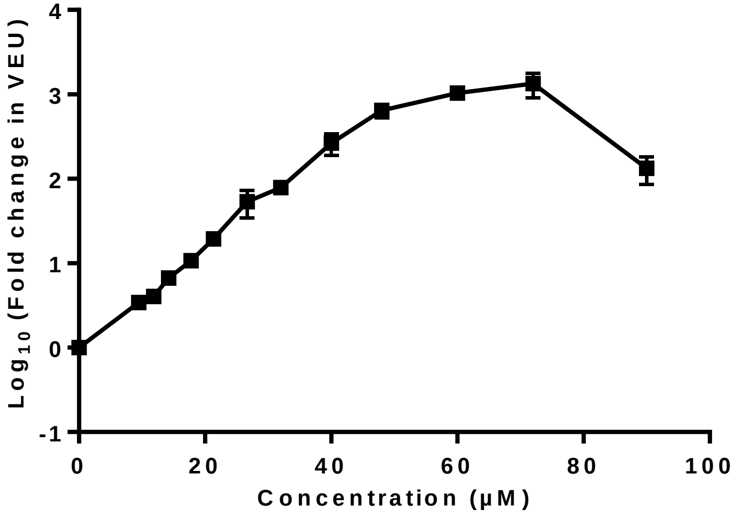

Compound ID: 2

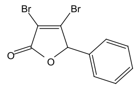

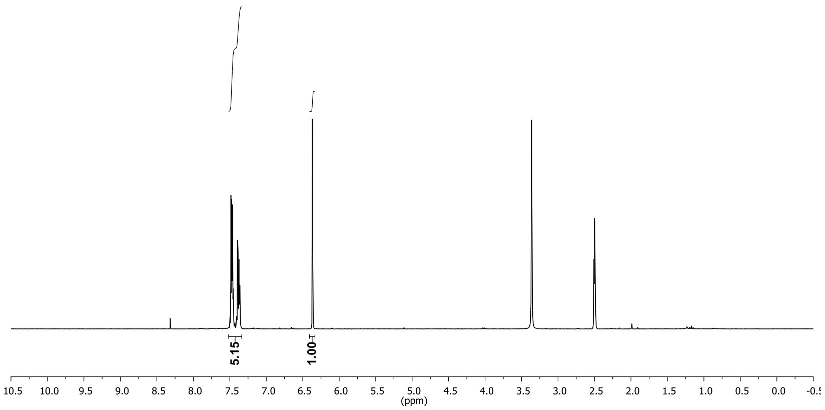

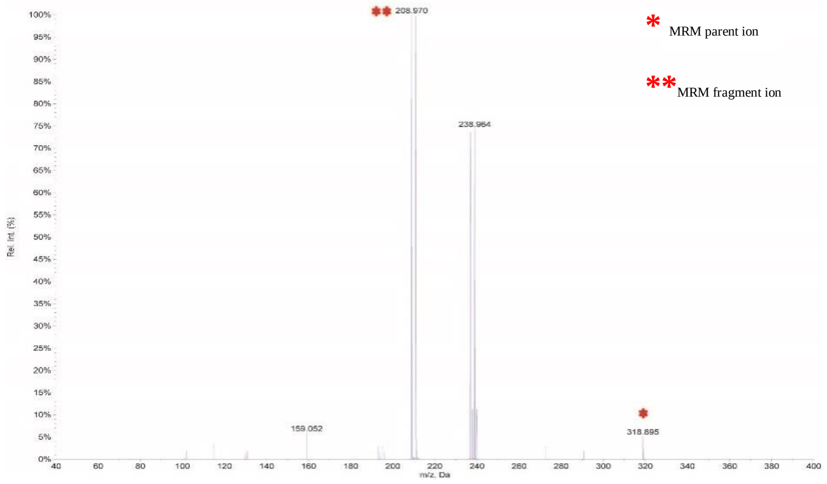

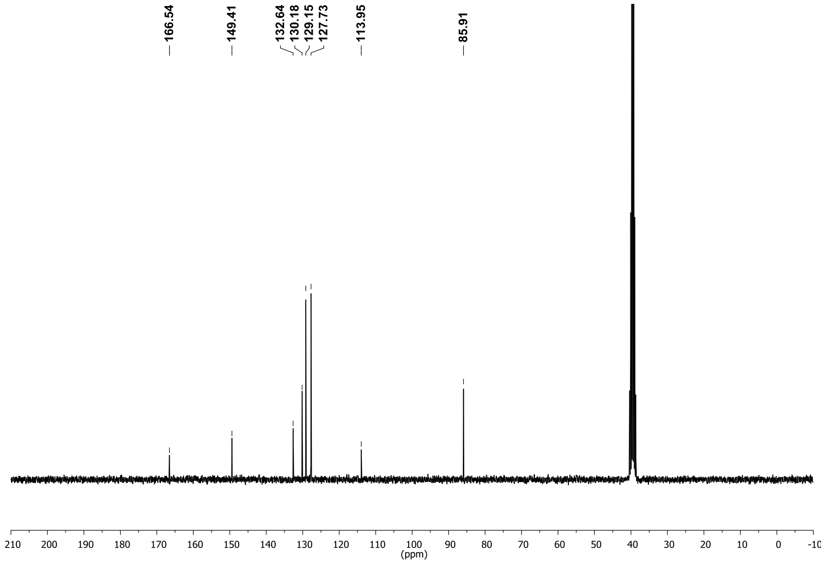

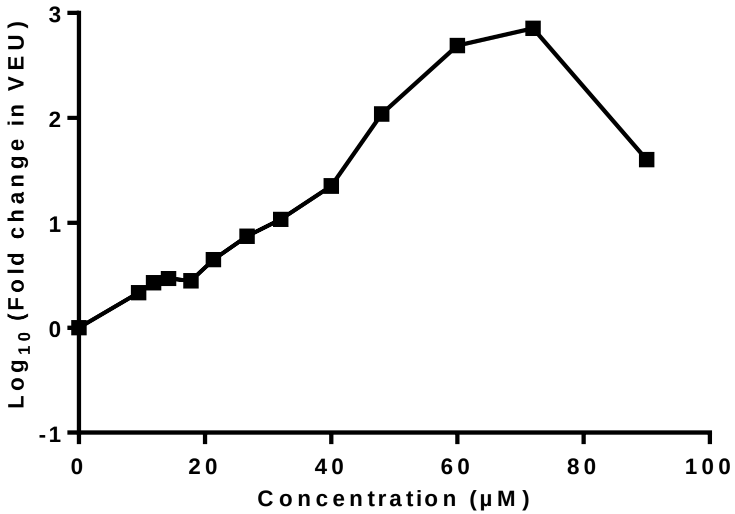

Compound ID: 3

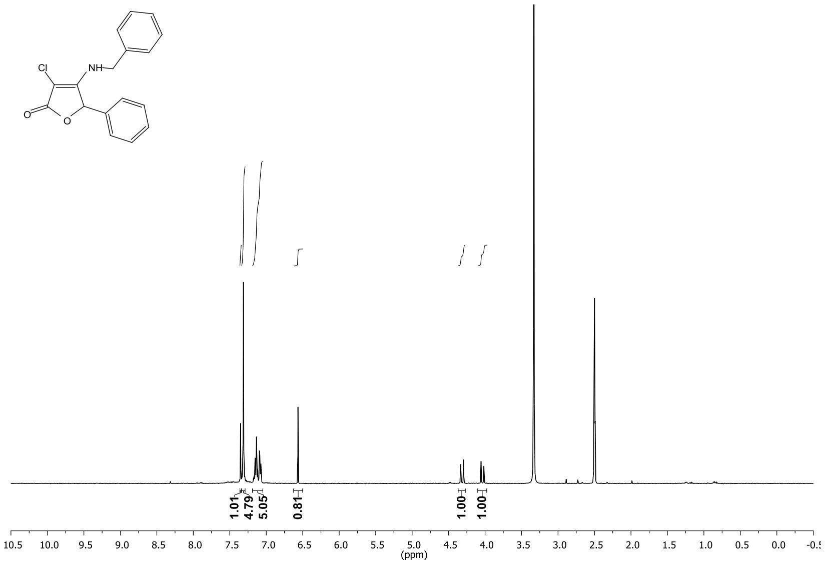

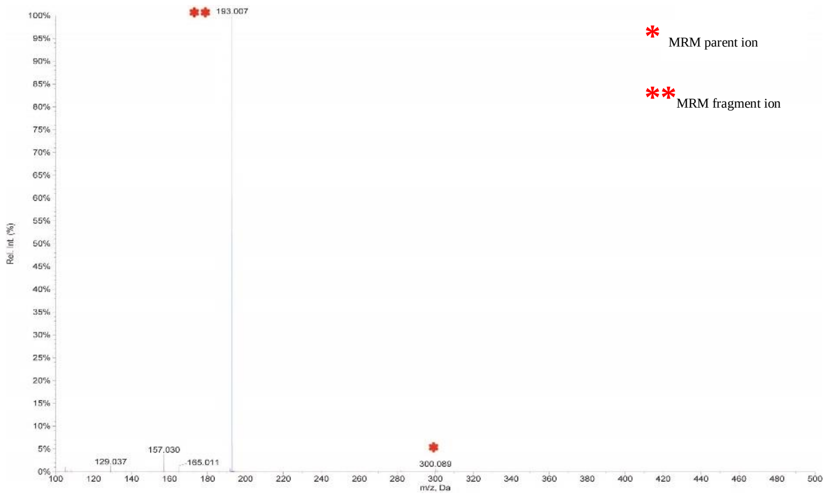

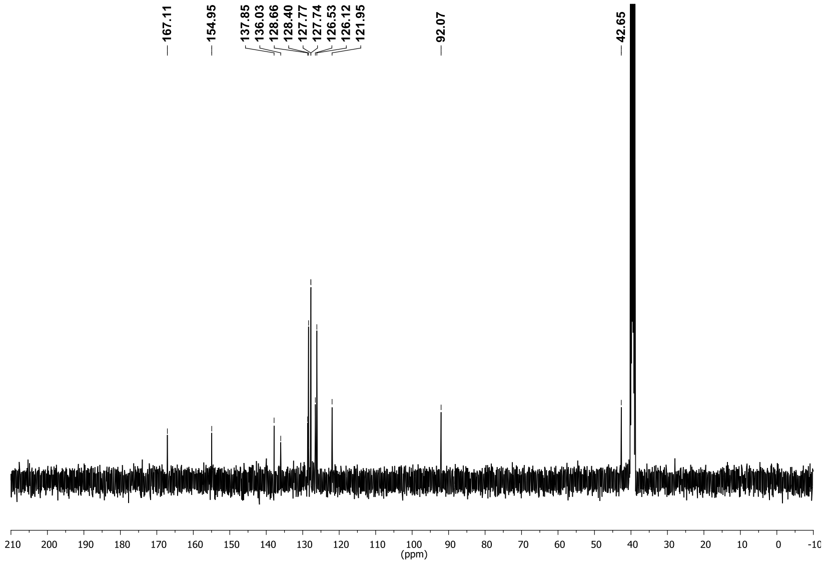

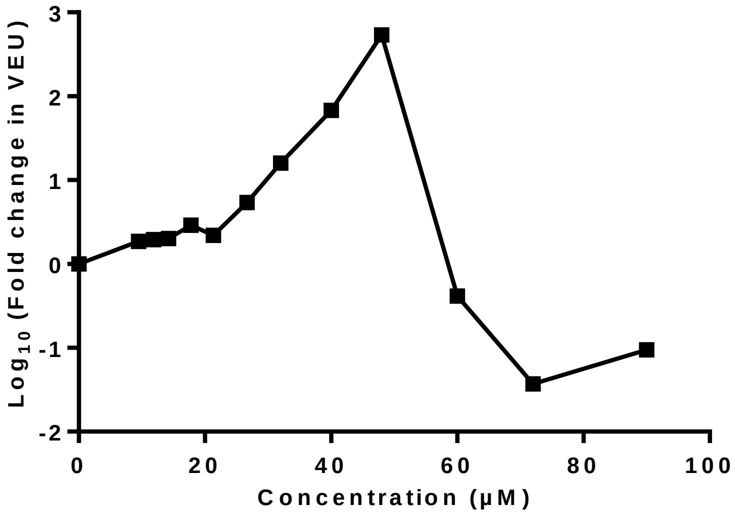

Compound ID: 4

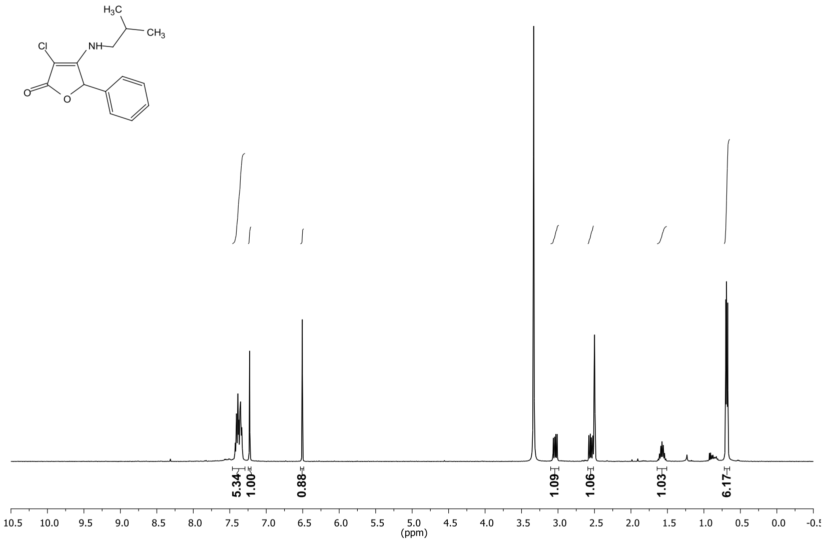

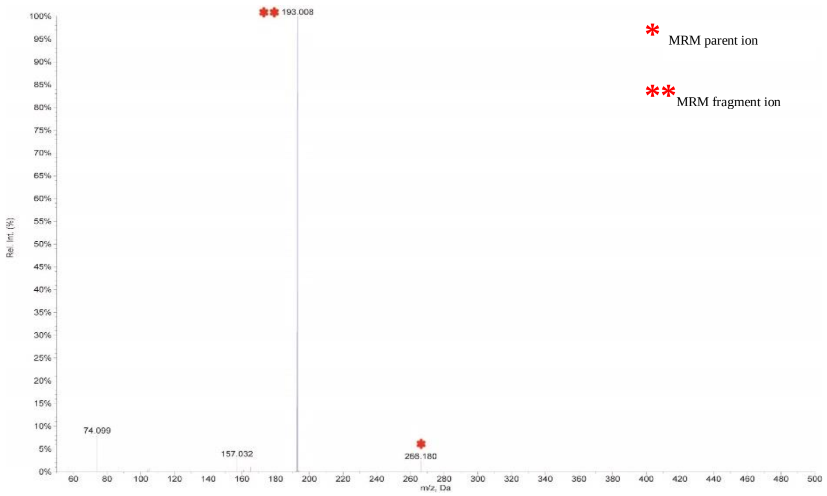

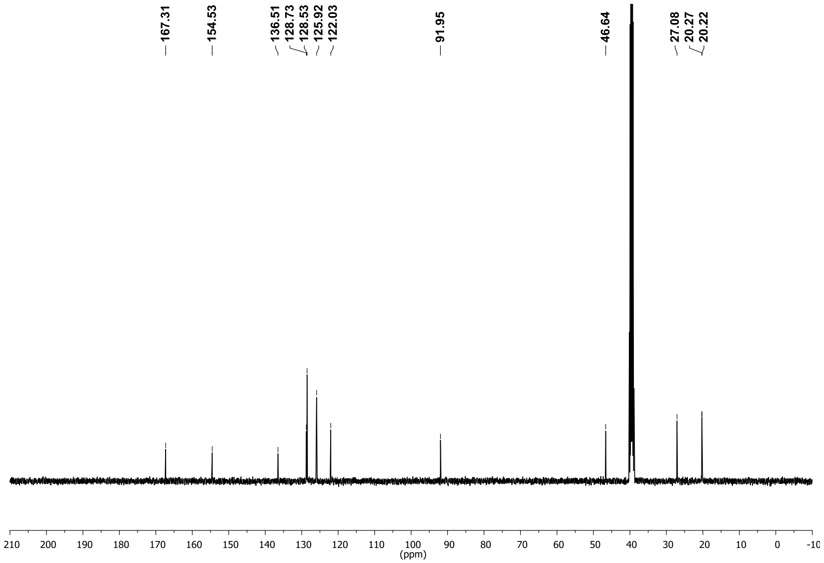

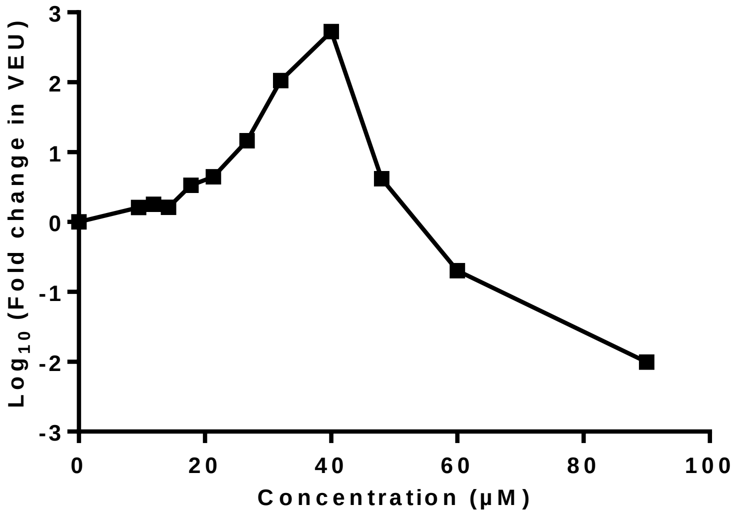

Compound ID: 5

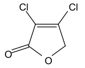

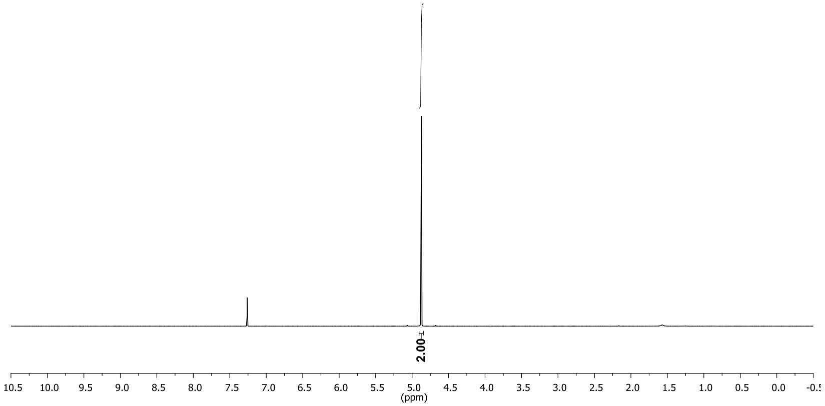

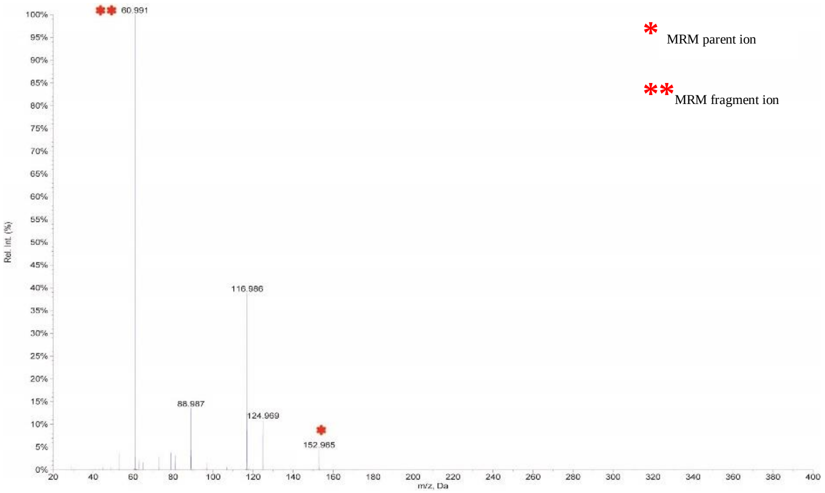

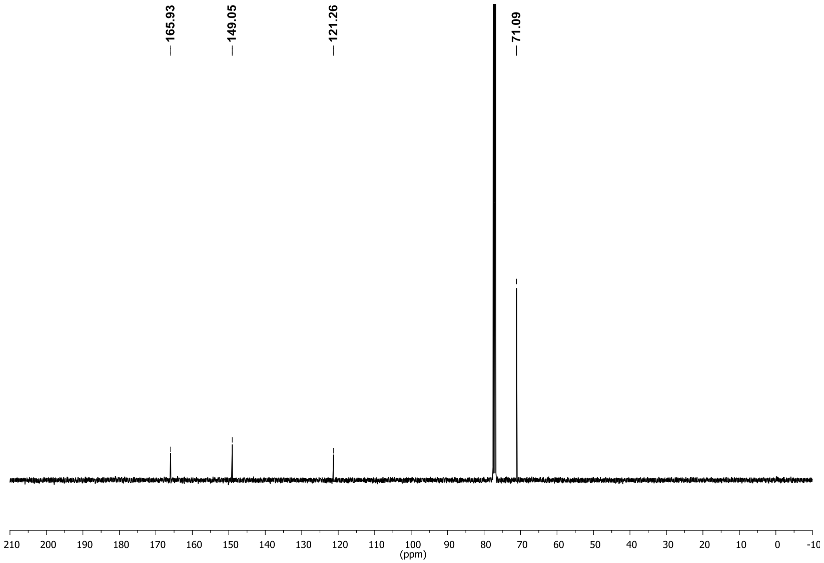

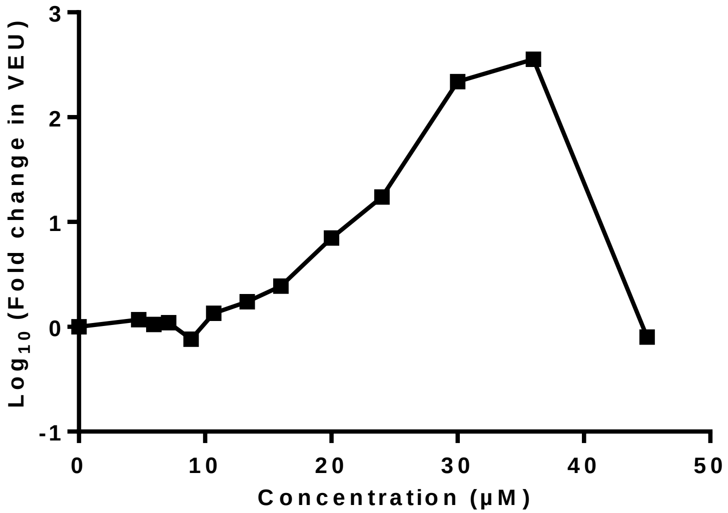

Compound ID: 6

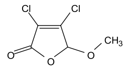

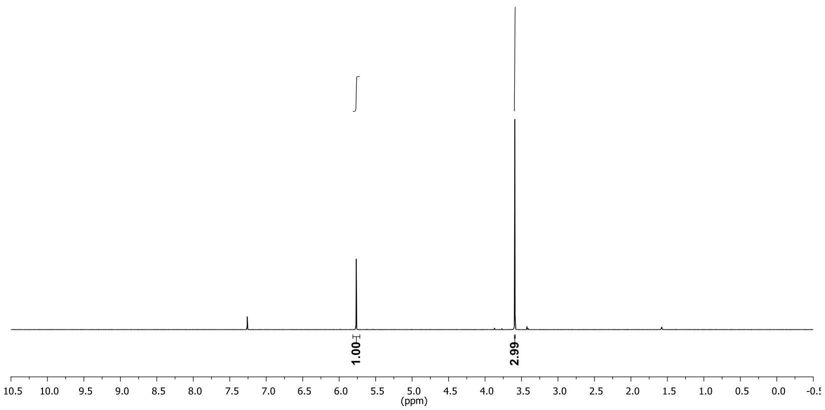

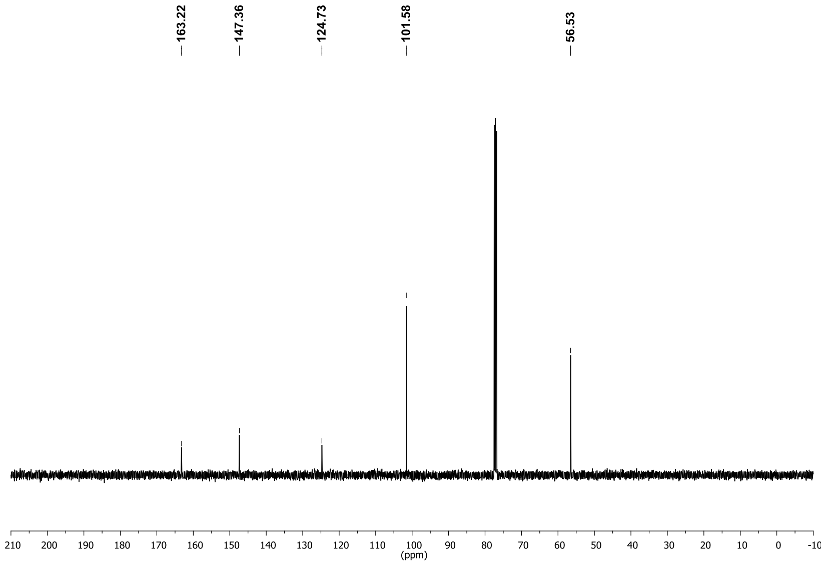

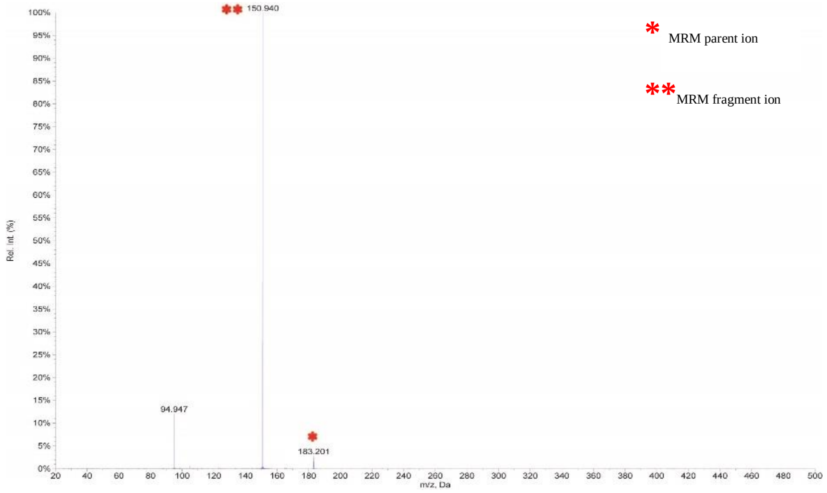

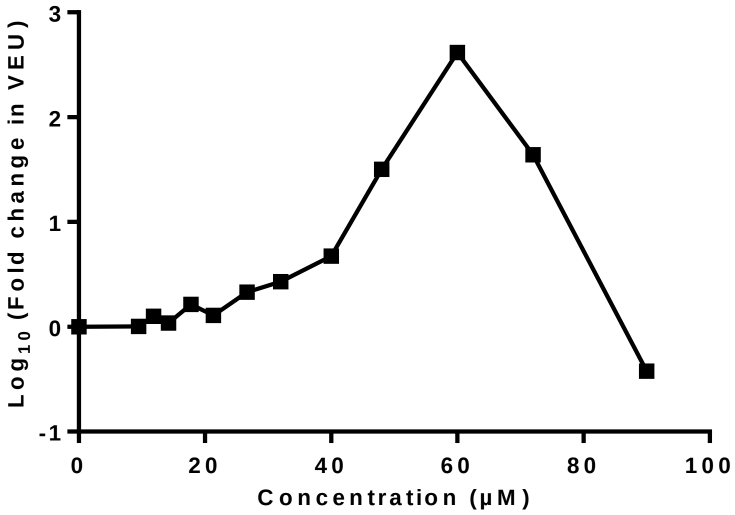

Compound ID: 7

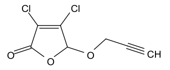

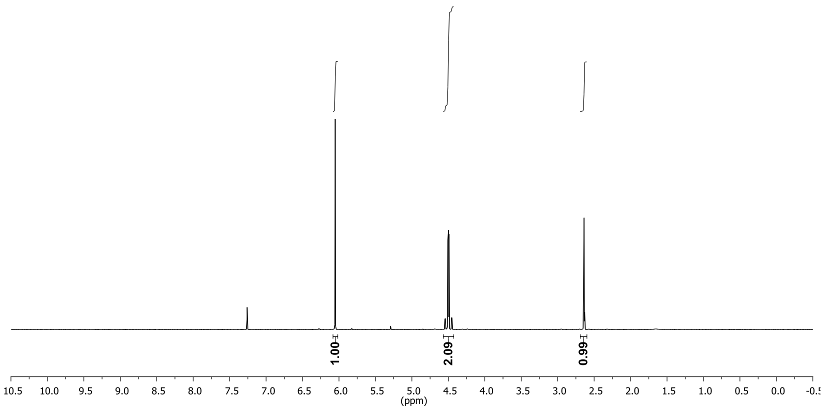

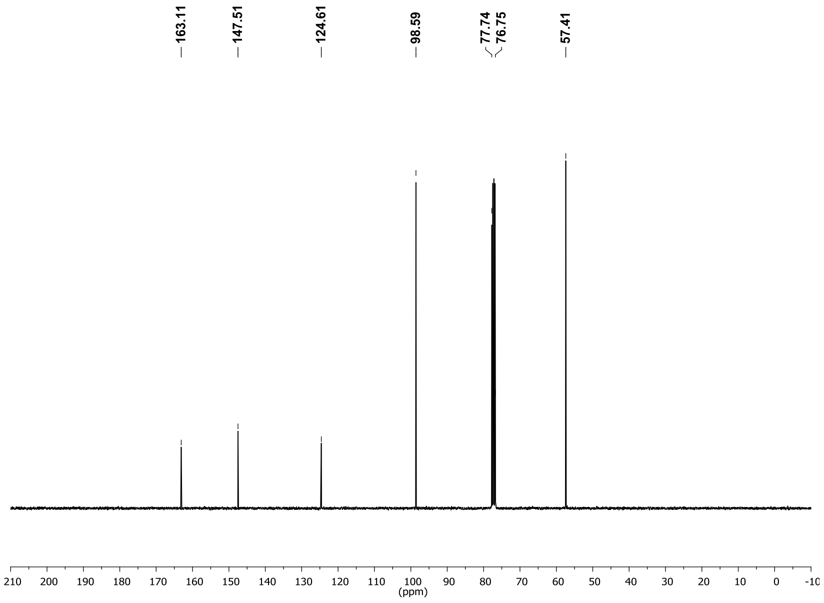

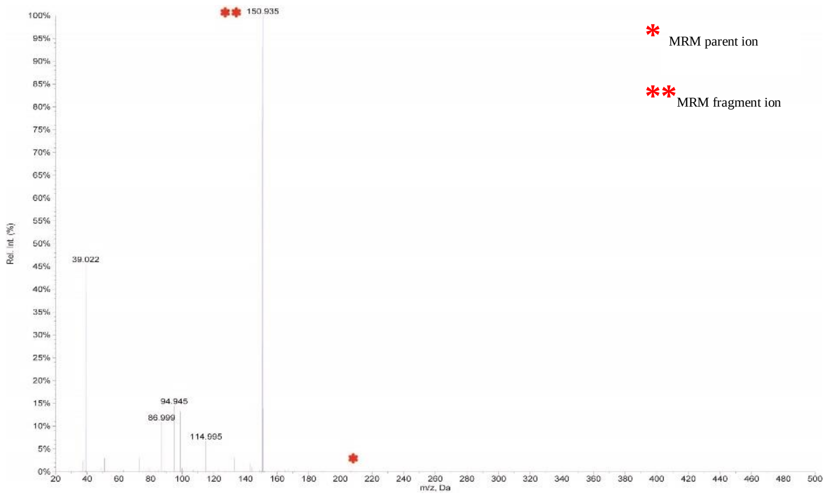

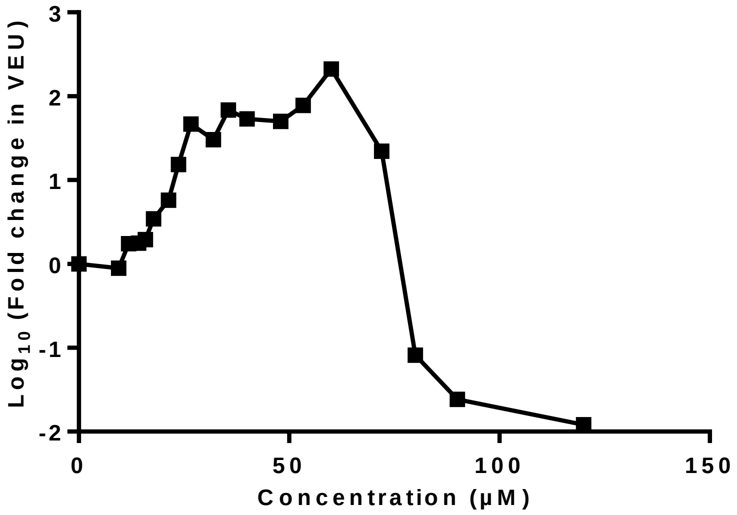

Compound ID: 8

Compound purchased from a commercial supplier.

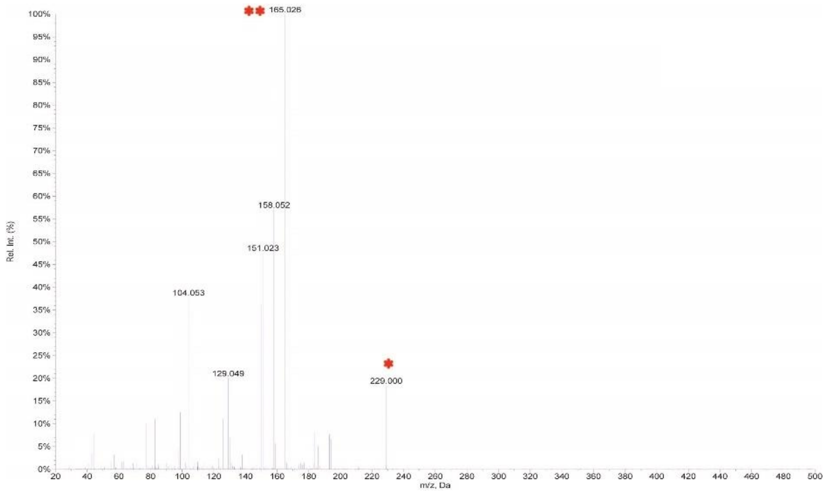

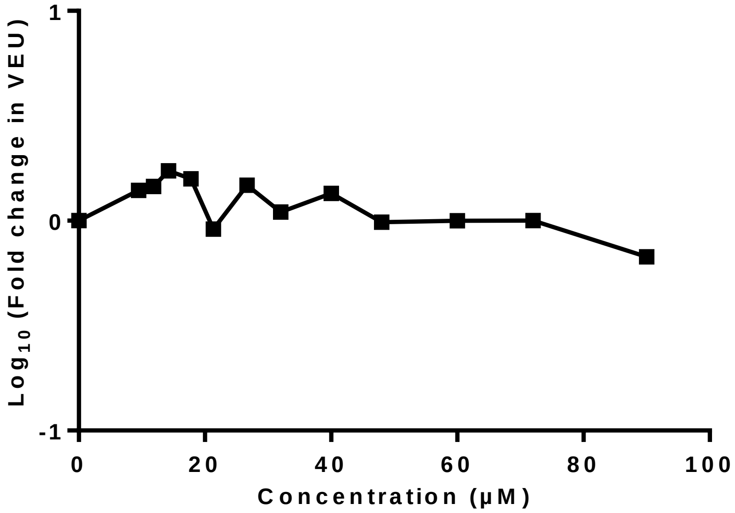

Compound ID: 9

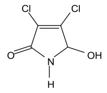

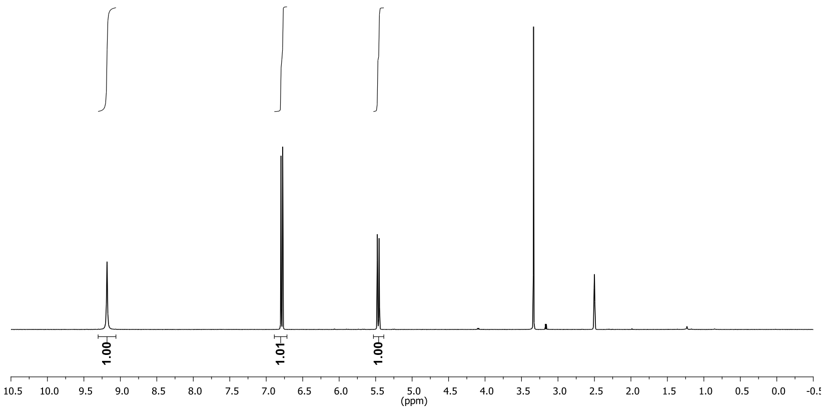

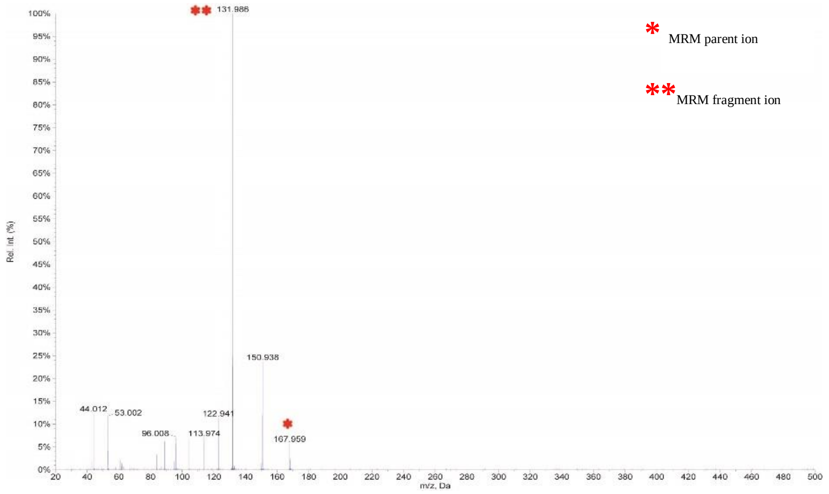

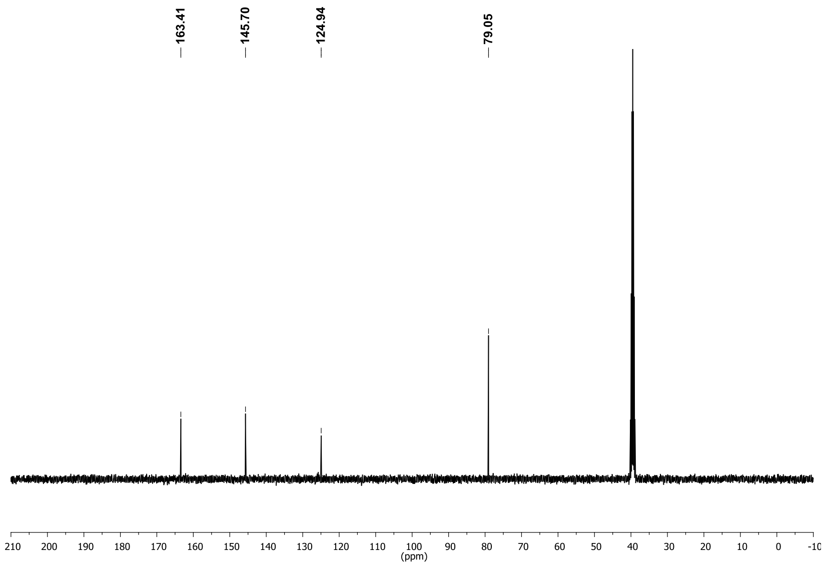

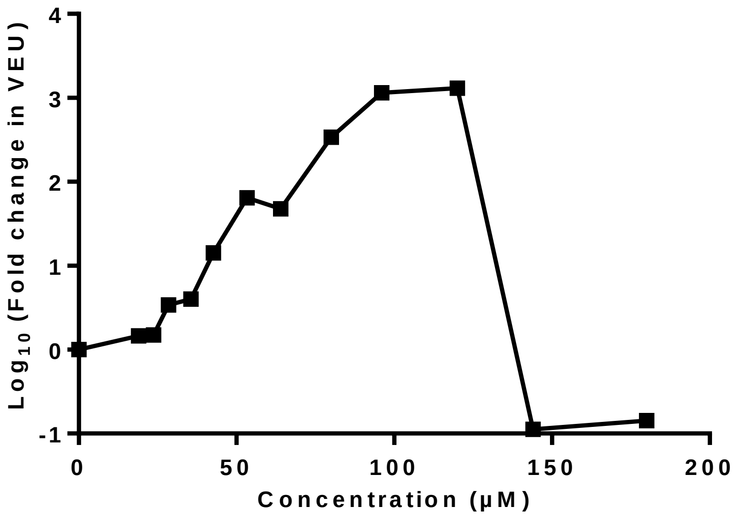

Compound ID: 10

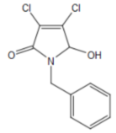

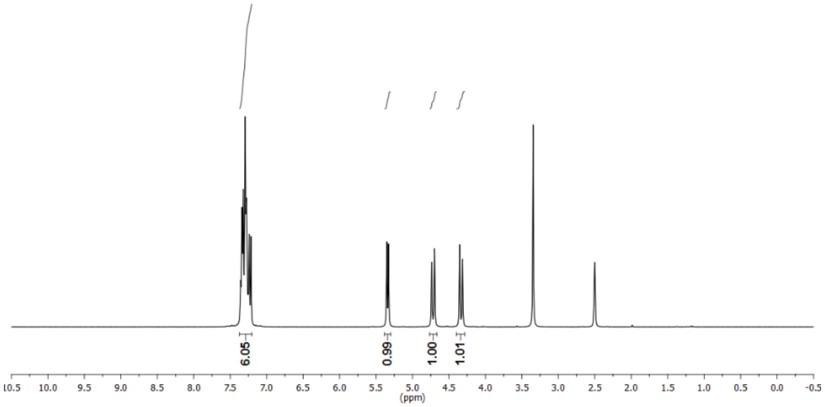

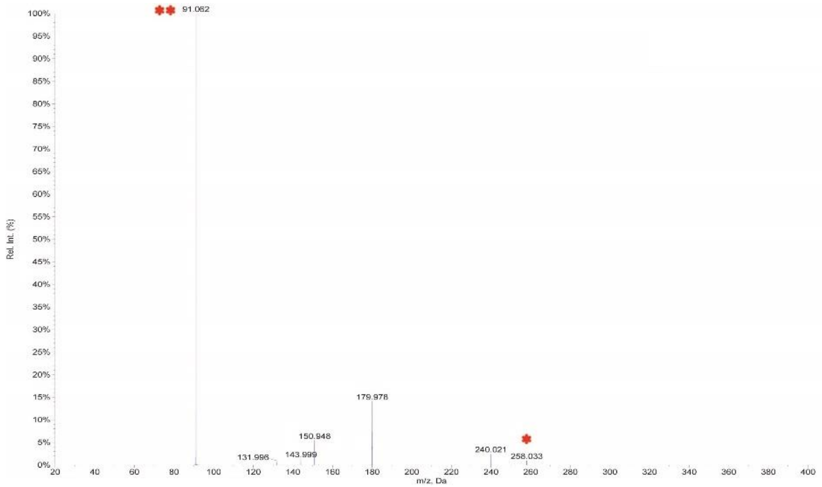

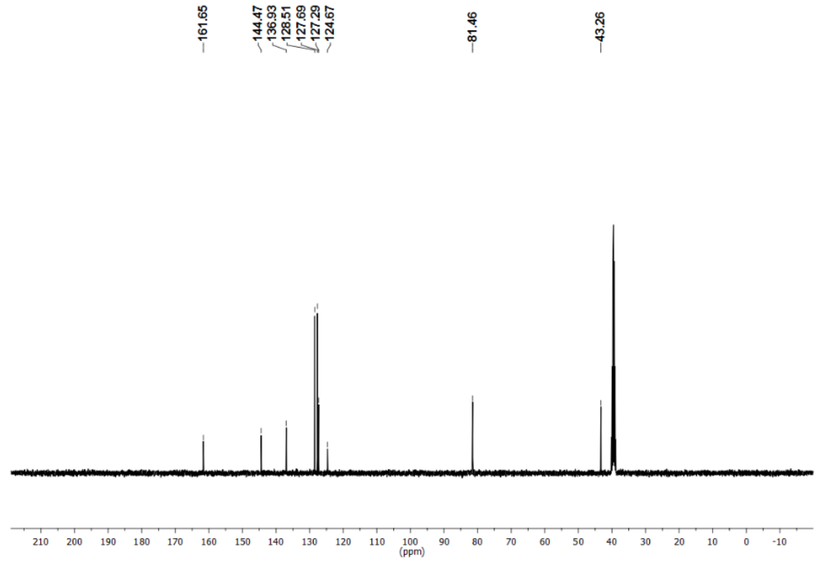

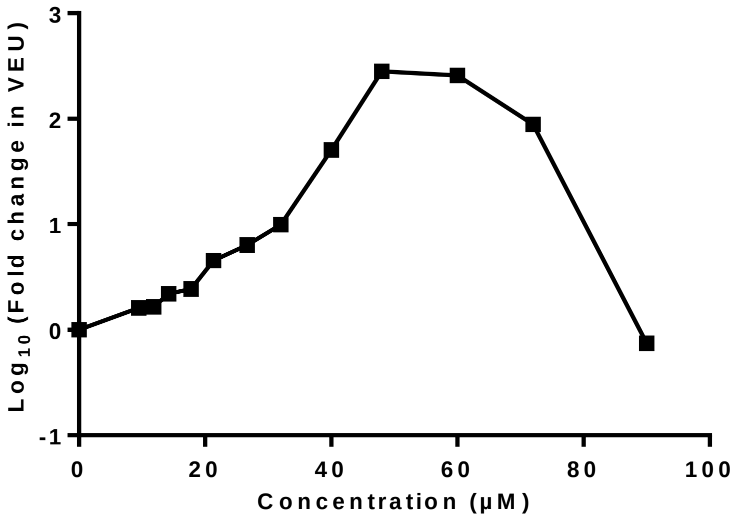

Compound ID: 11

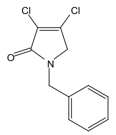

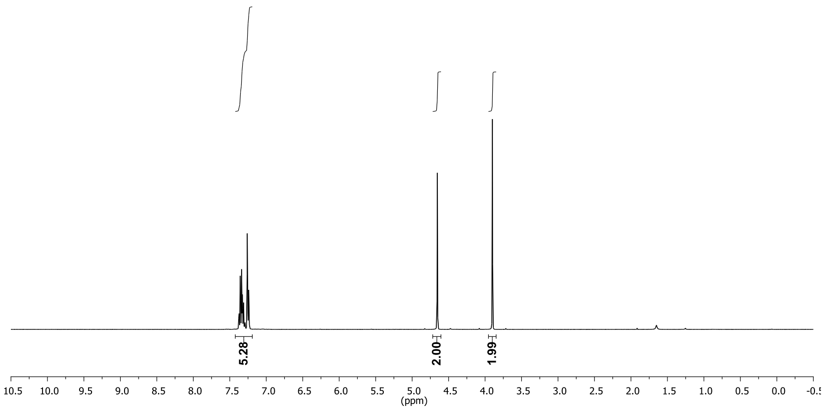

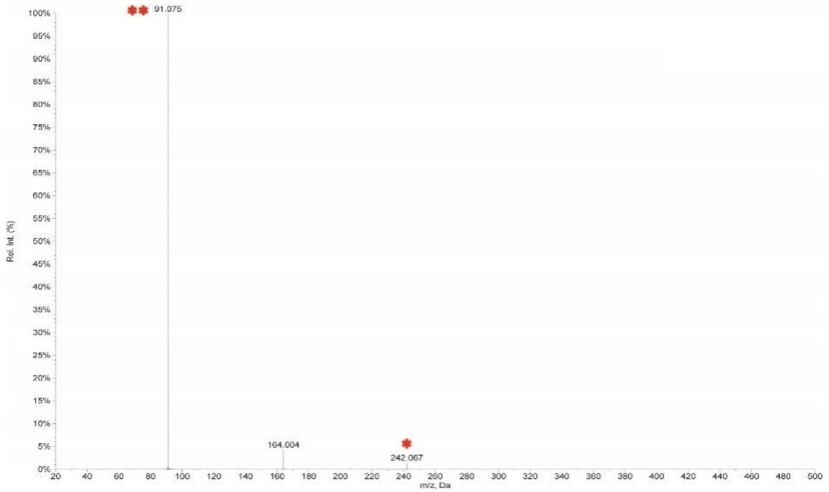

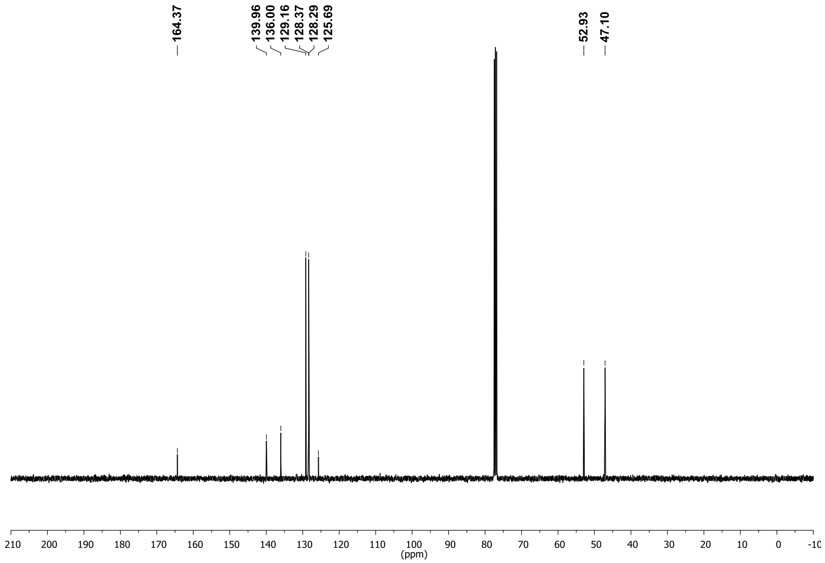

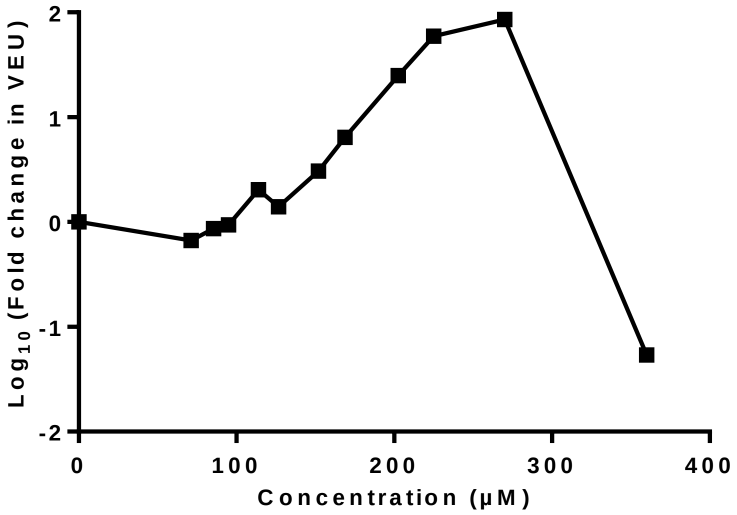

Compound ID: 12

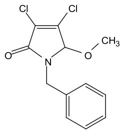

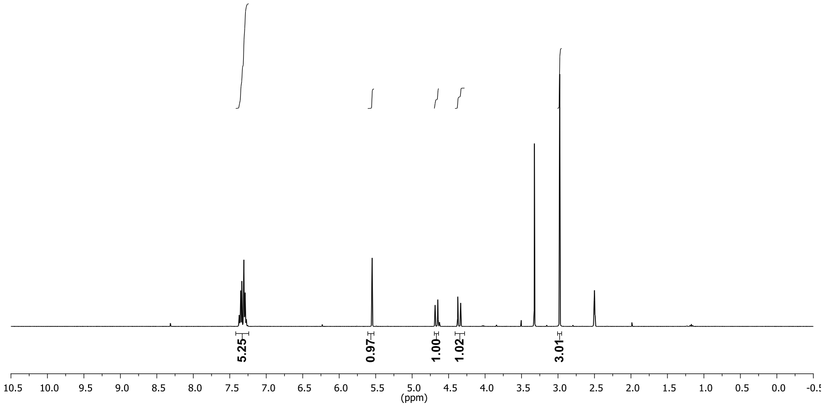

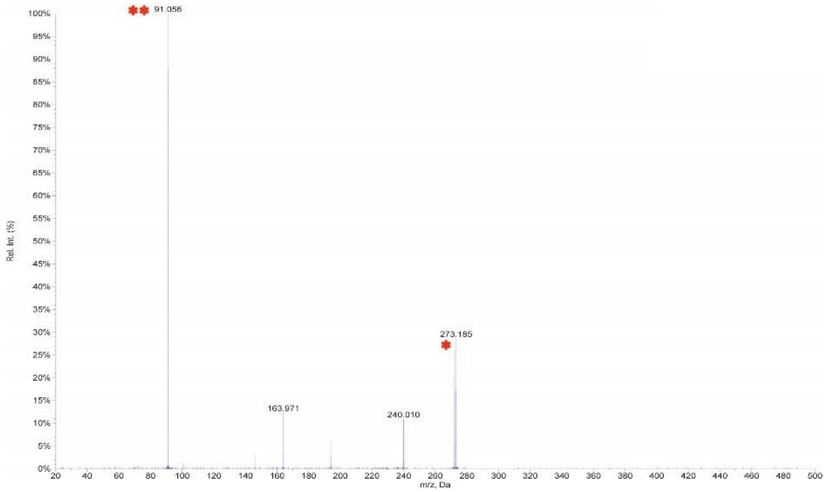

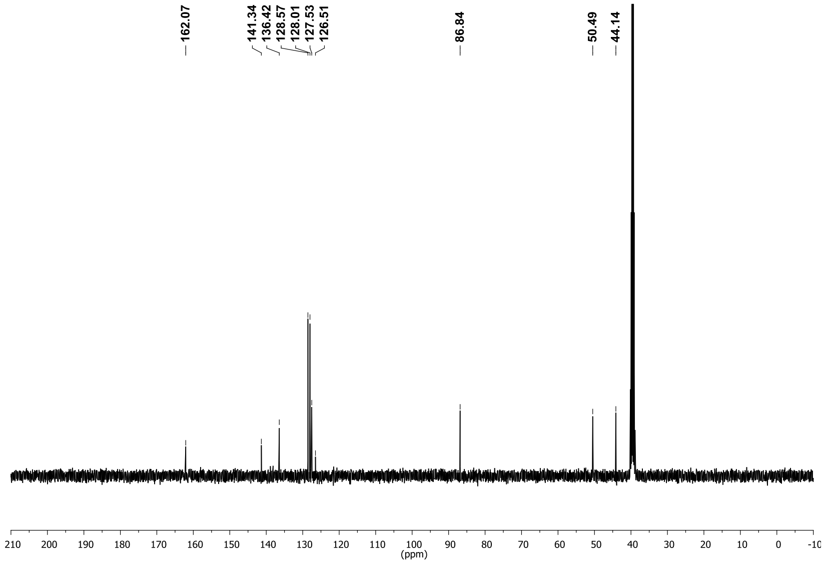

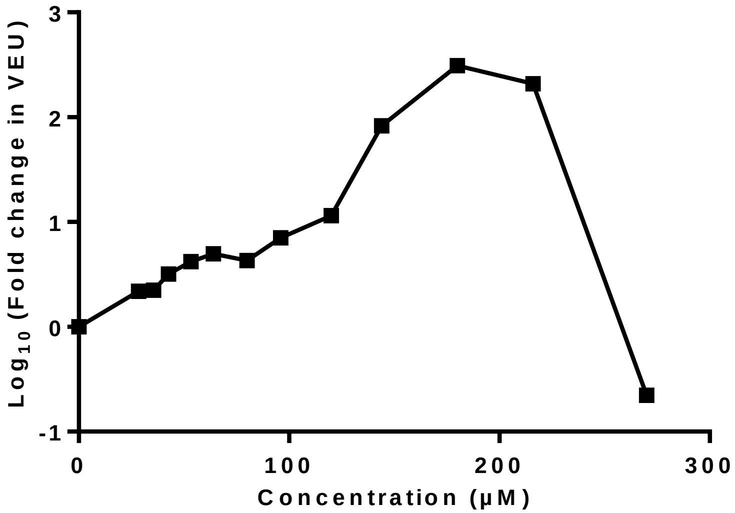

Compound ID: 13

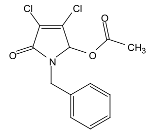

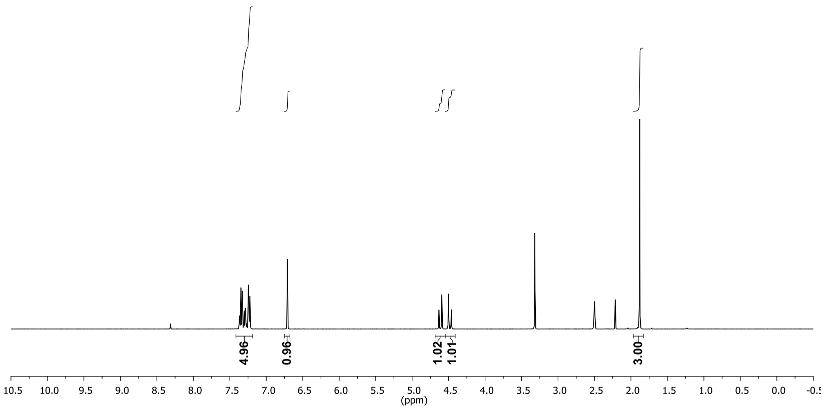

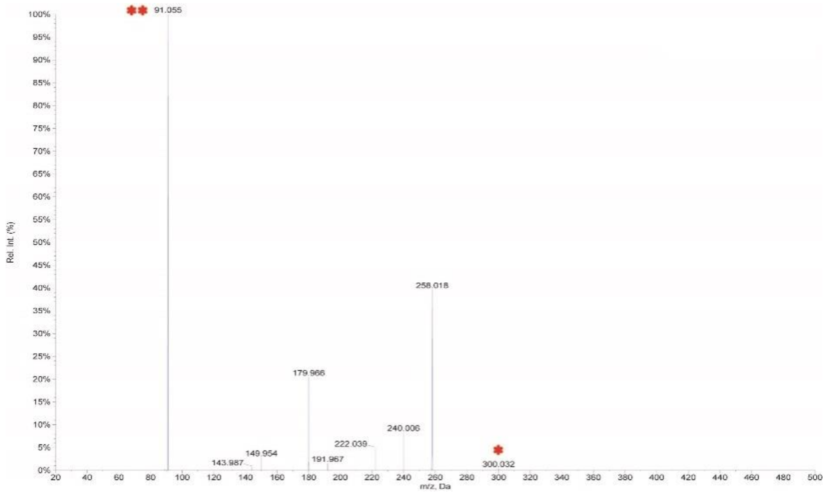

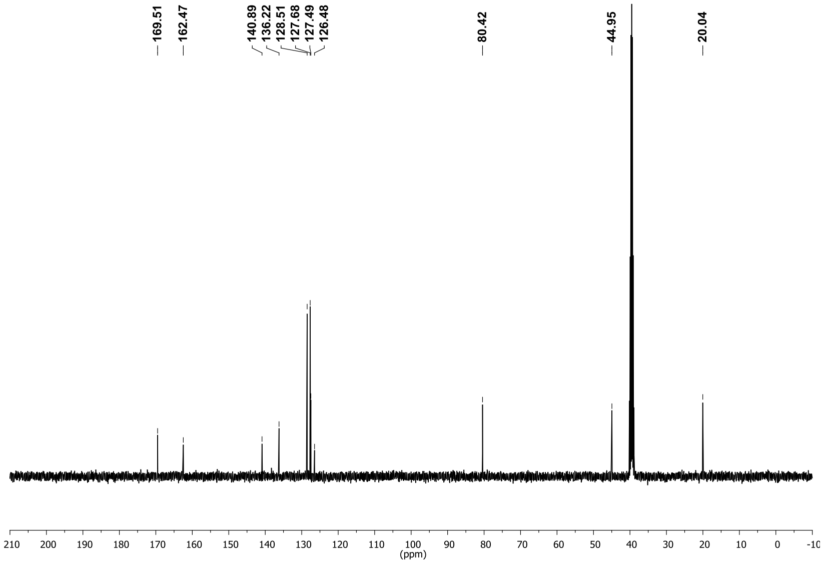

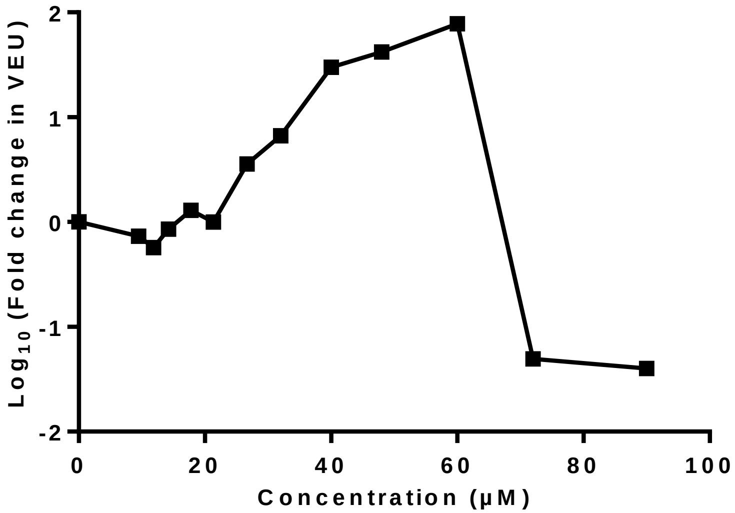

Compound ID: 14

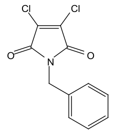

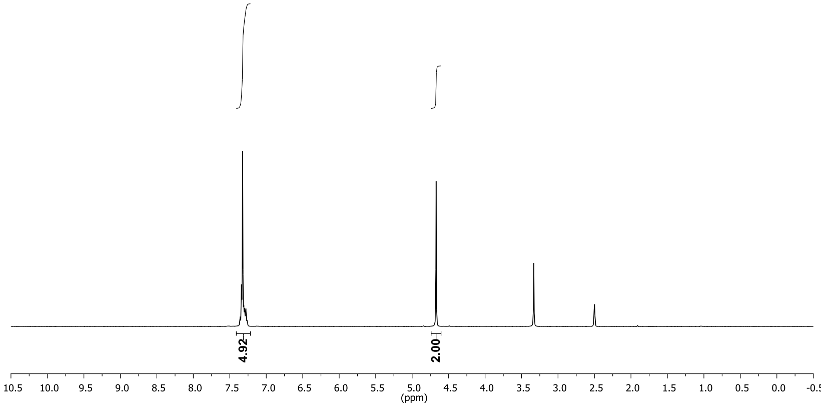

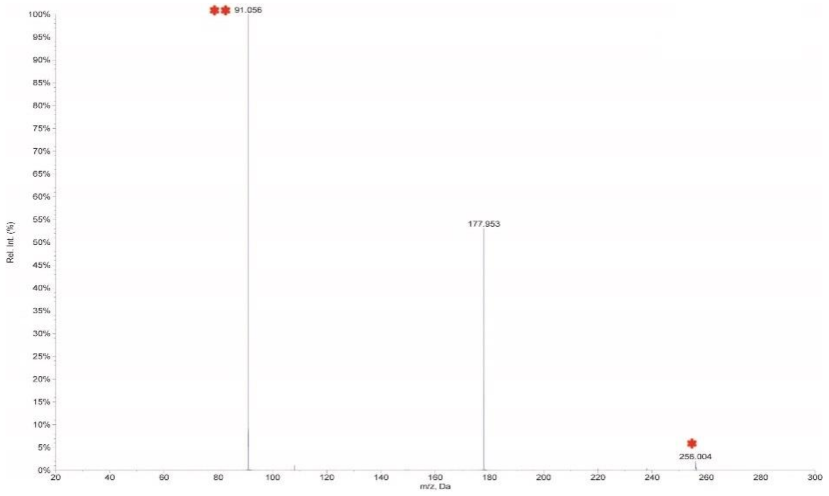

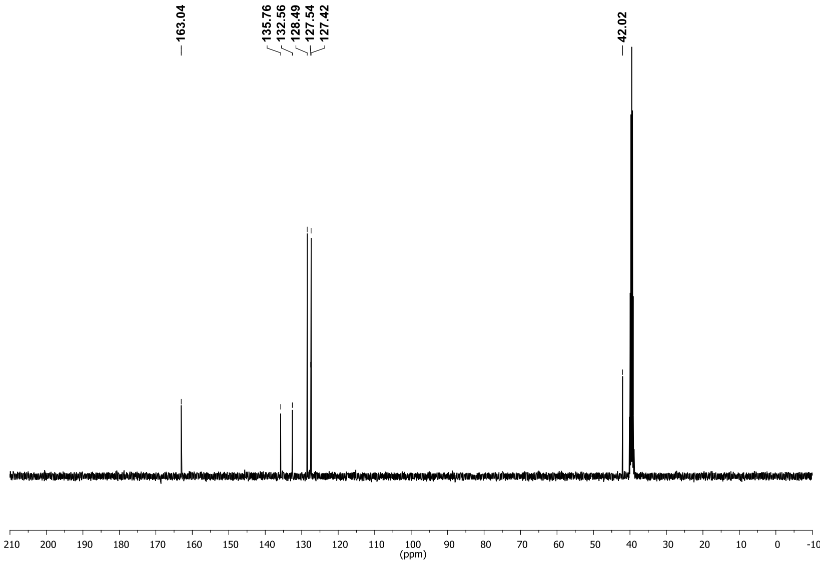

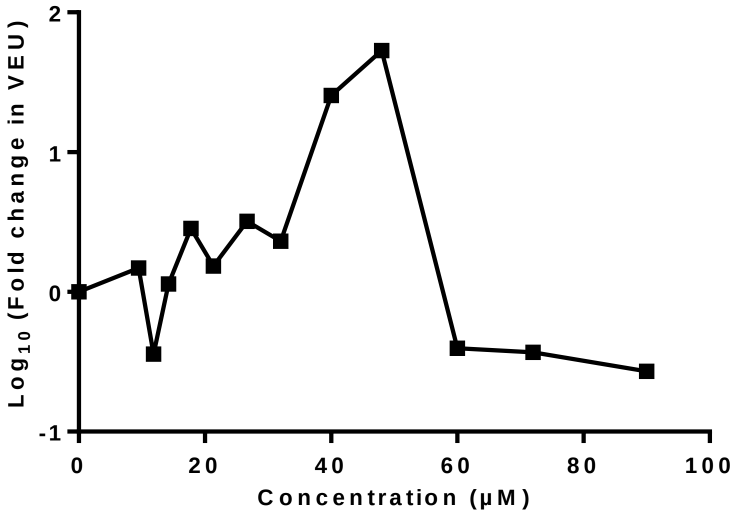

Compound ID: 15

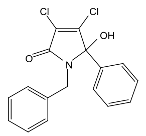

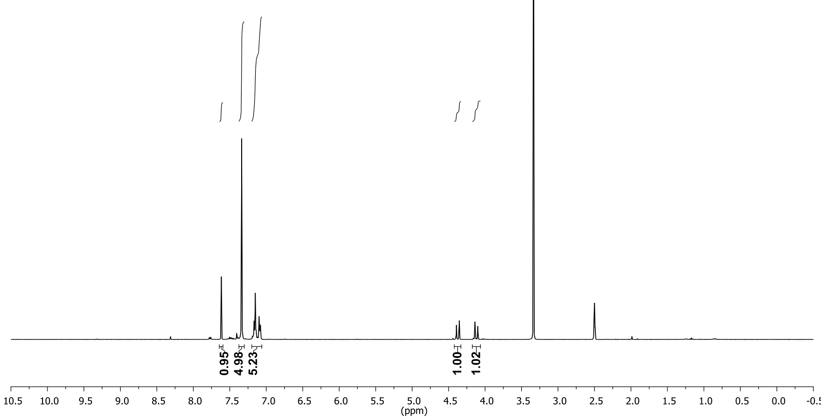

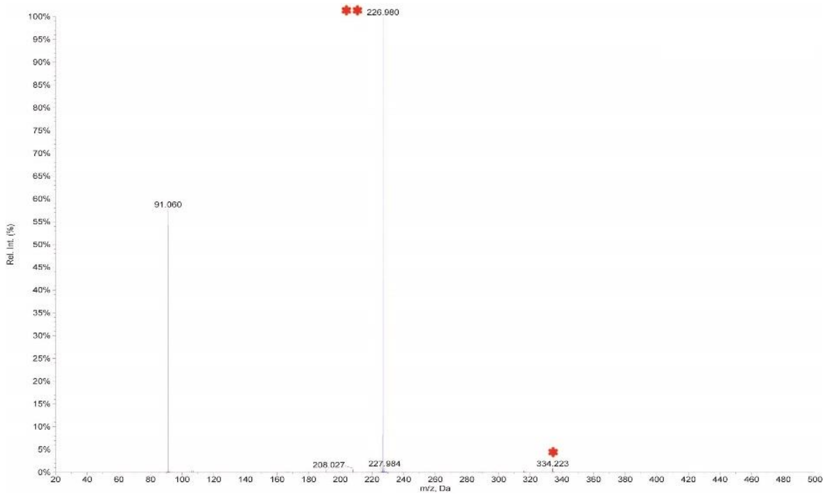

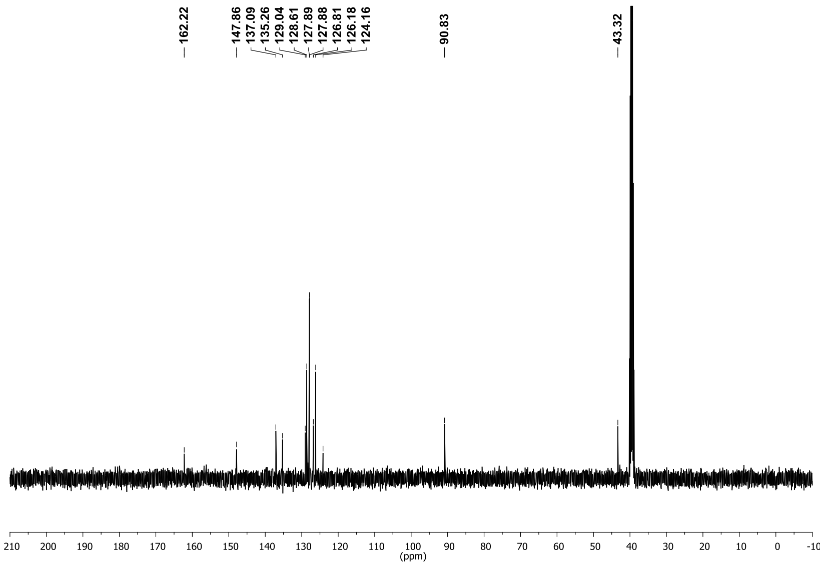

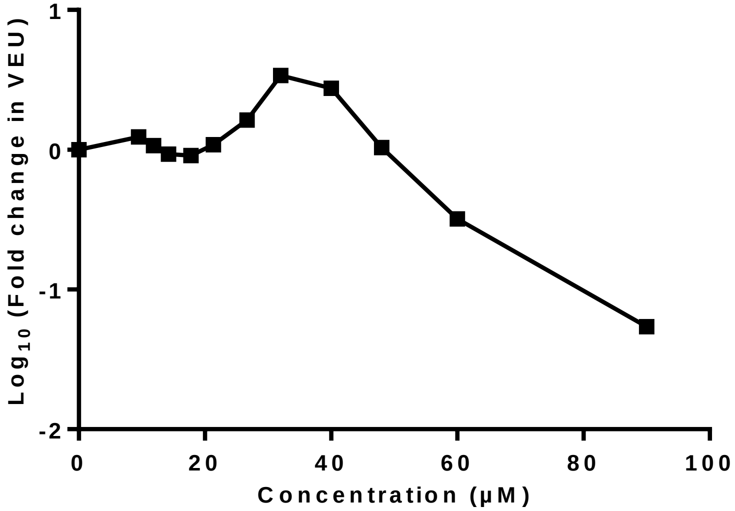

Compound ID: 16

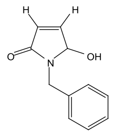

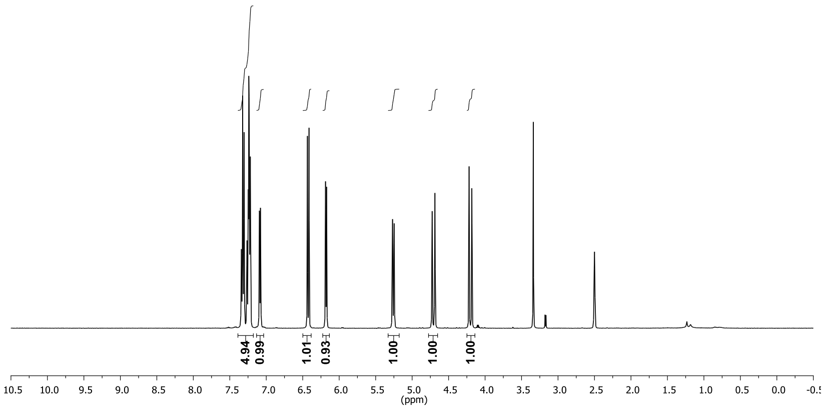

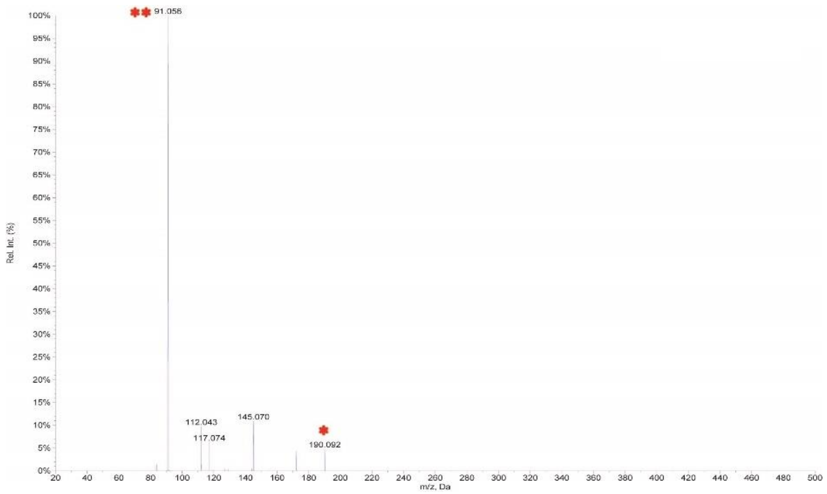

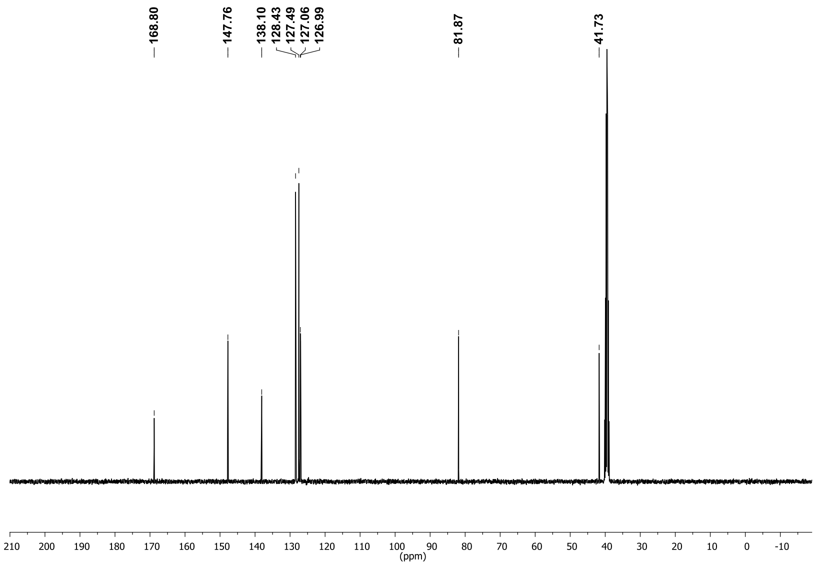

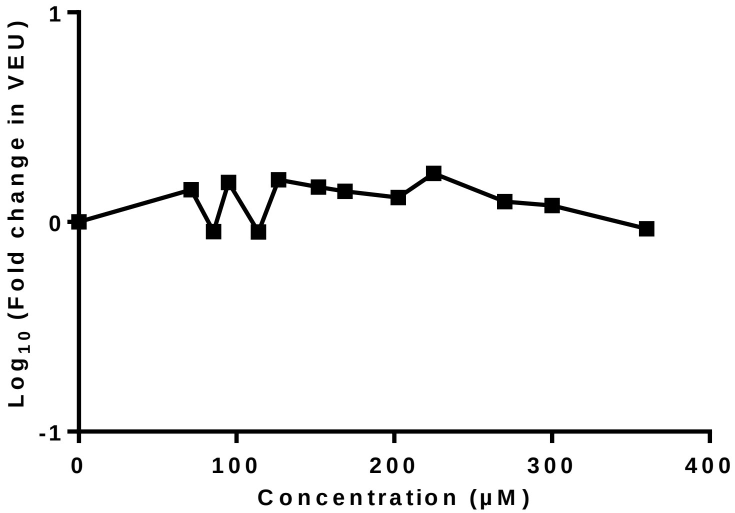

Compound ID: 17

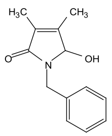

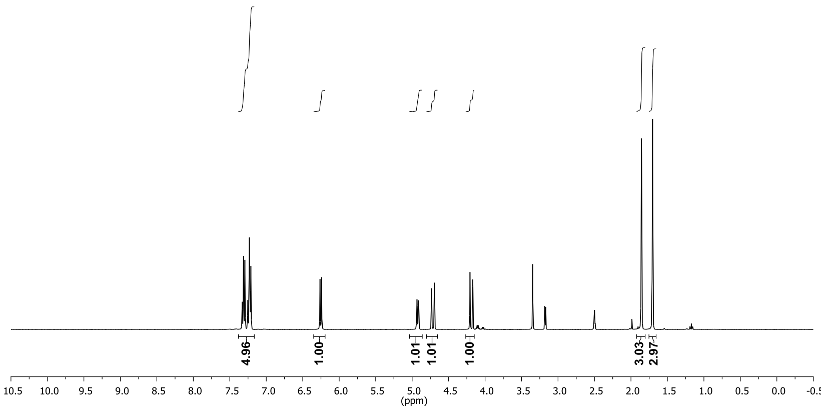

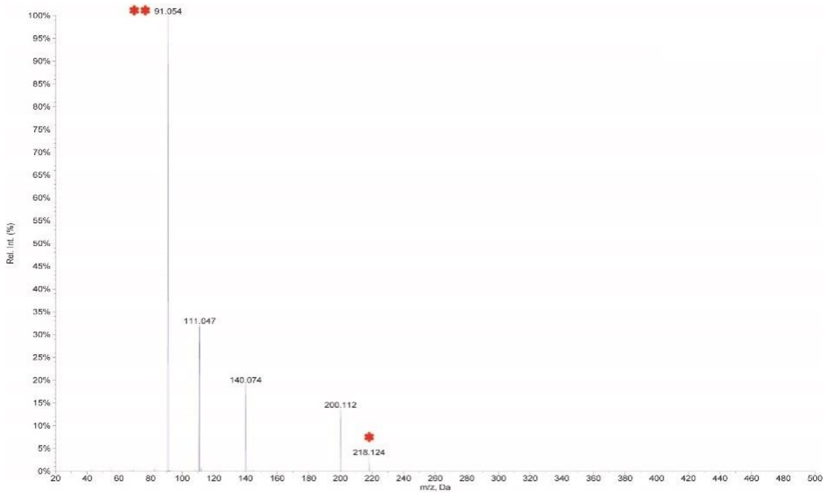

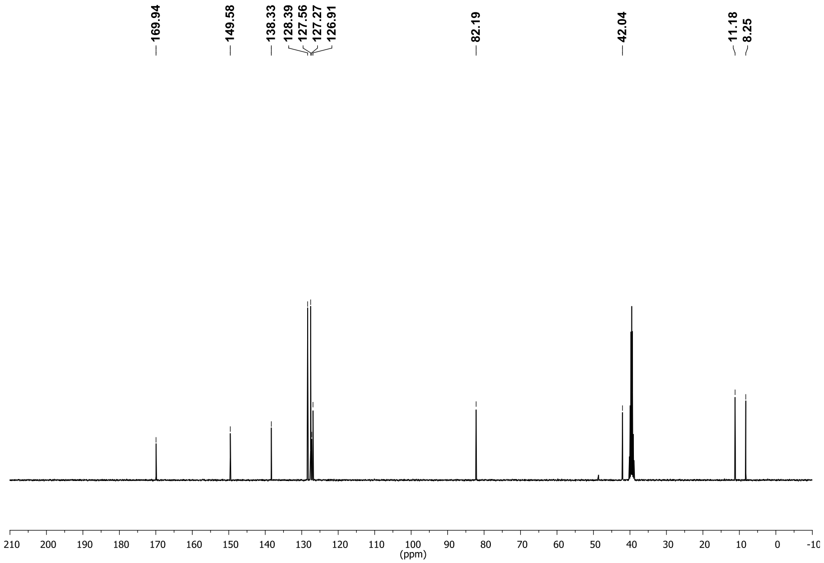

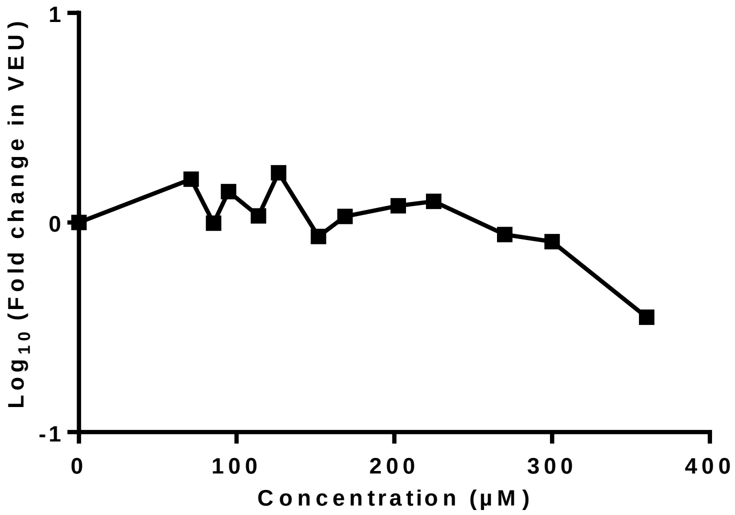

Compound ID: 18

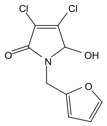

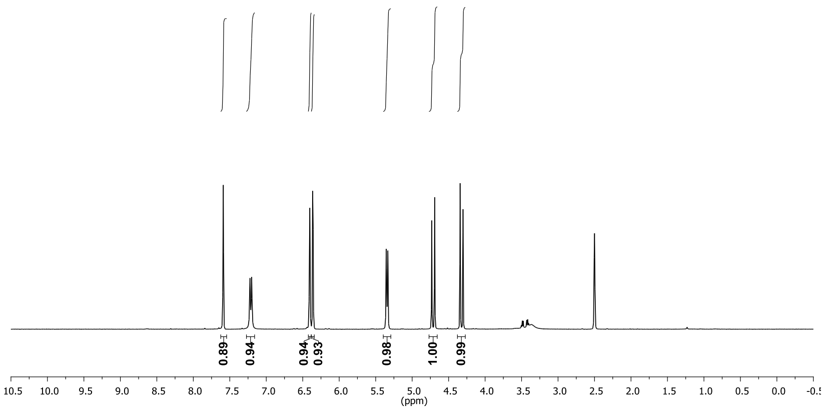

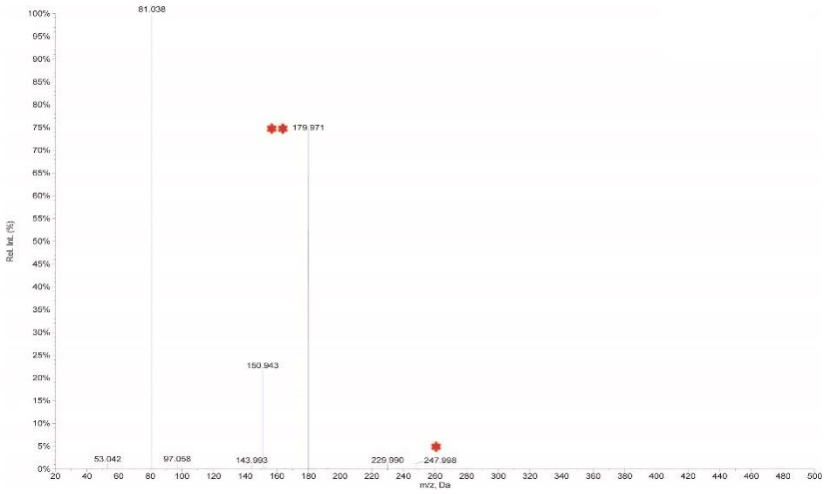

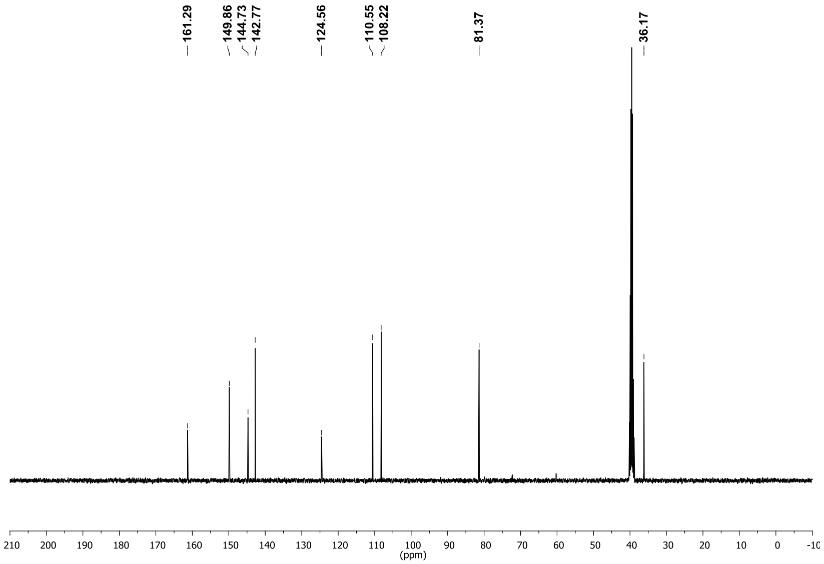

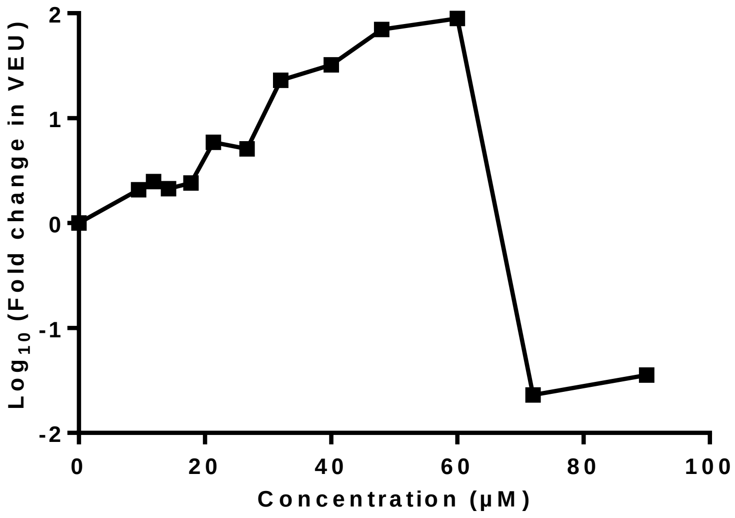

Compound ID: 19

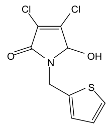

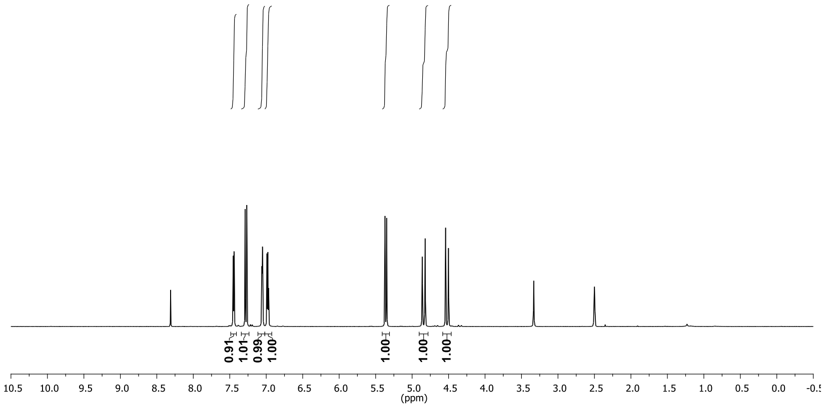

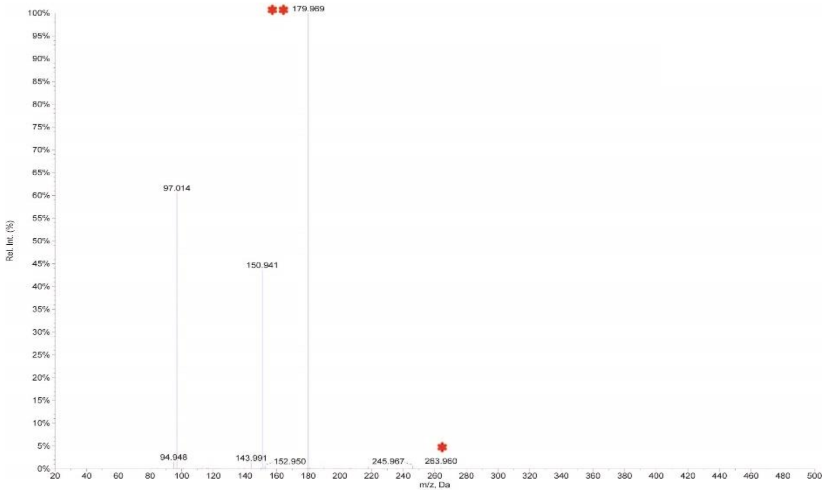

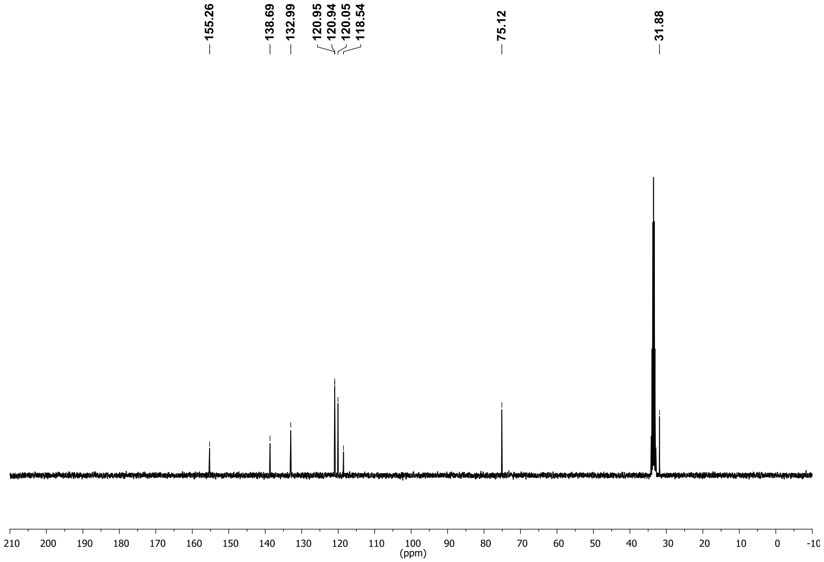

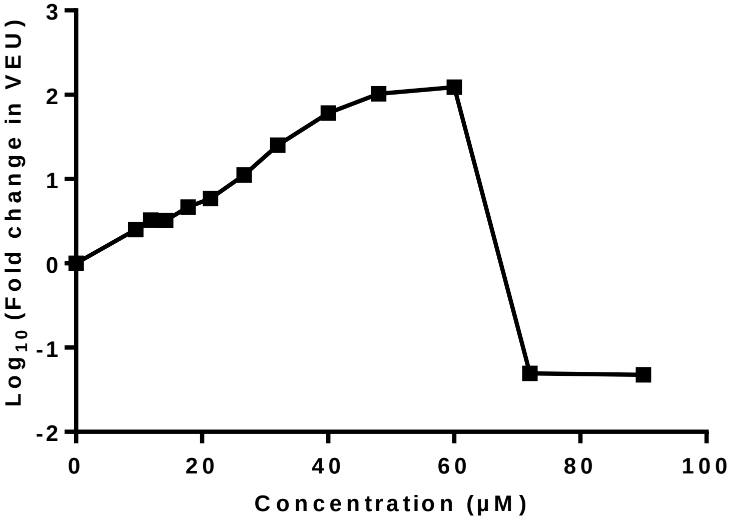

Compound ID: 20

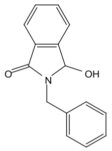

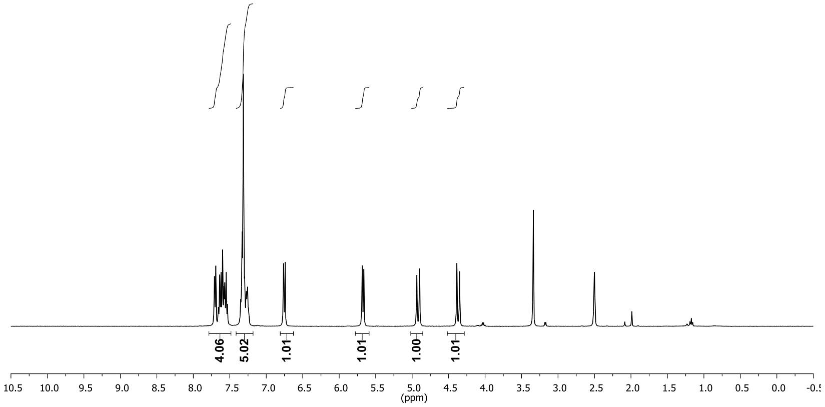

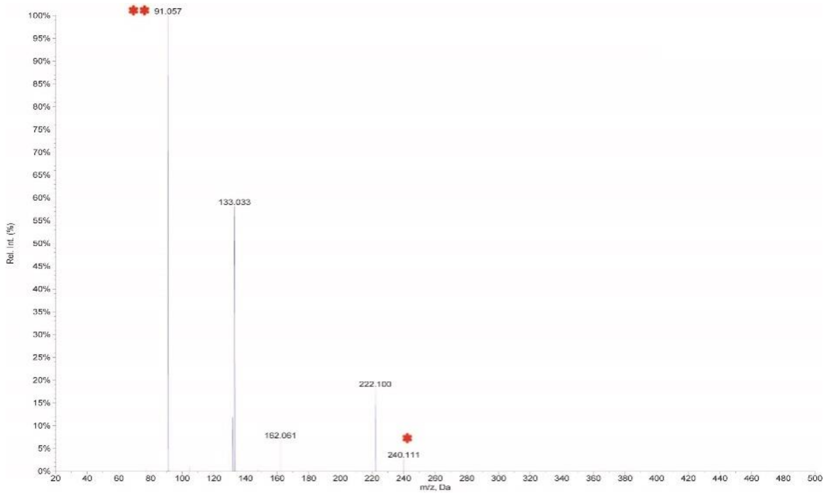

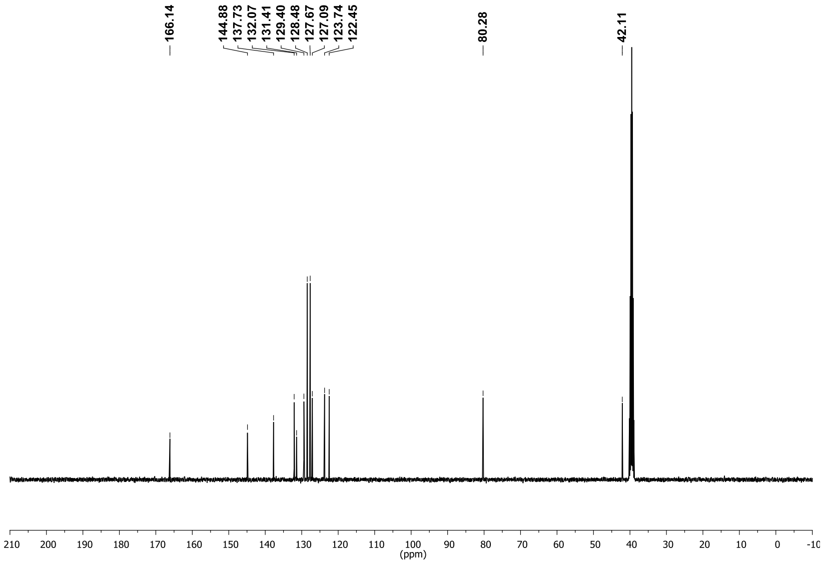

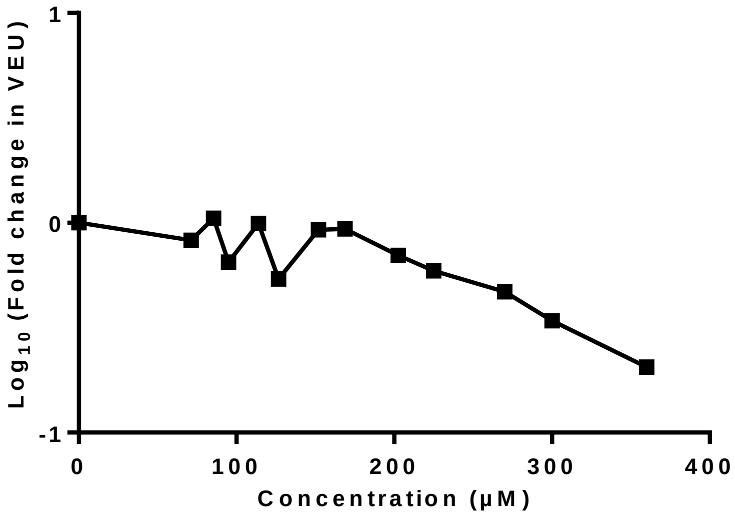

Compound ID: 21

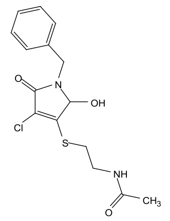

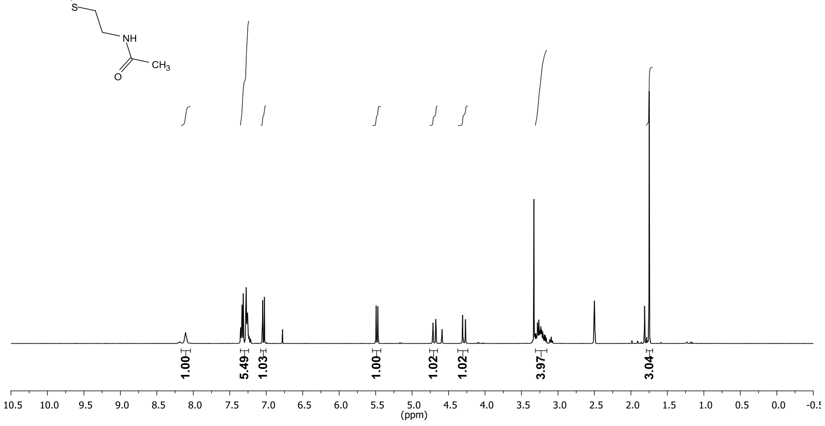

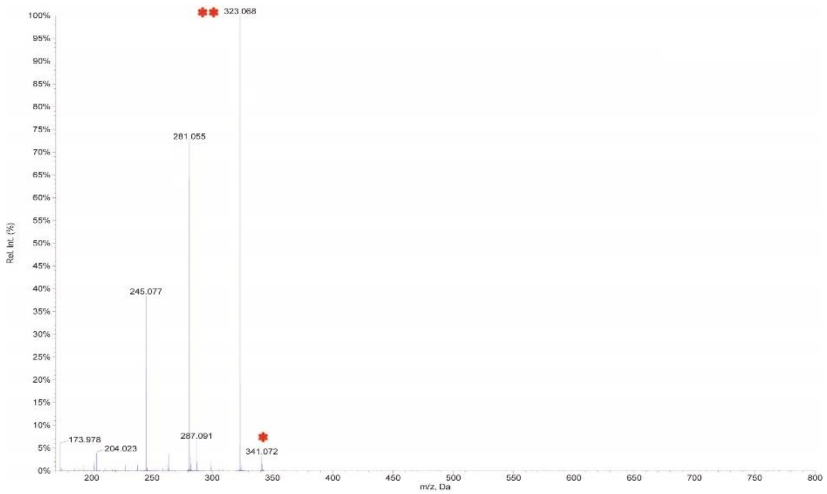

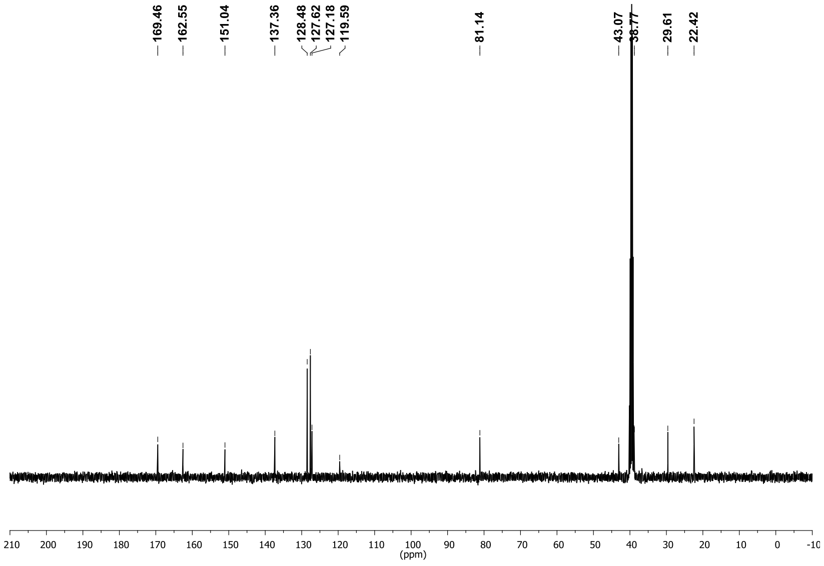

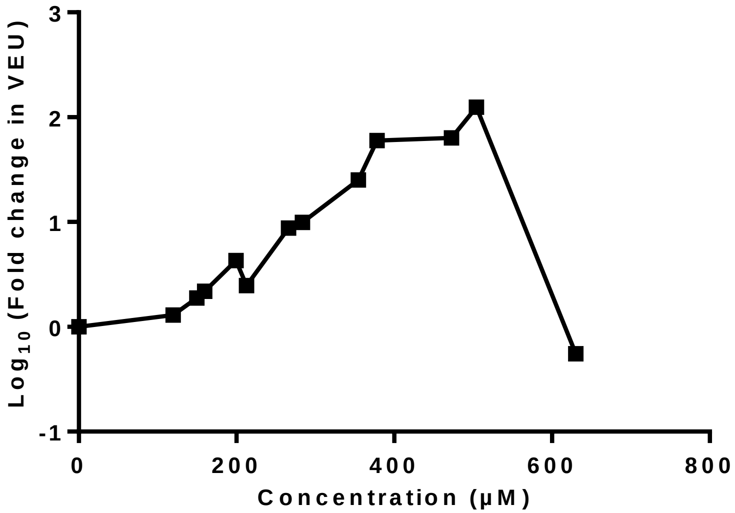

Compound ID: 22

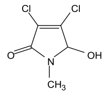

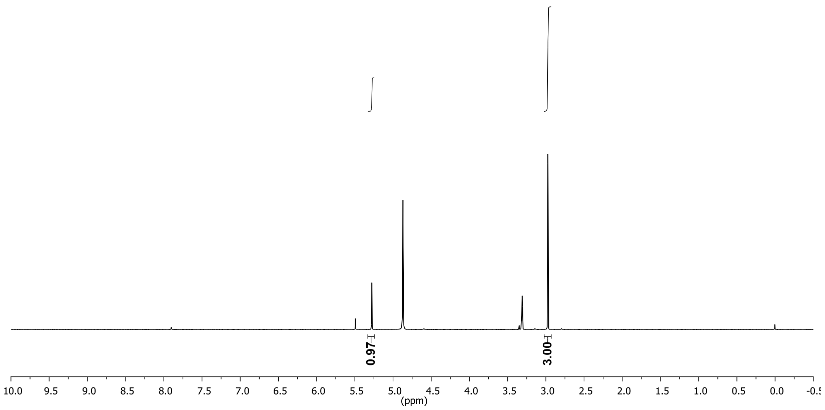

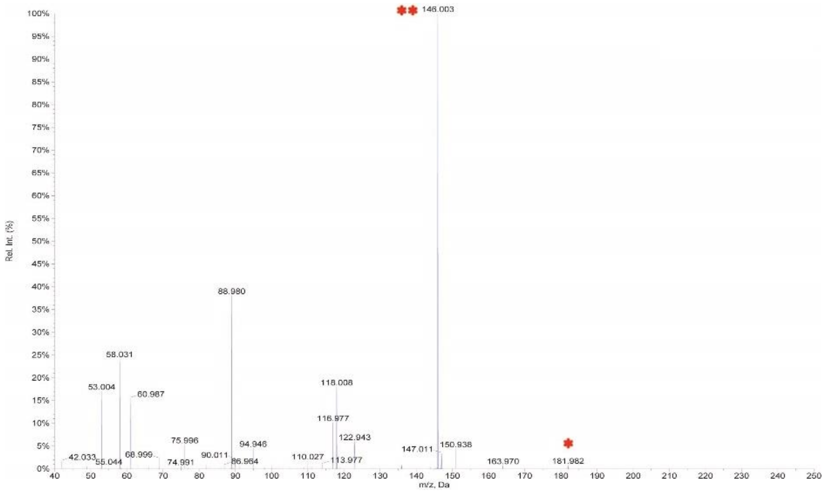

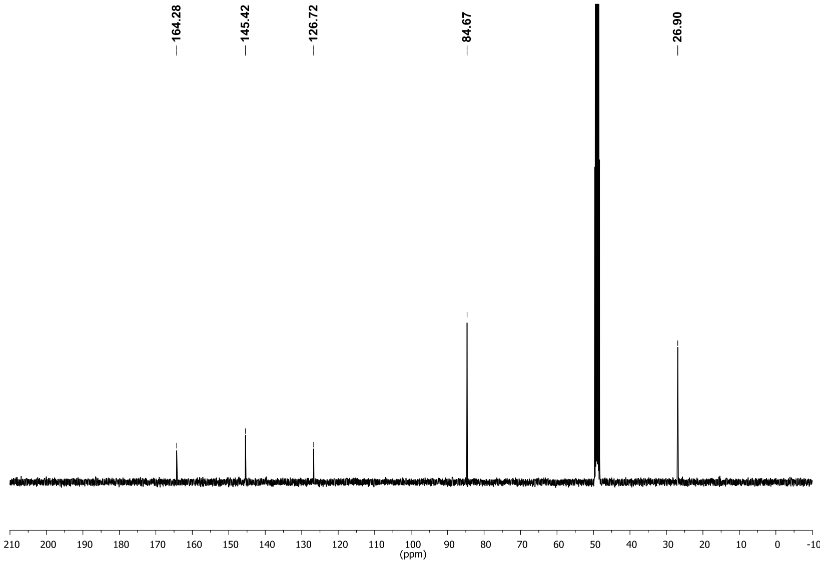

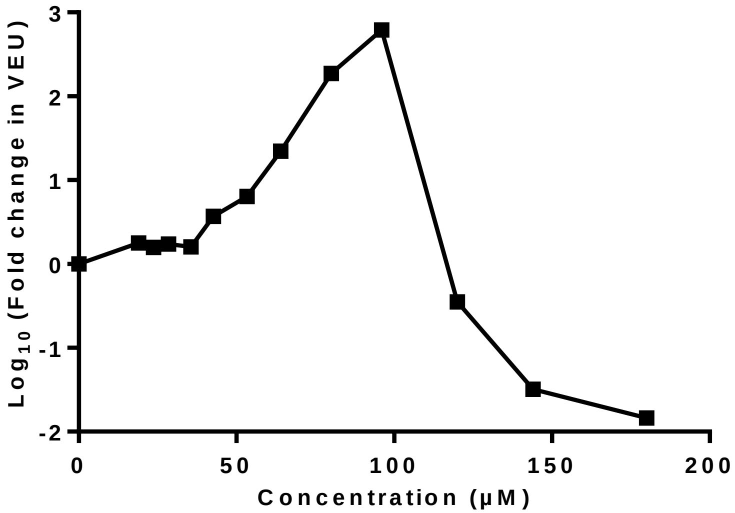

Compound ID: 23

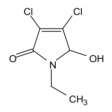

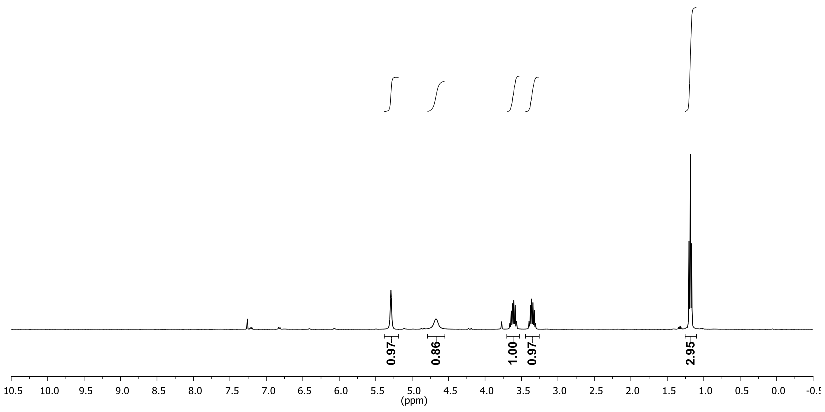

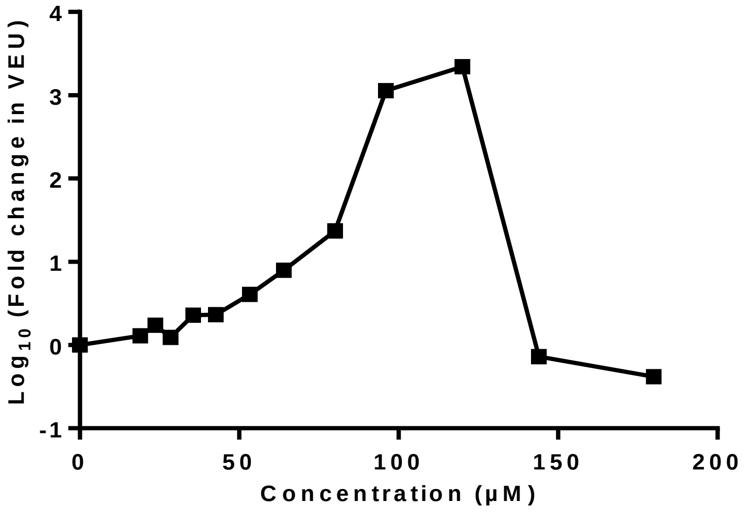

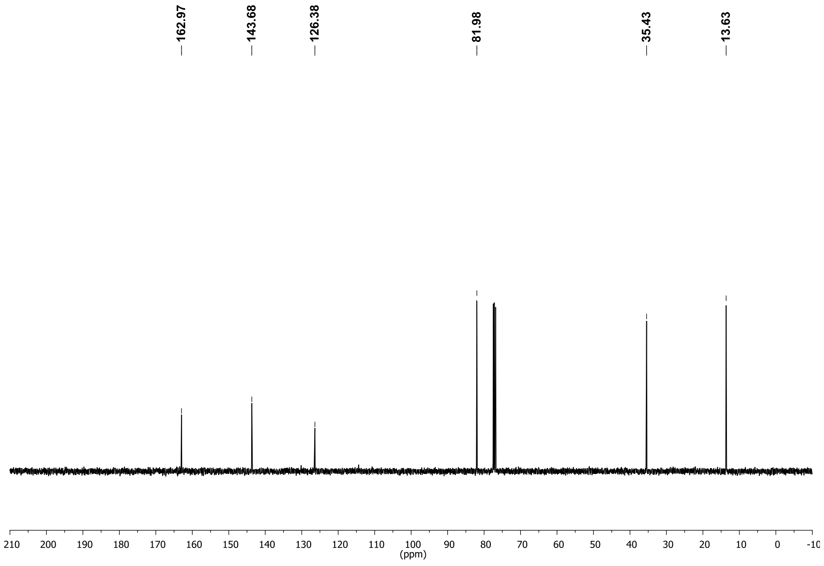

Compound ID: 24

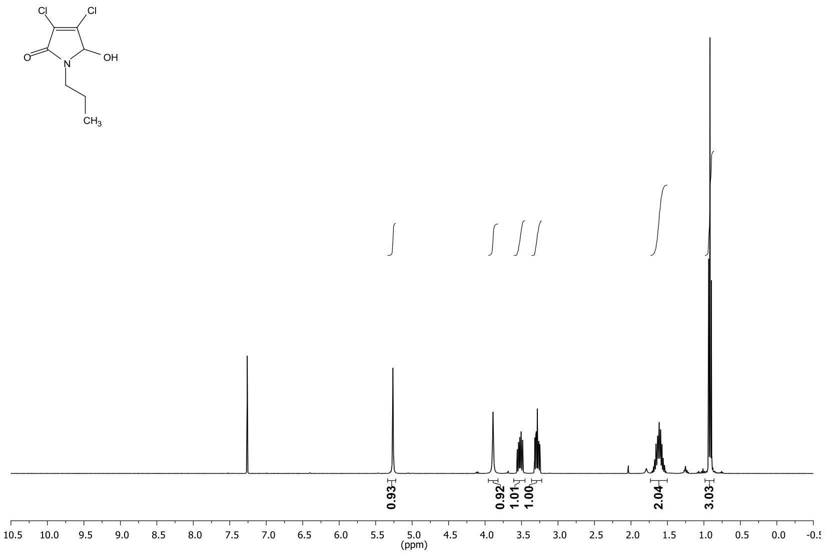

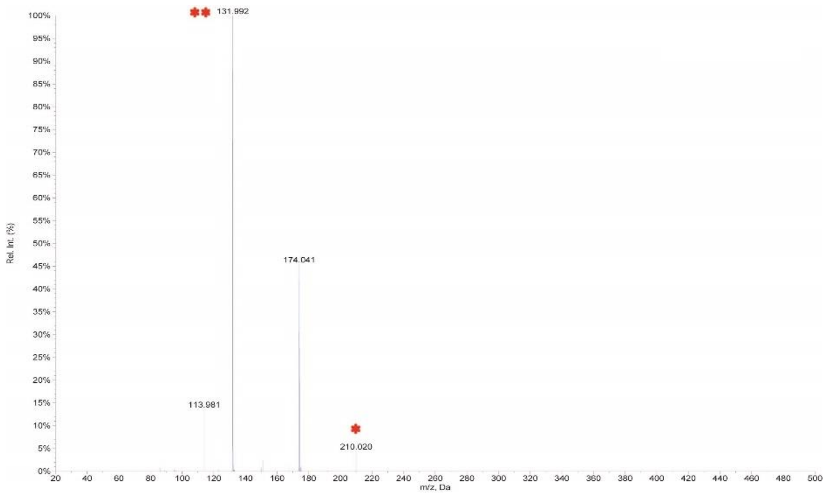

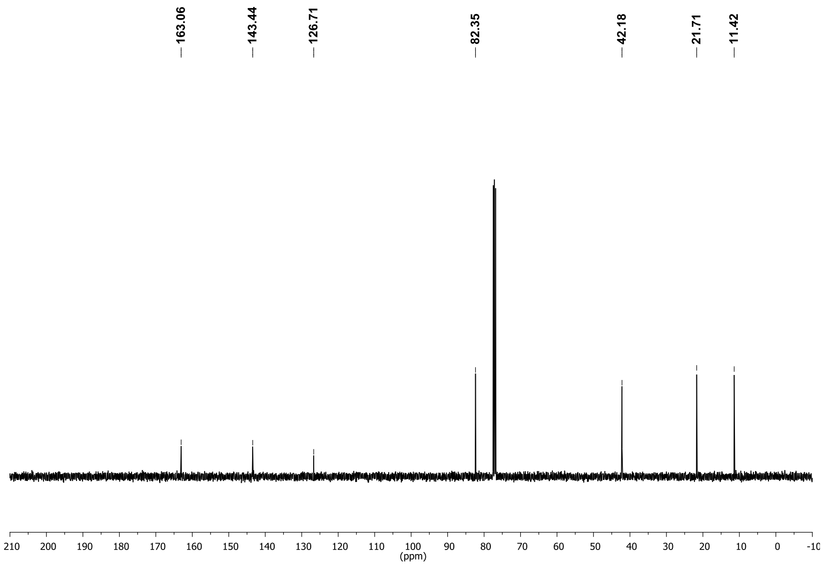

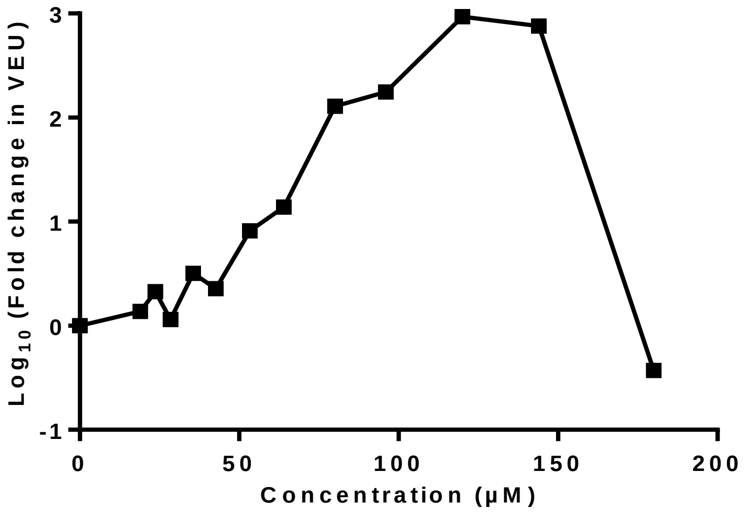

Compound ID: 25

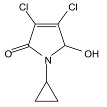

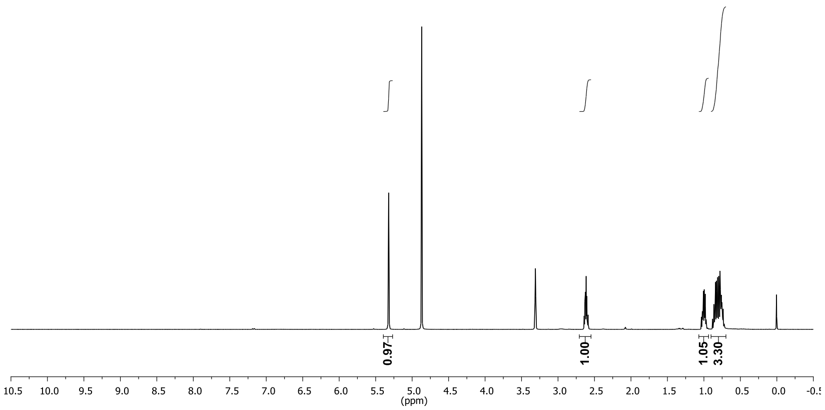

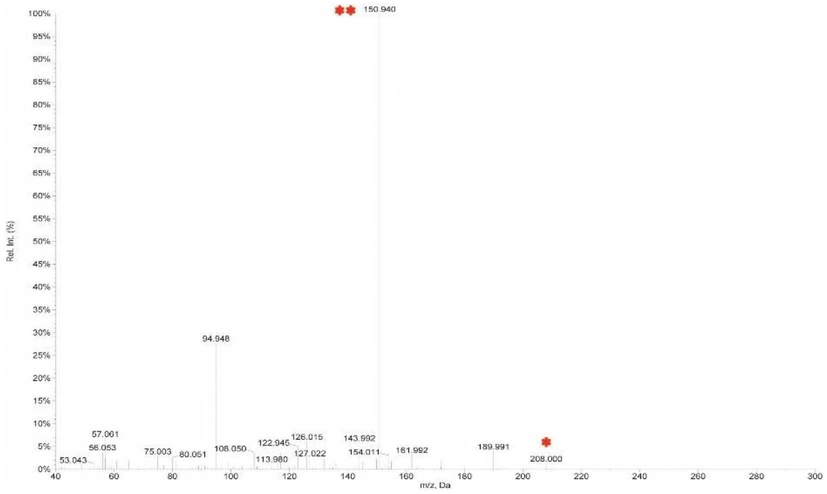

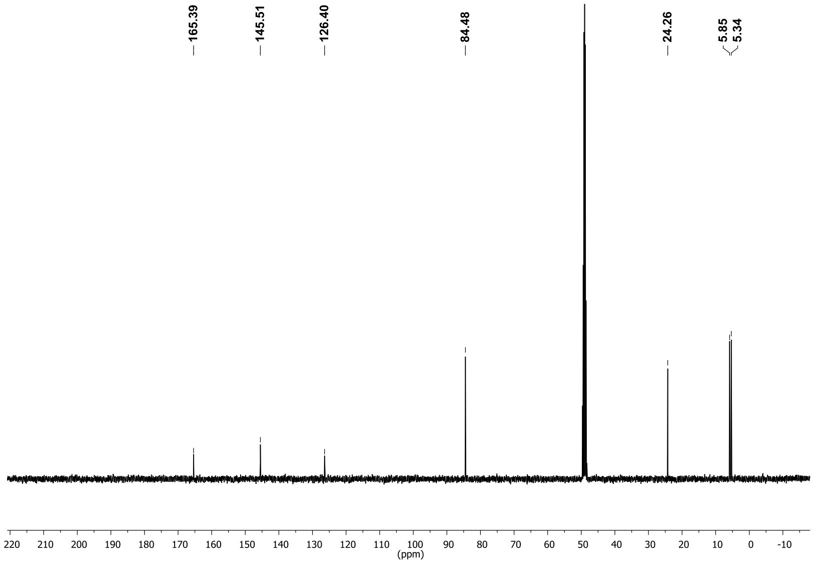

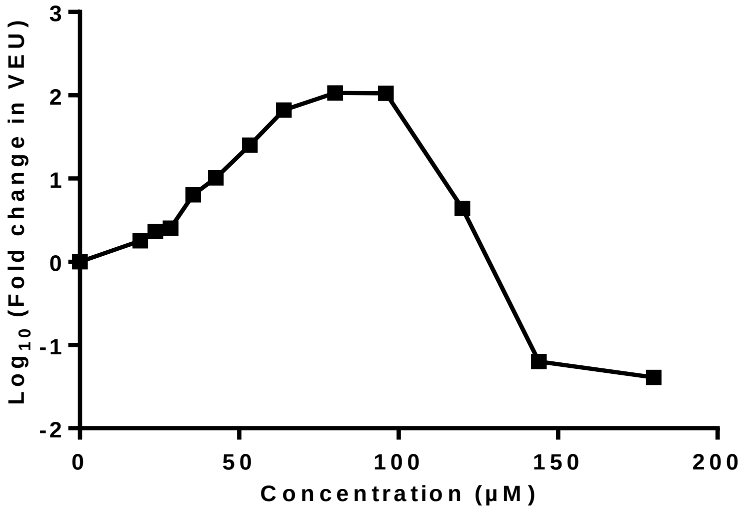

Compound ID: 26

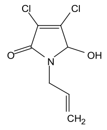

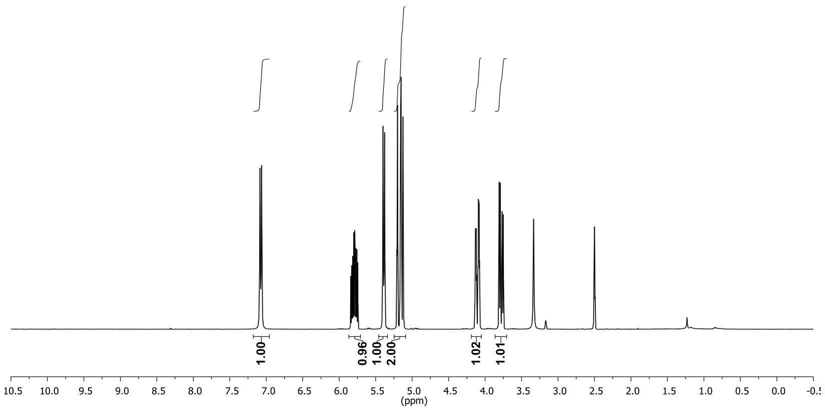

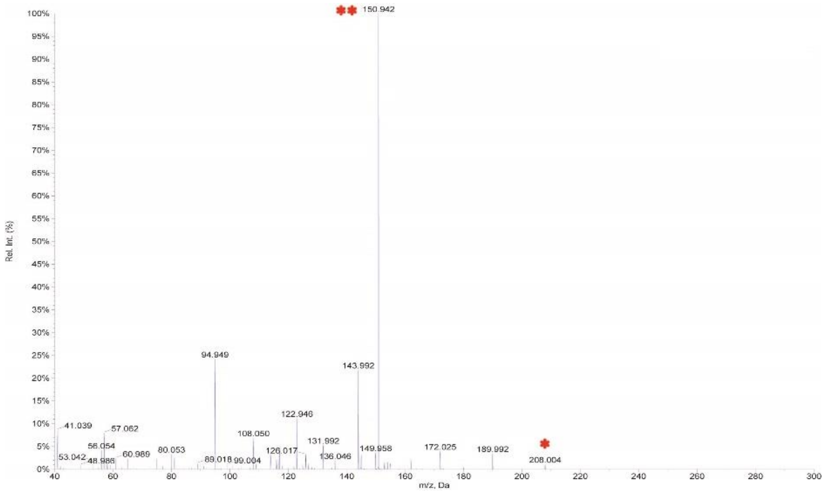

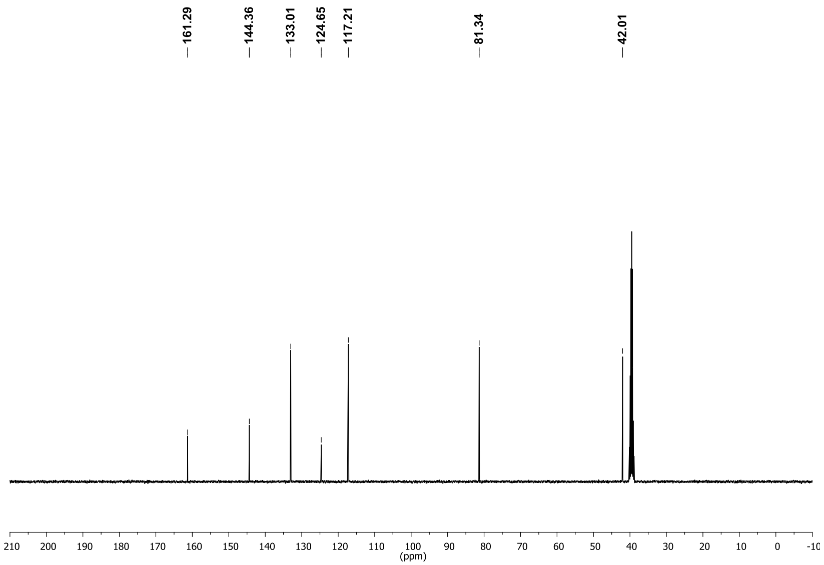

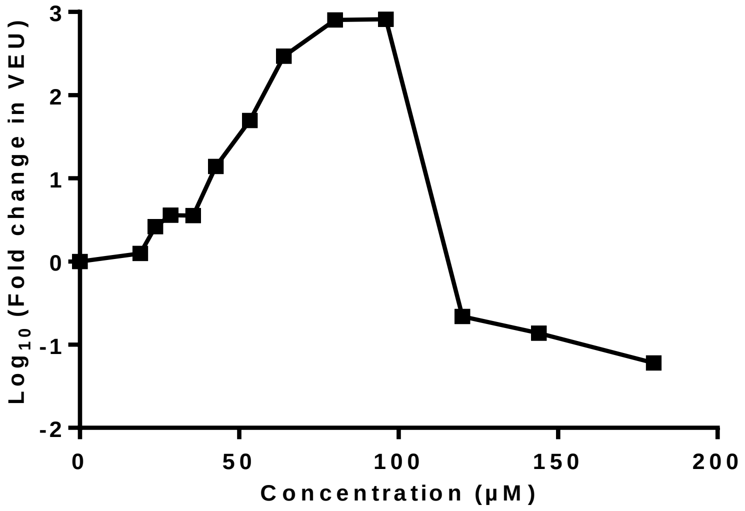

Compound ID: 27

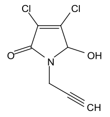

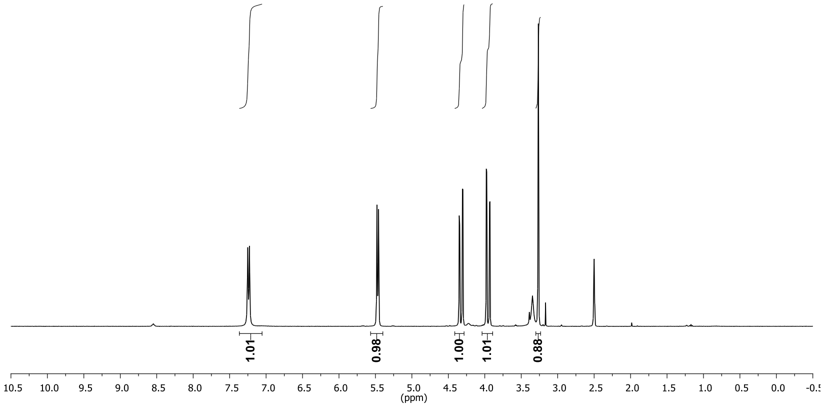

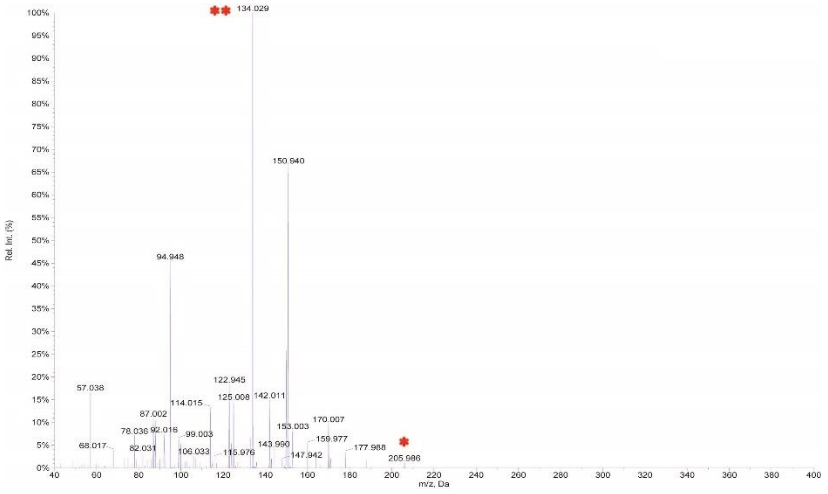

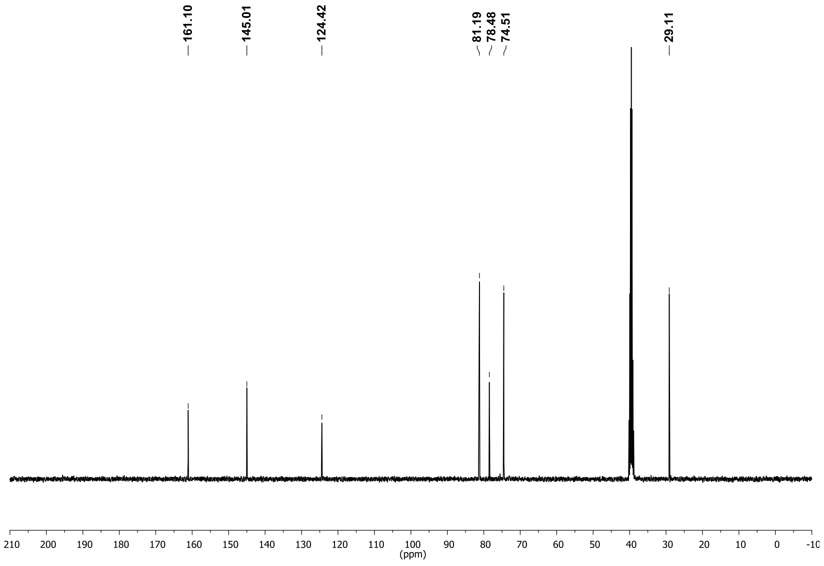

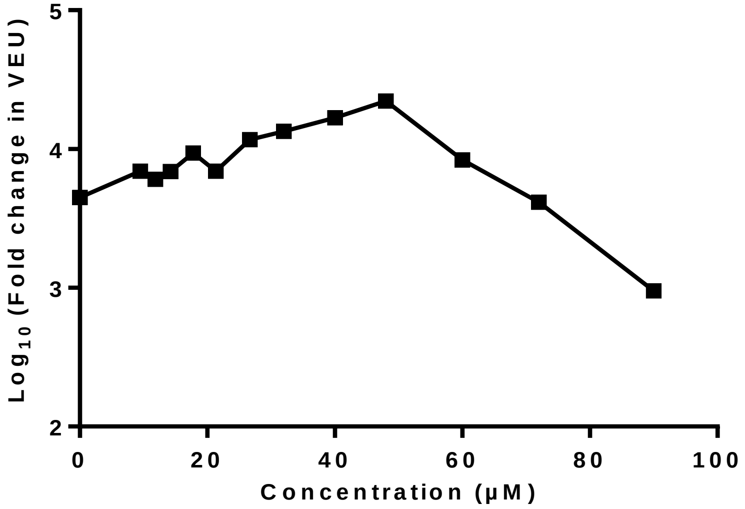

Compound ID: 28

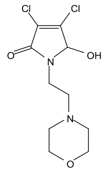

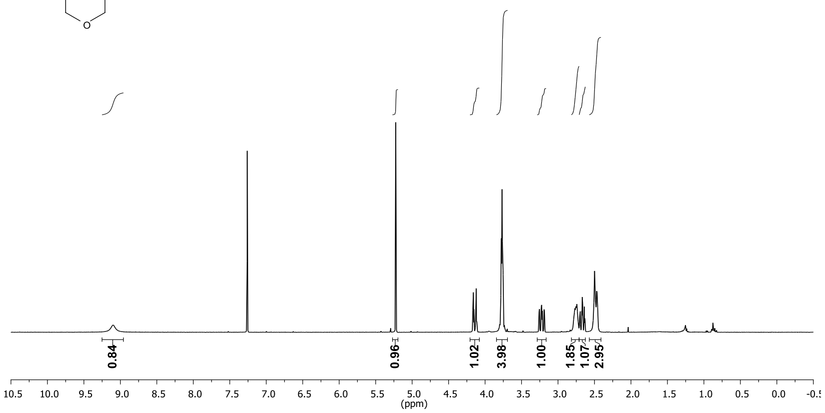

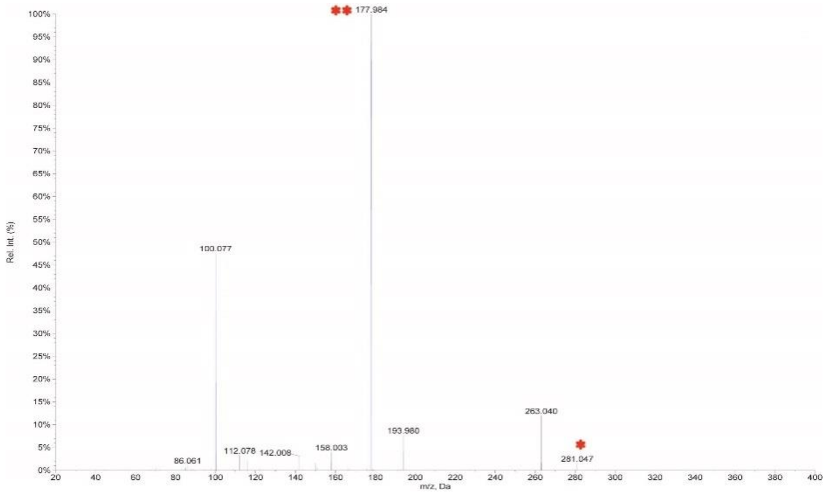

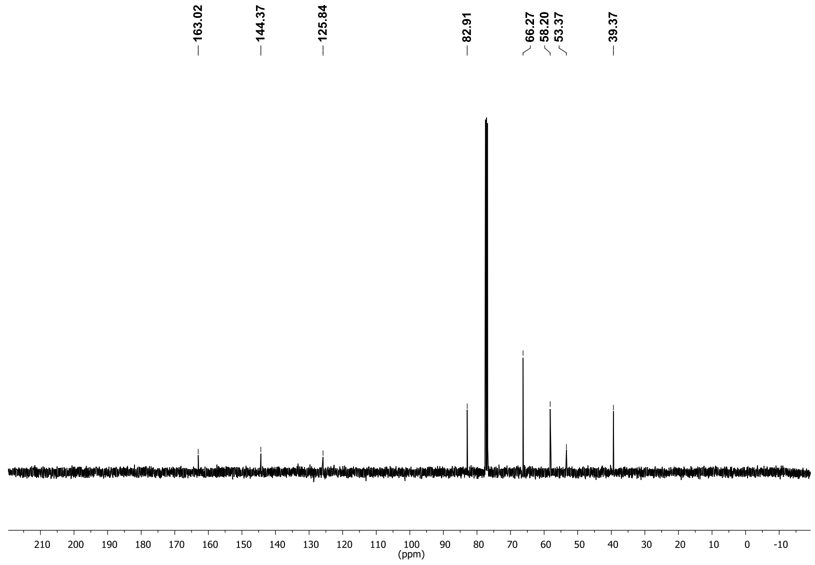

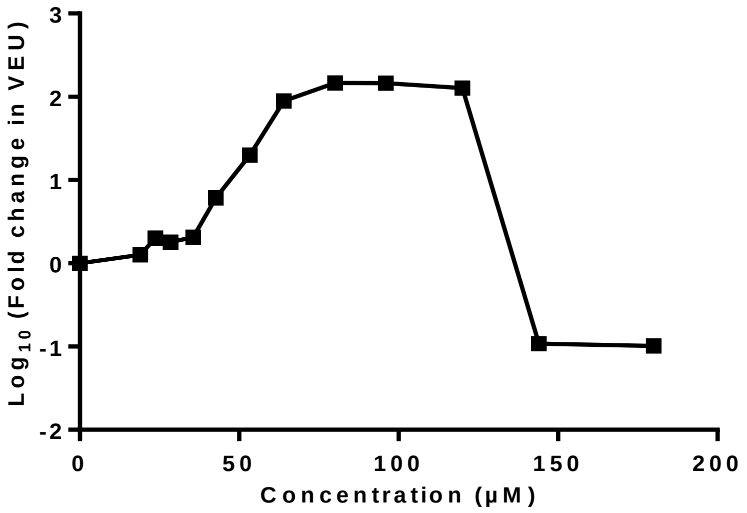

Compound ID: 29

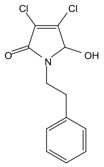

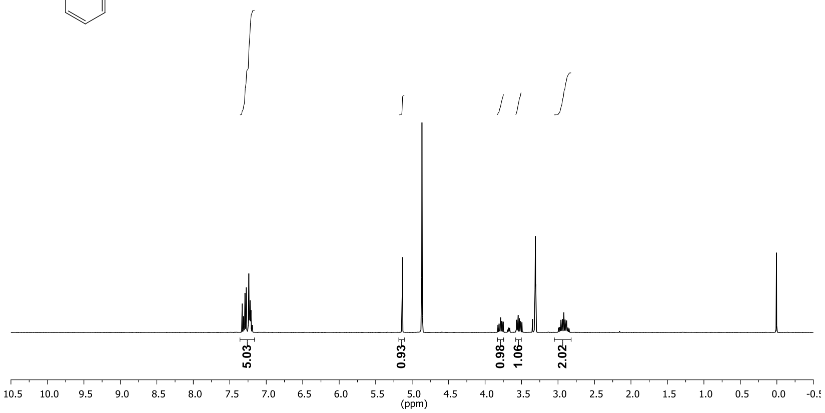

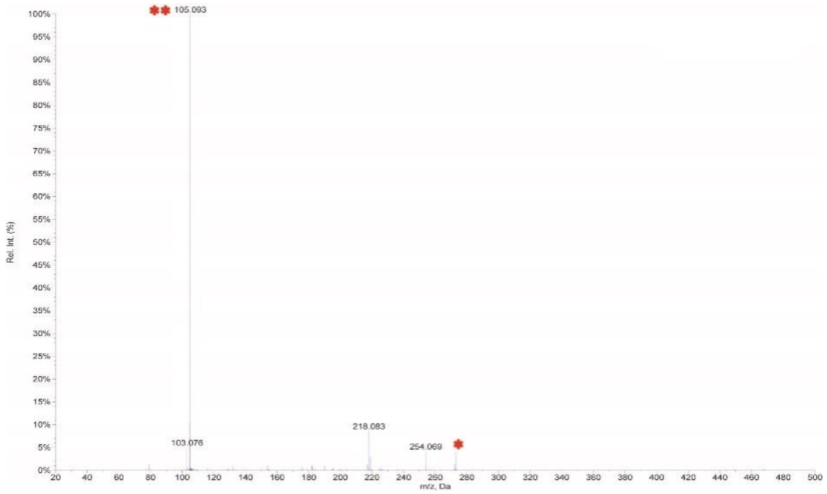

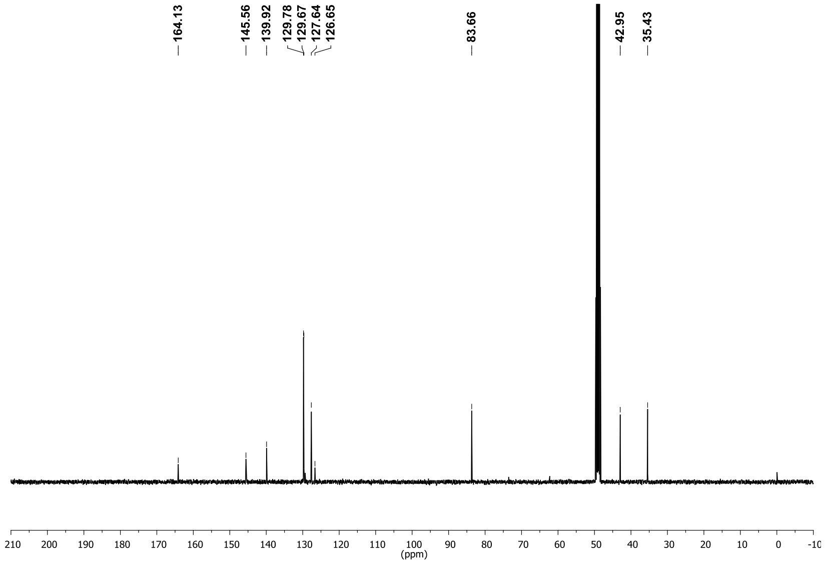

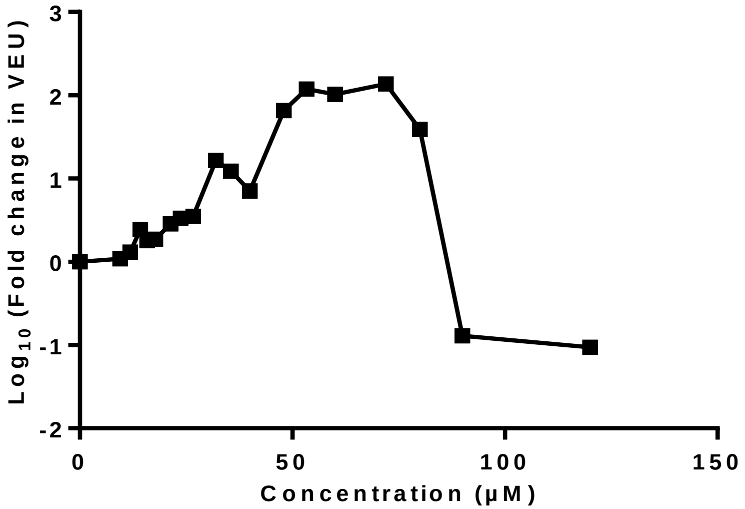

Compound ID: 30

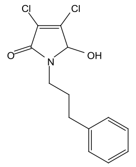

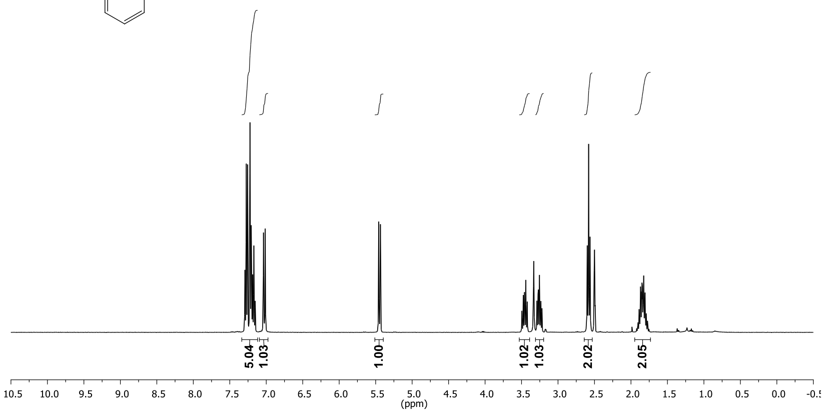

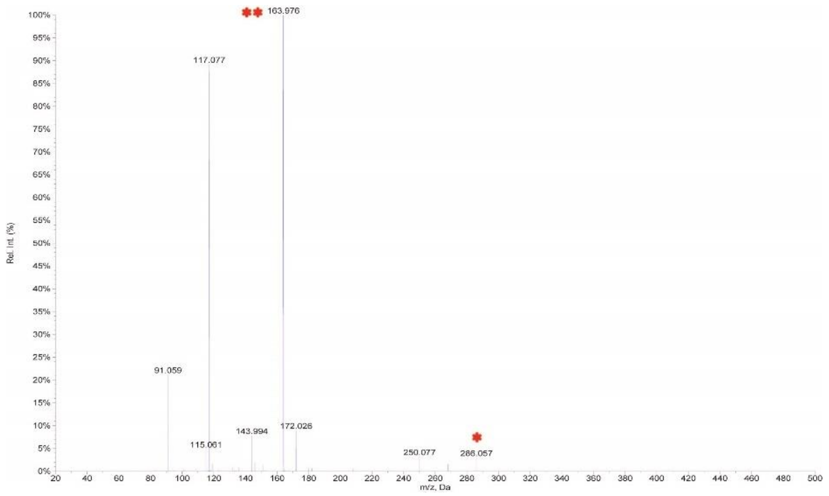

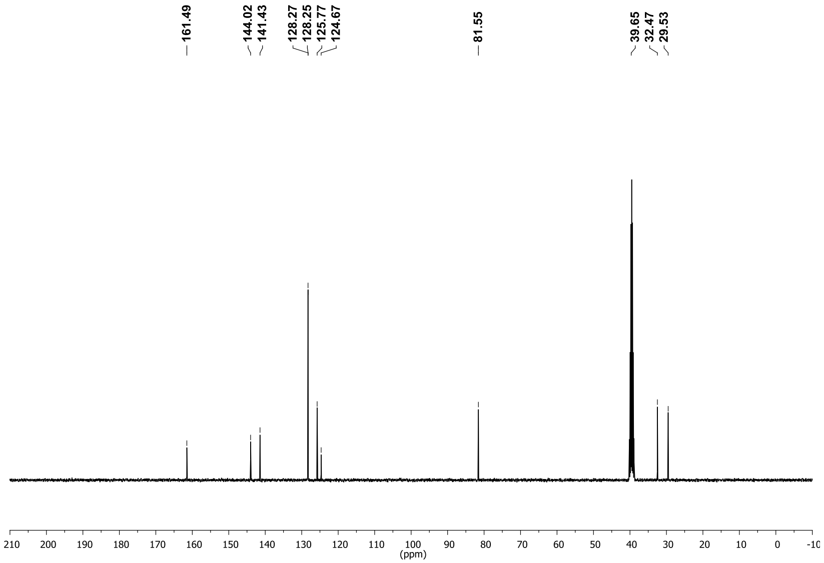

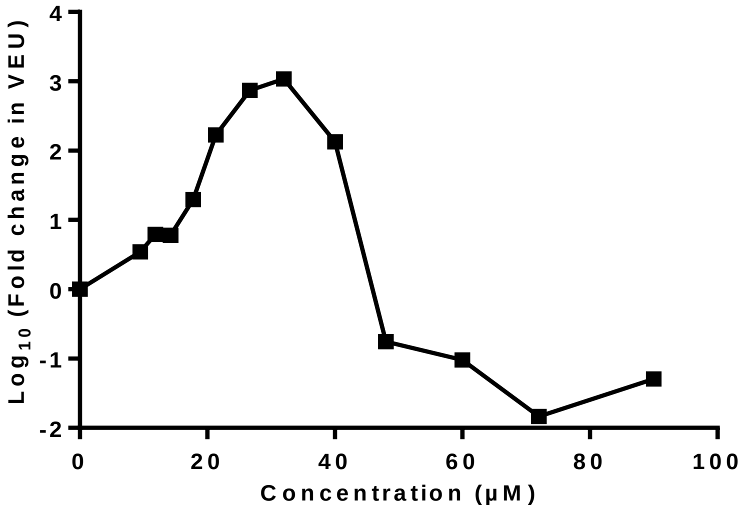

Compound ID: 31

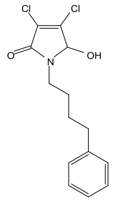

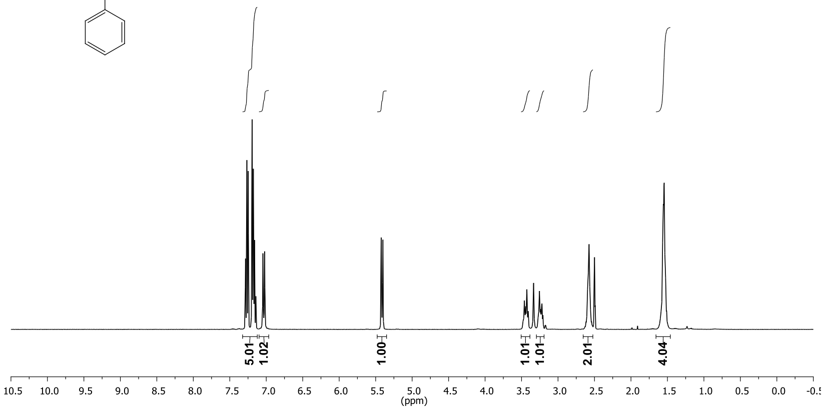

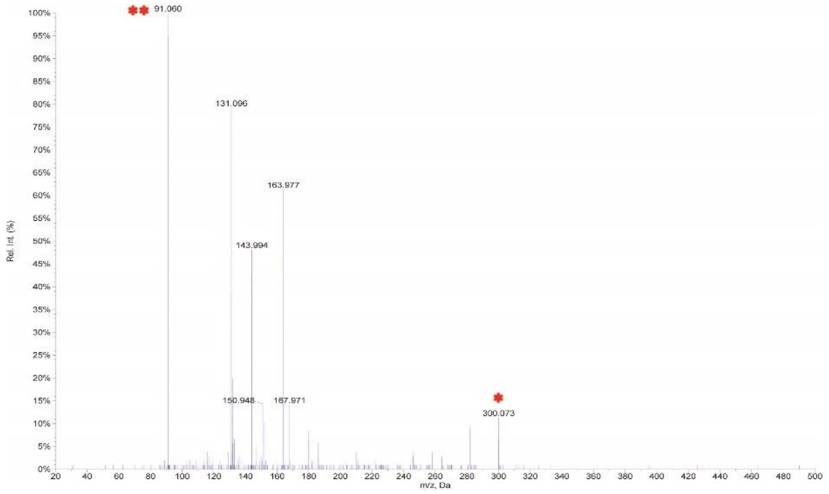

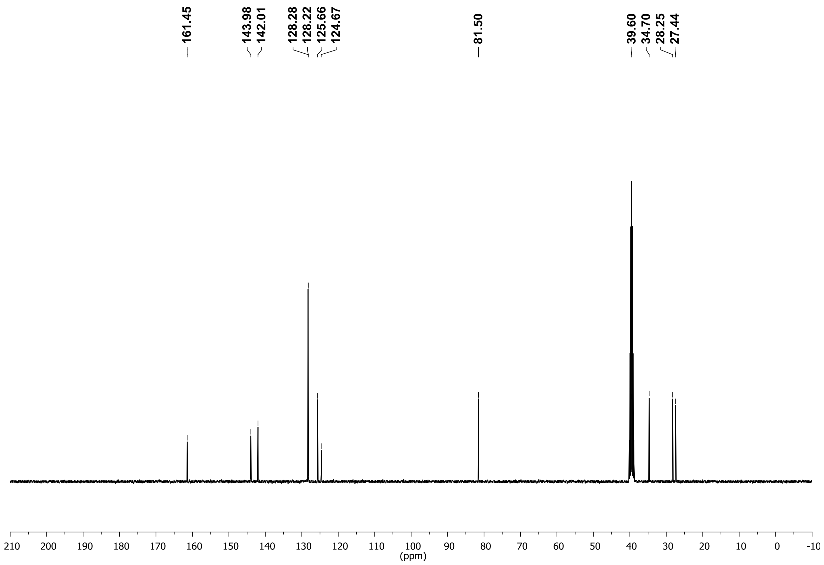

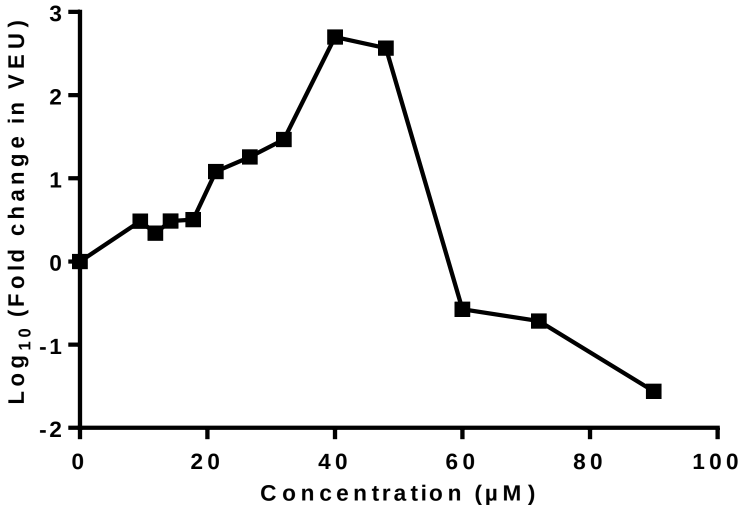

Compound ID: 32

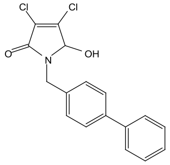

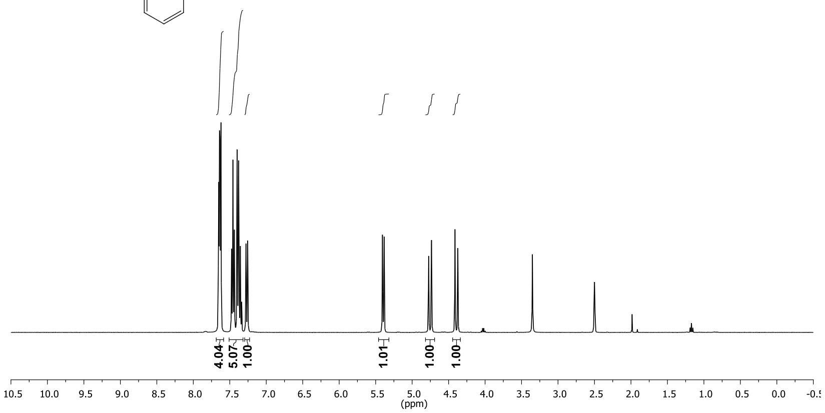

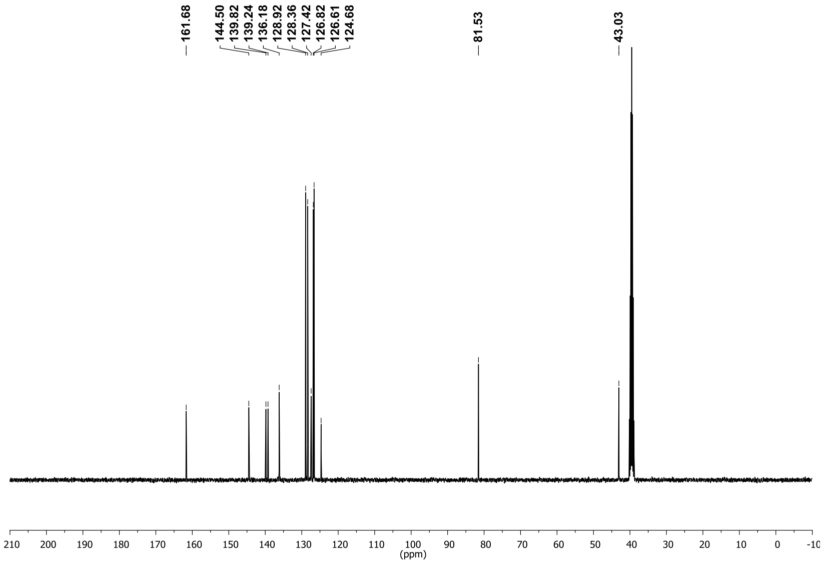

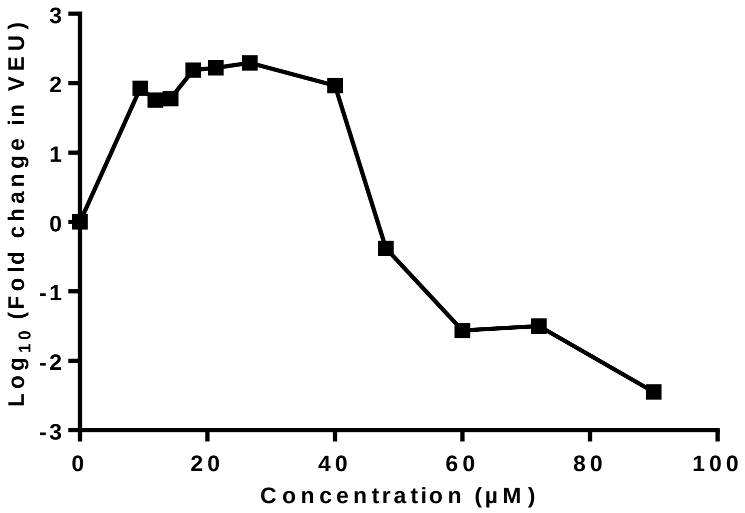

Compound ID: 33

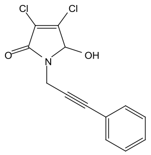

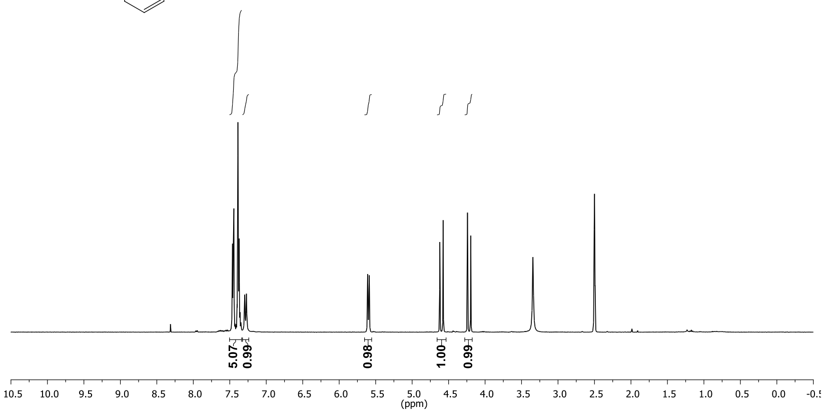

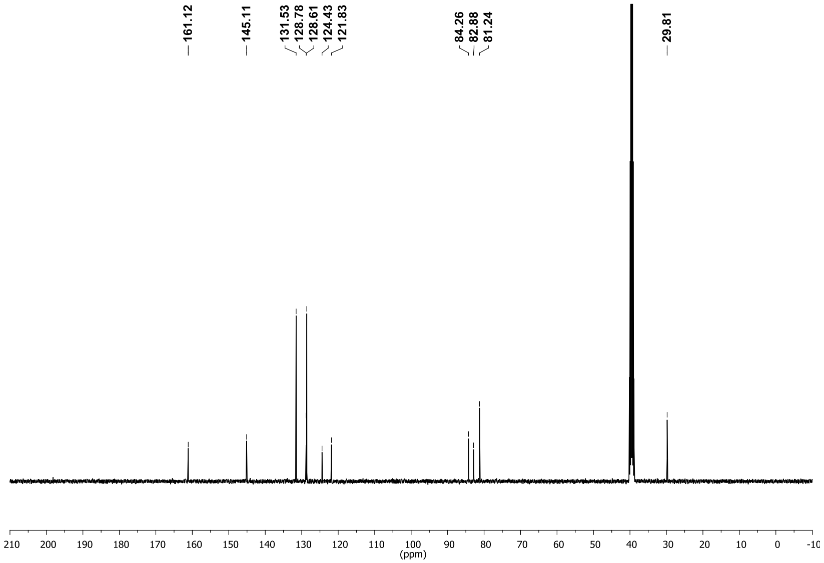

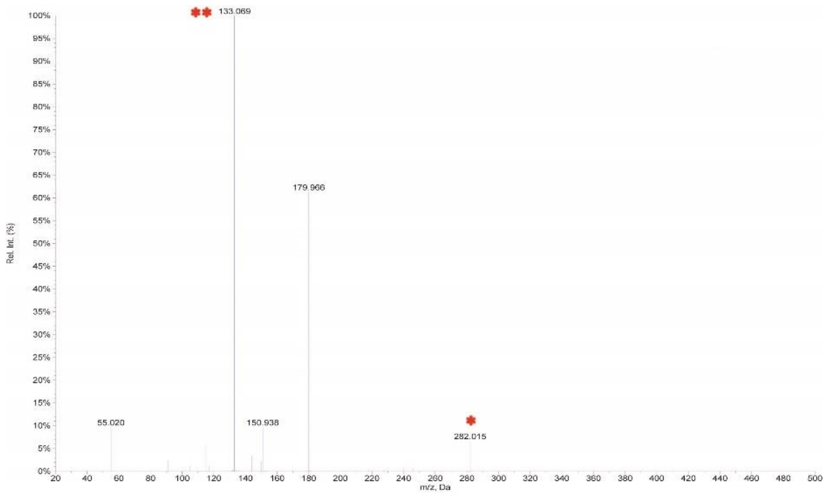

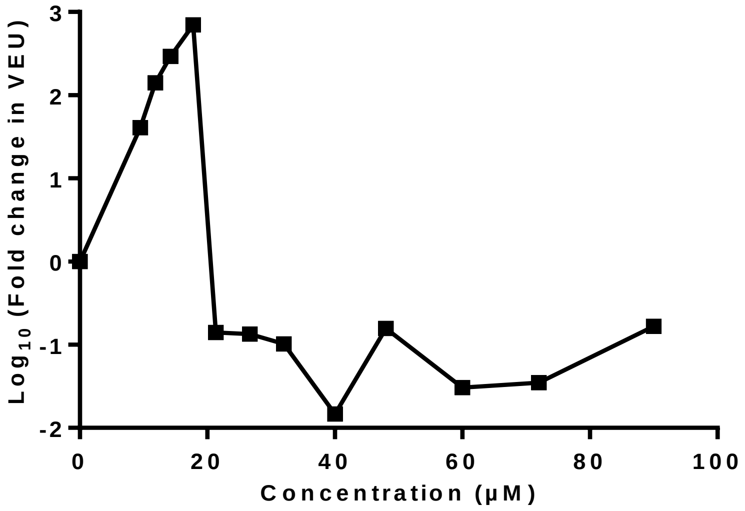

Compound ID: 34

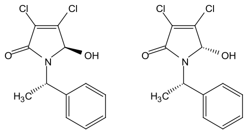

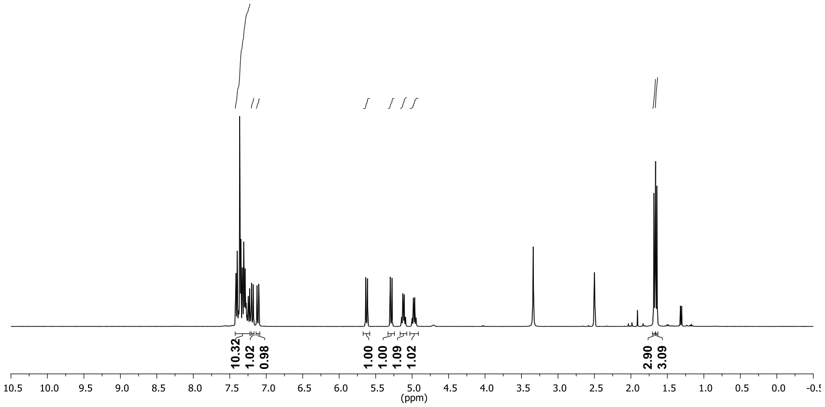

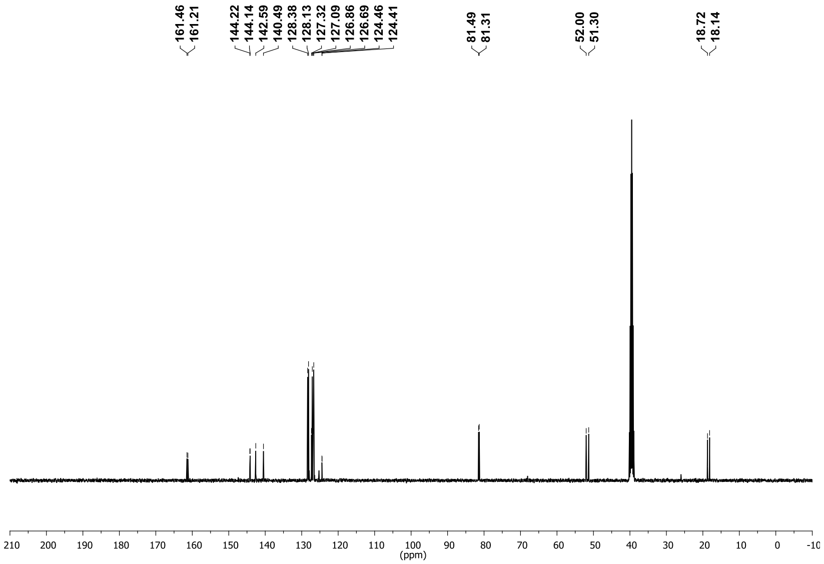

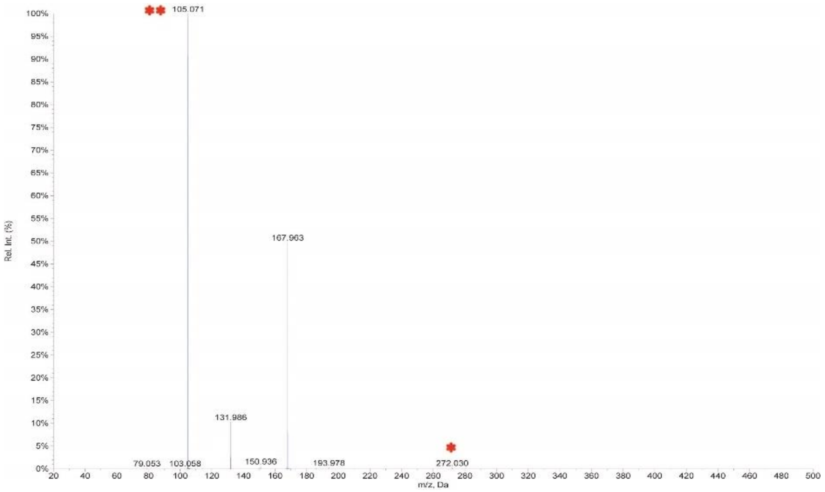

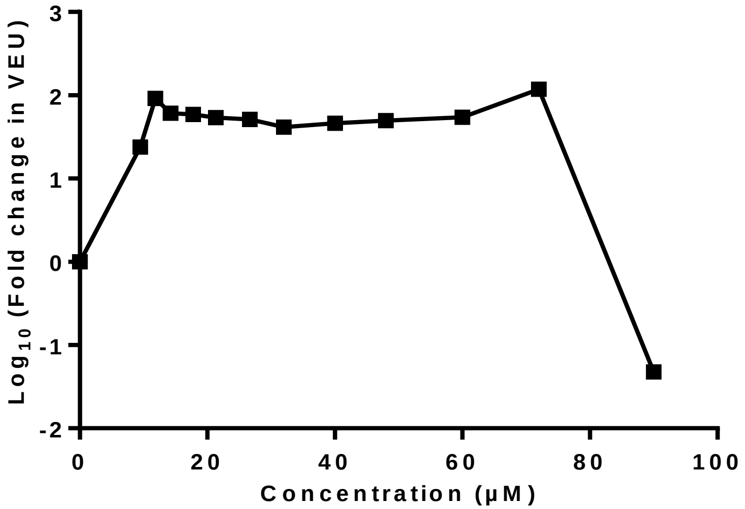

Compound ID: 35

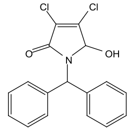

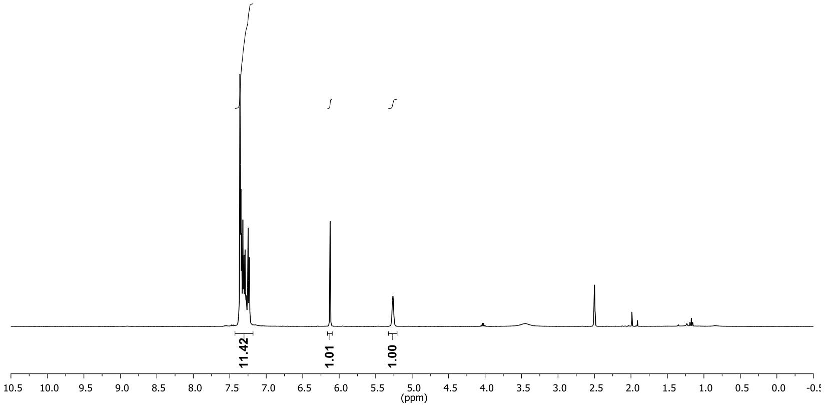

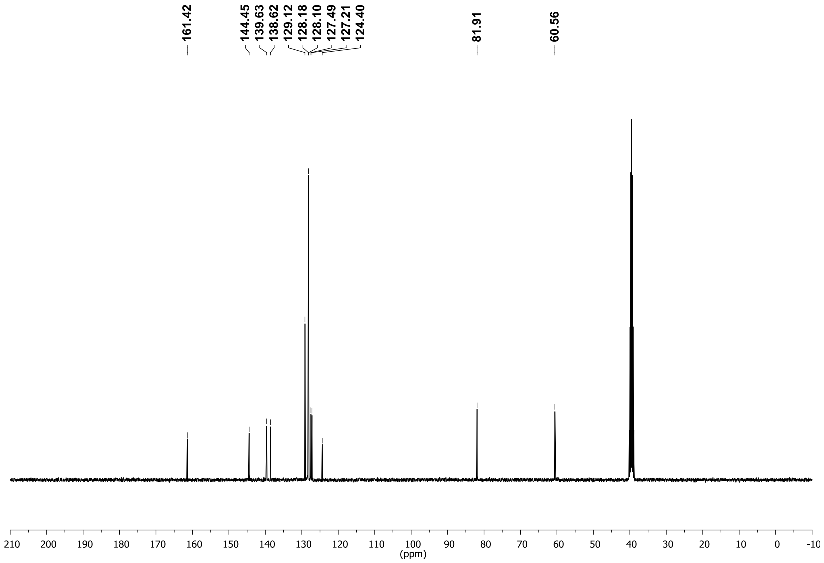

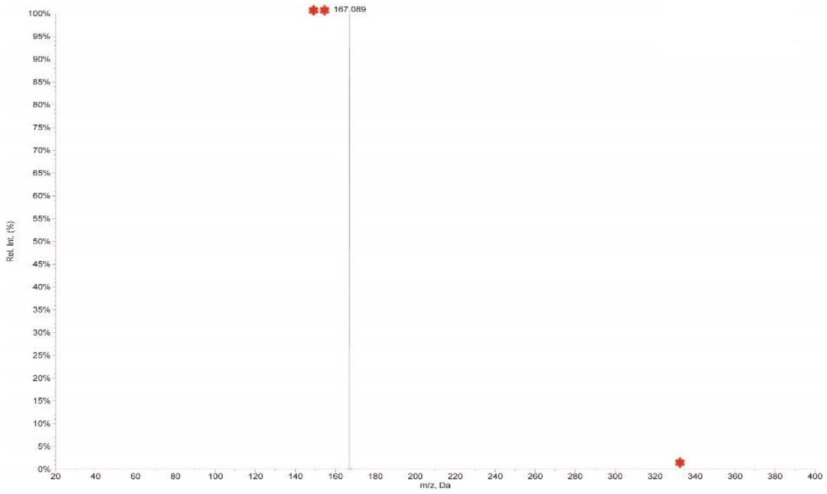

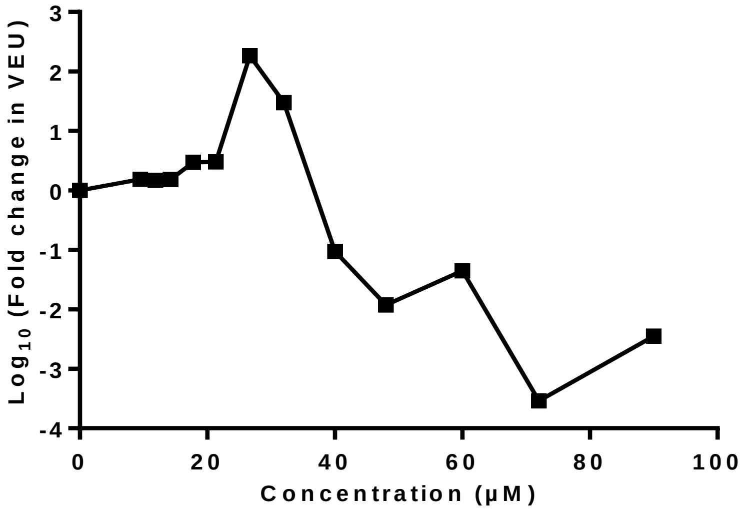

Compound ID: 36

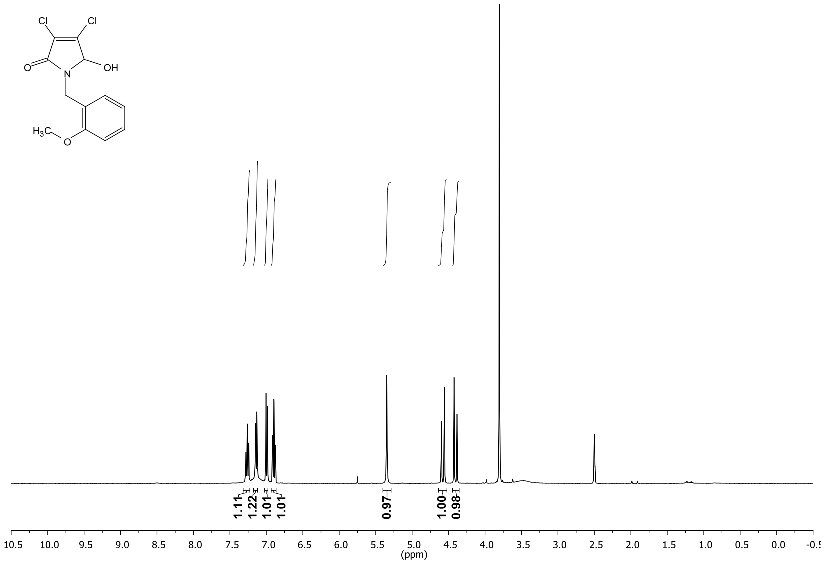

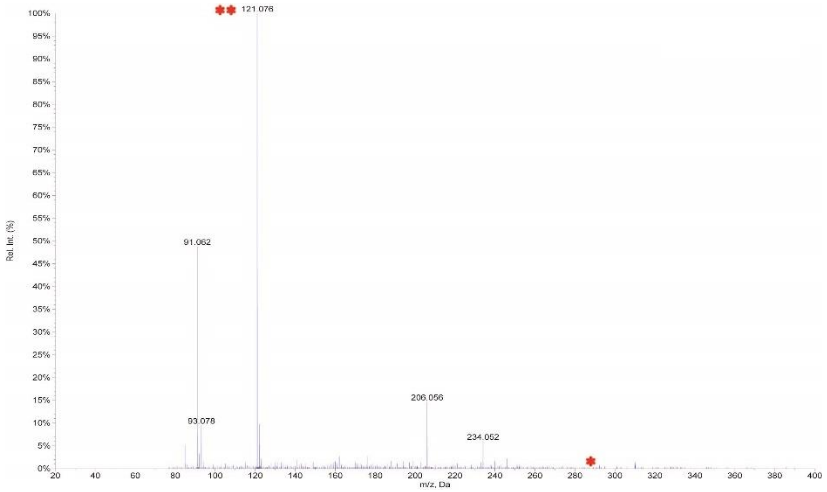

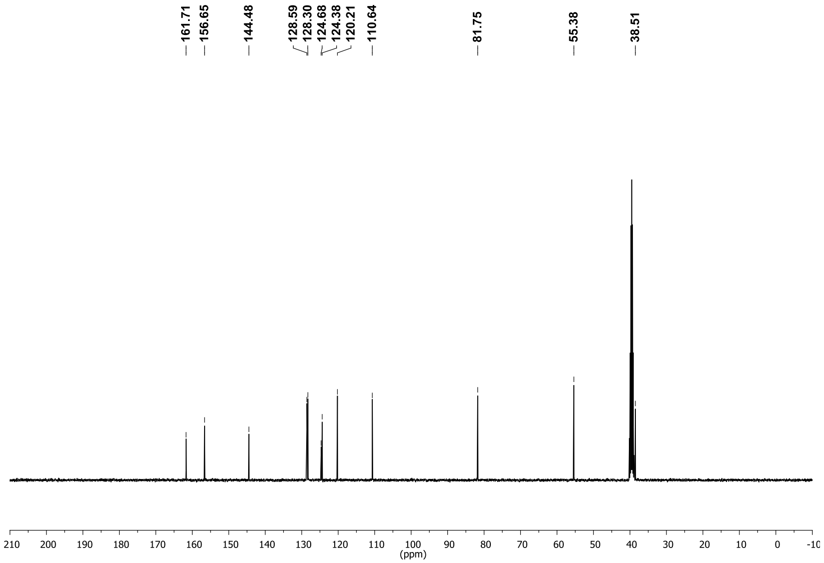

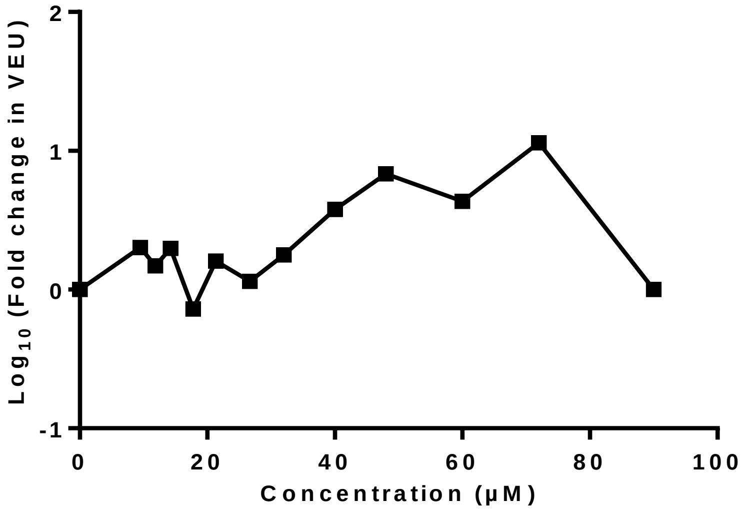

Compound ID: 37

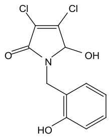

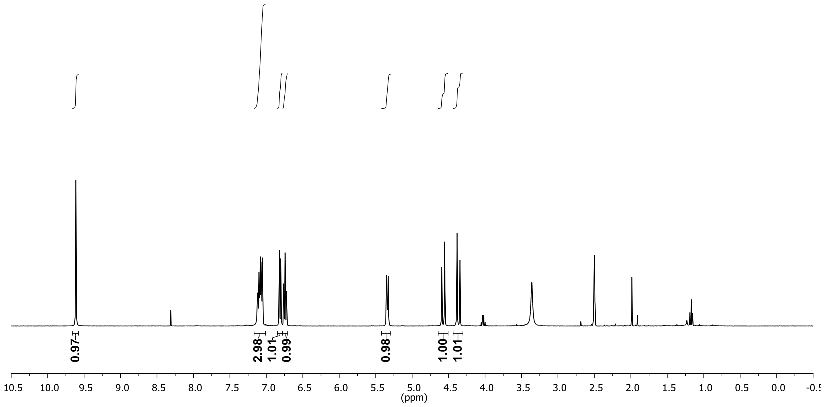

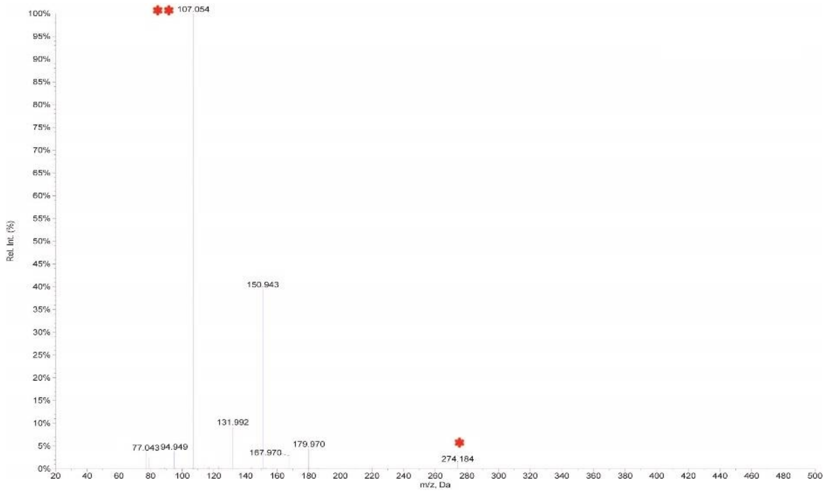

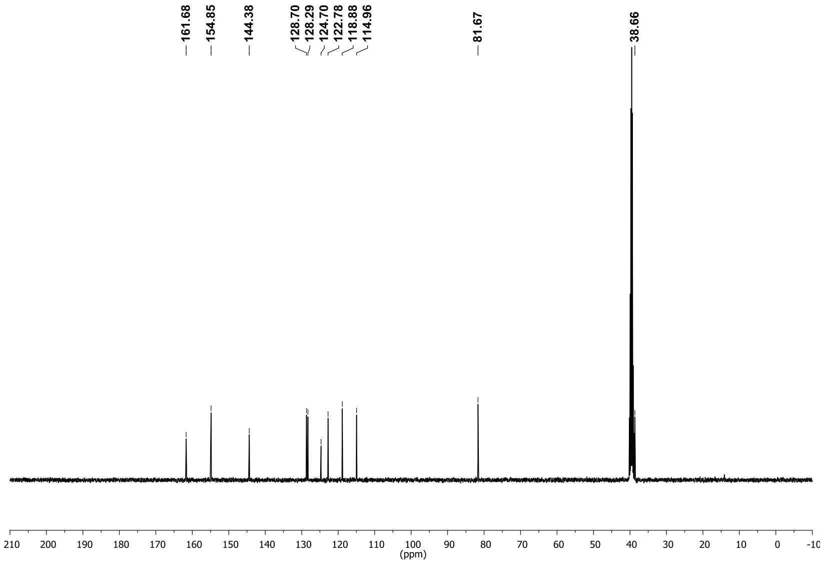

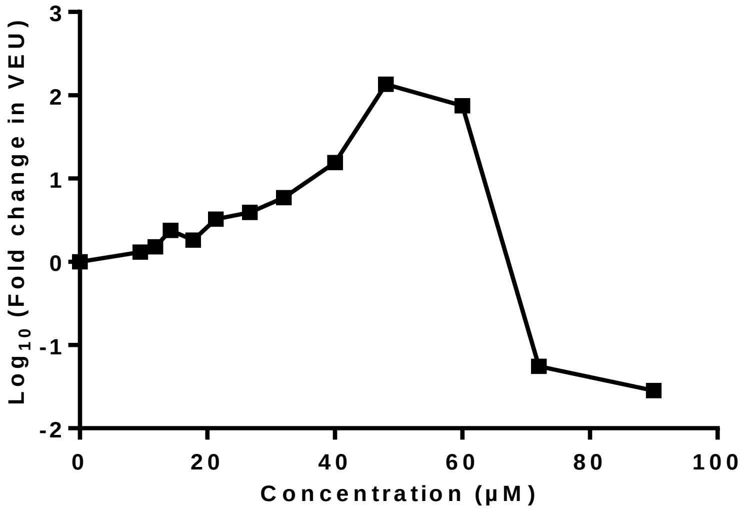

Compound ID: 38

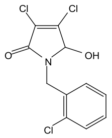

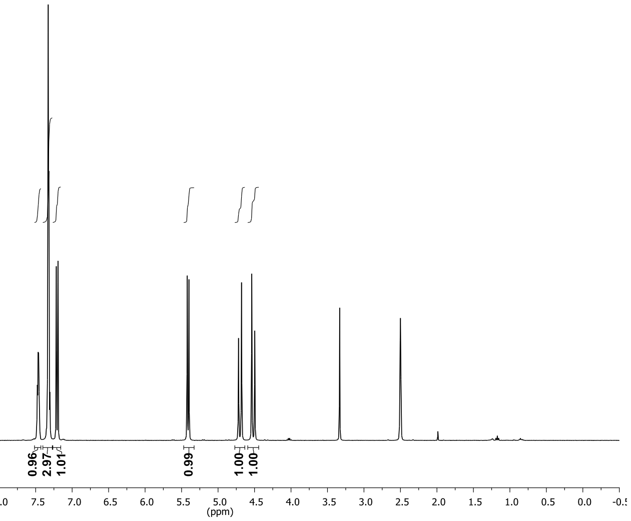

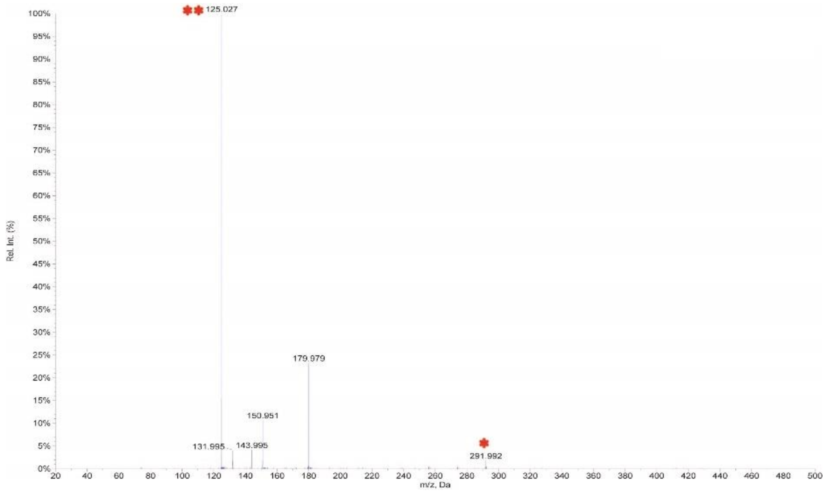

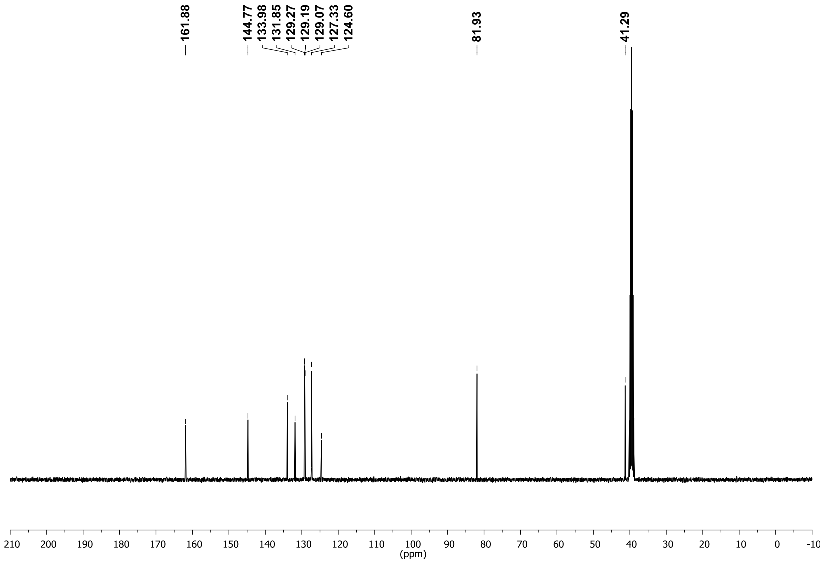

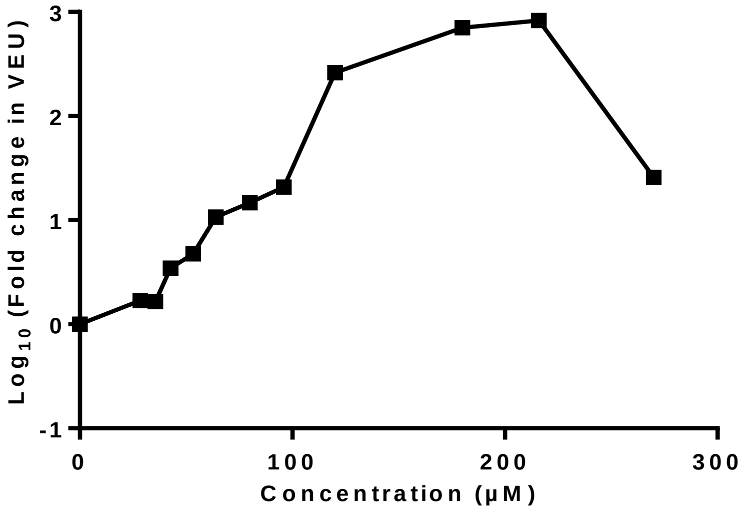

Compound ID: 39

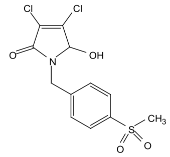

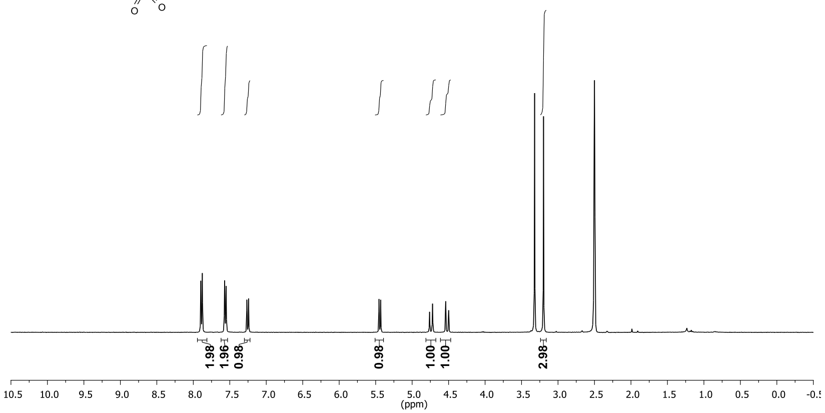

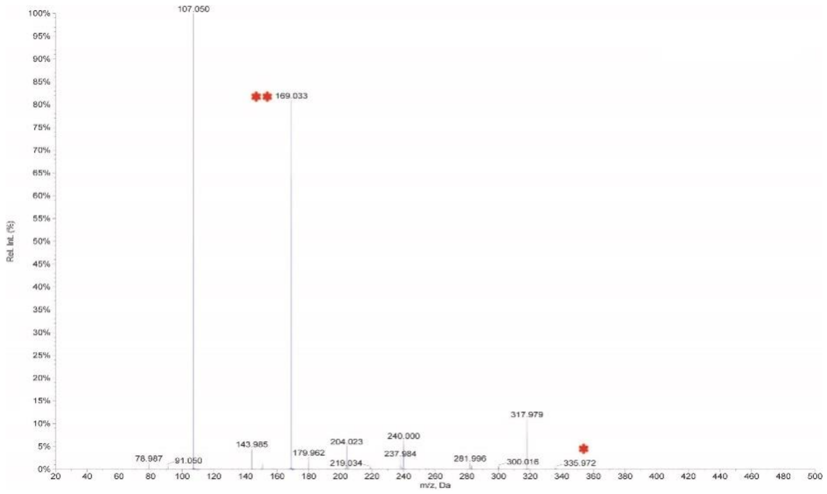

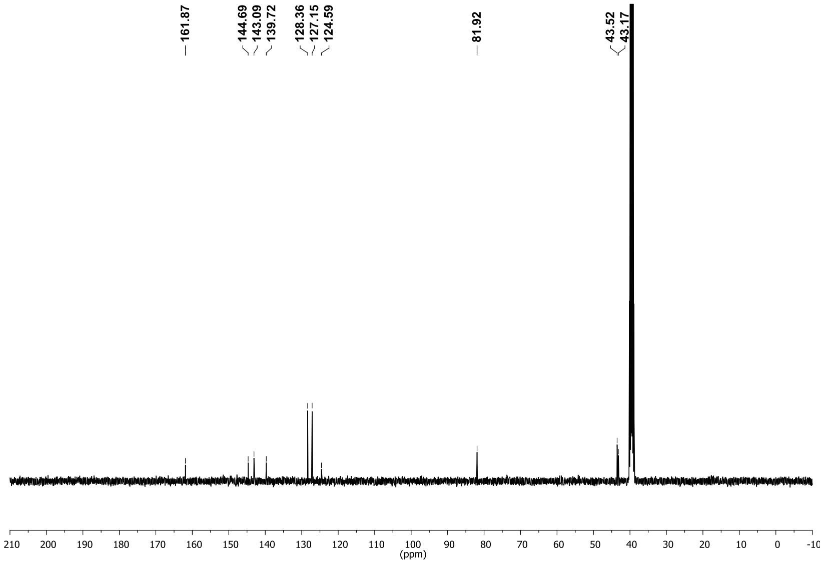

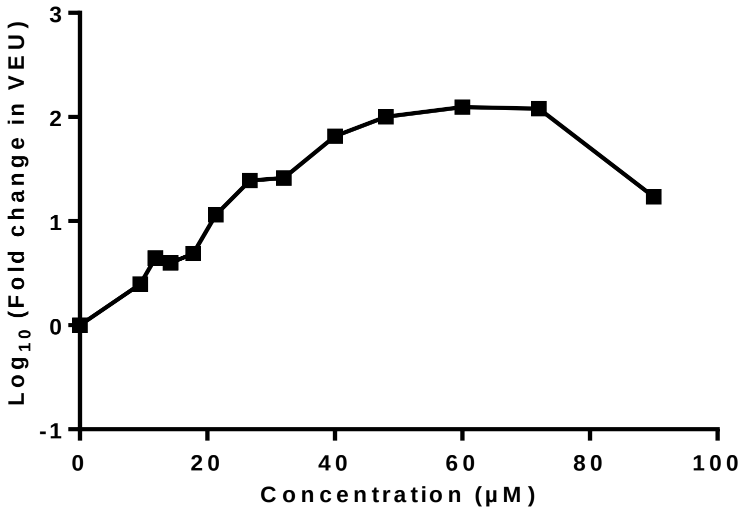

Compound ID: 40

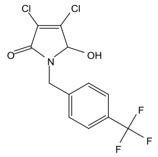

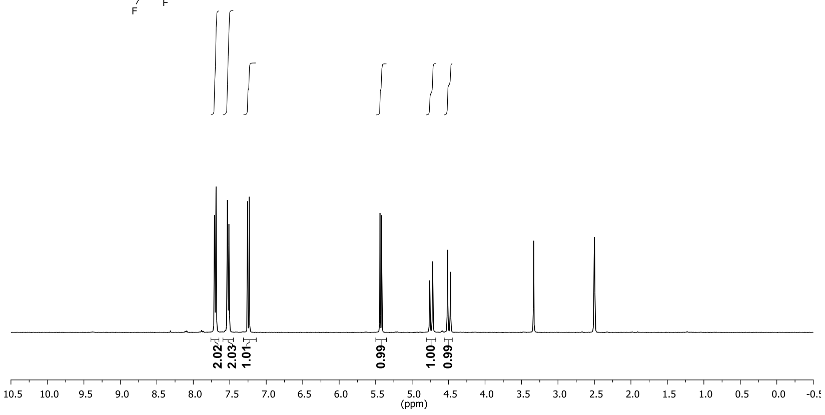

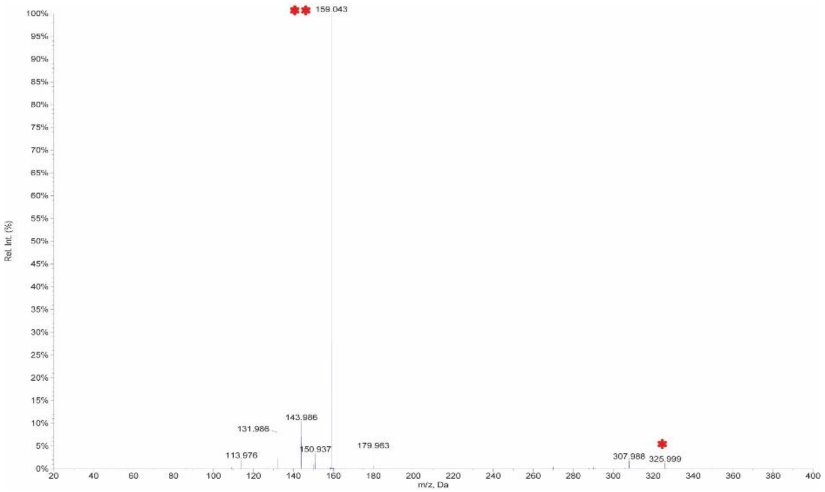

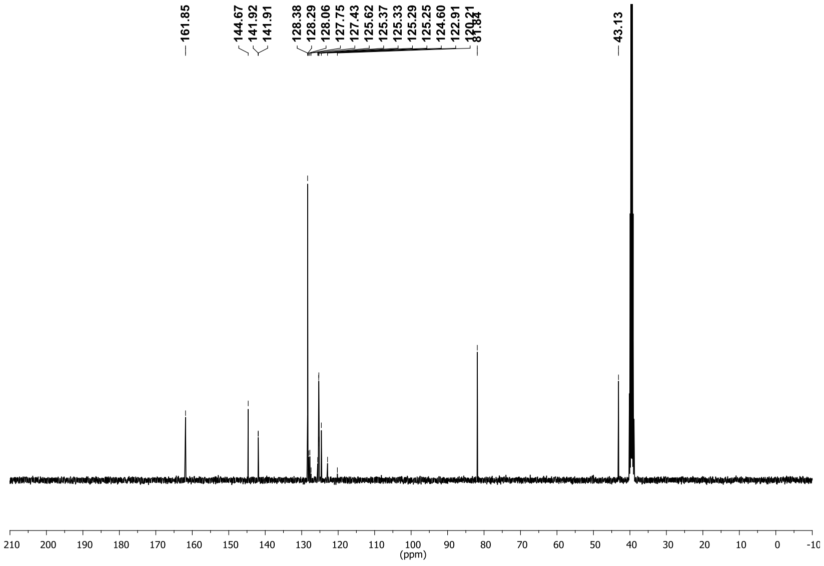

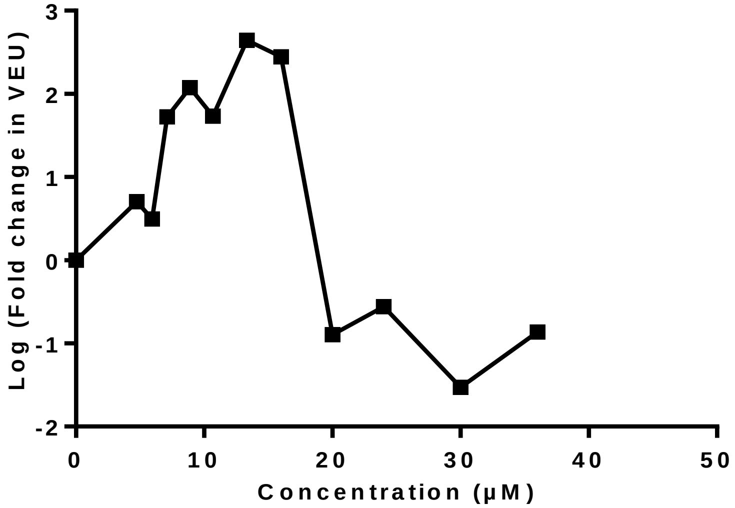

Compound ID: 41

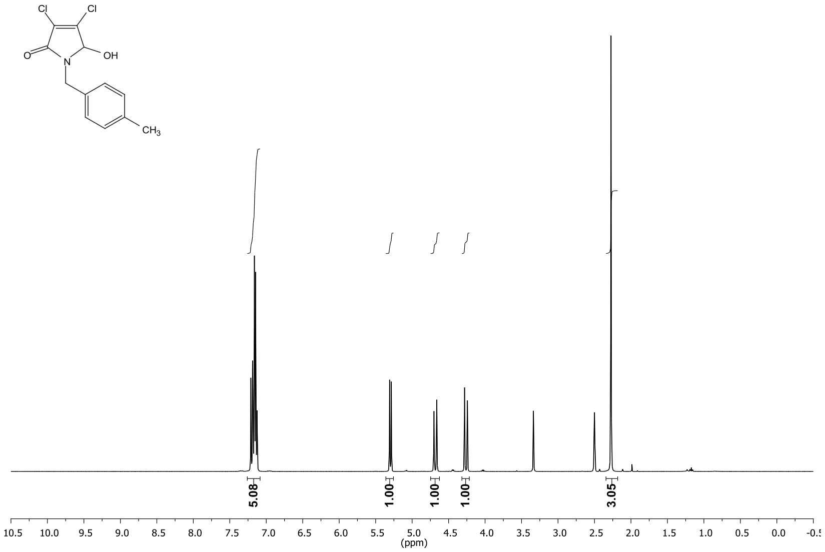

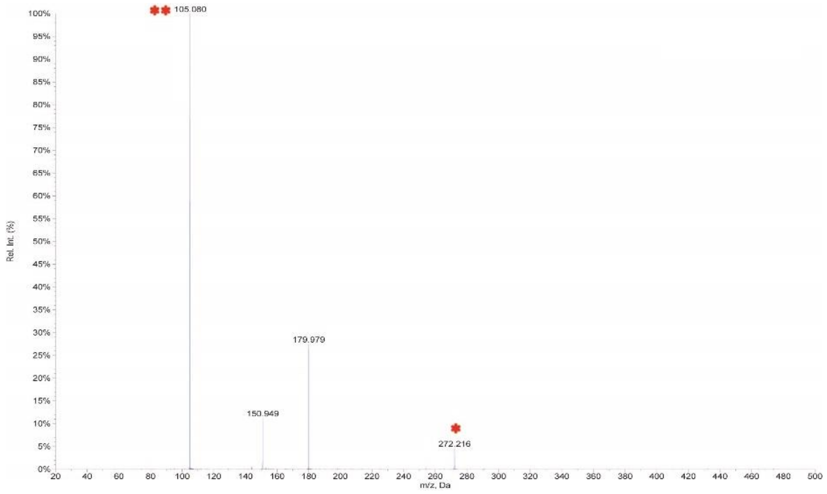

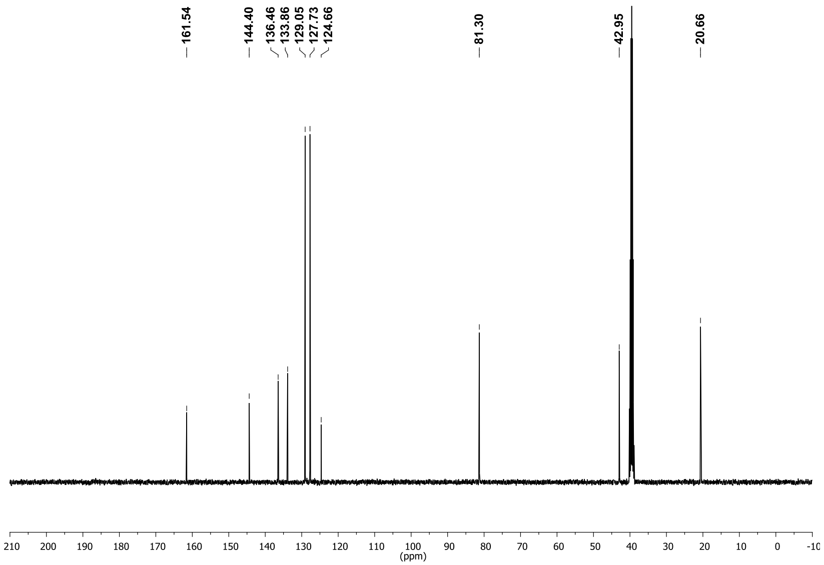

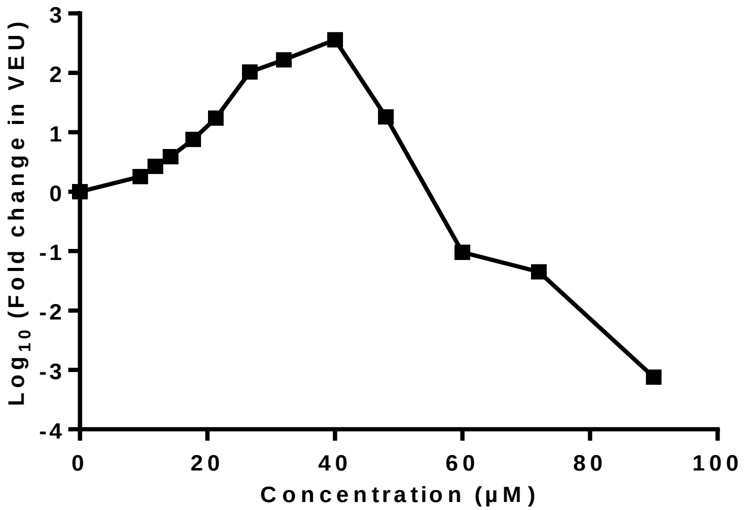

Compound ID: 42

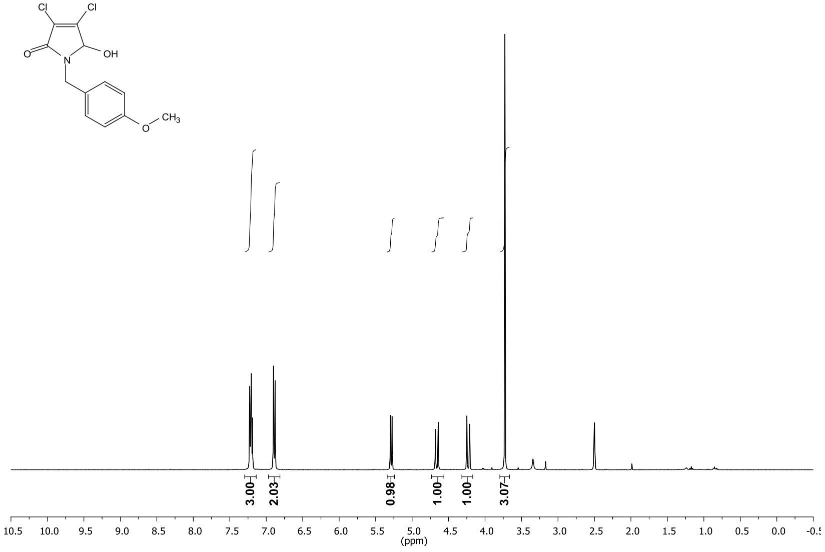

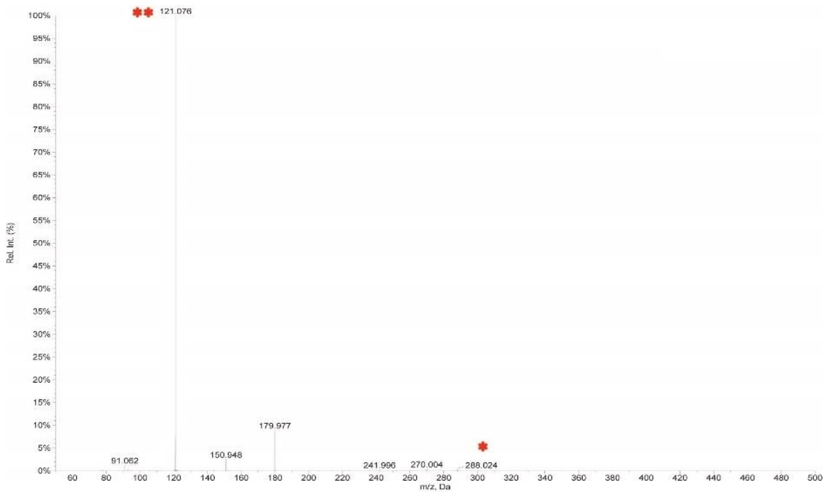

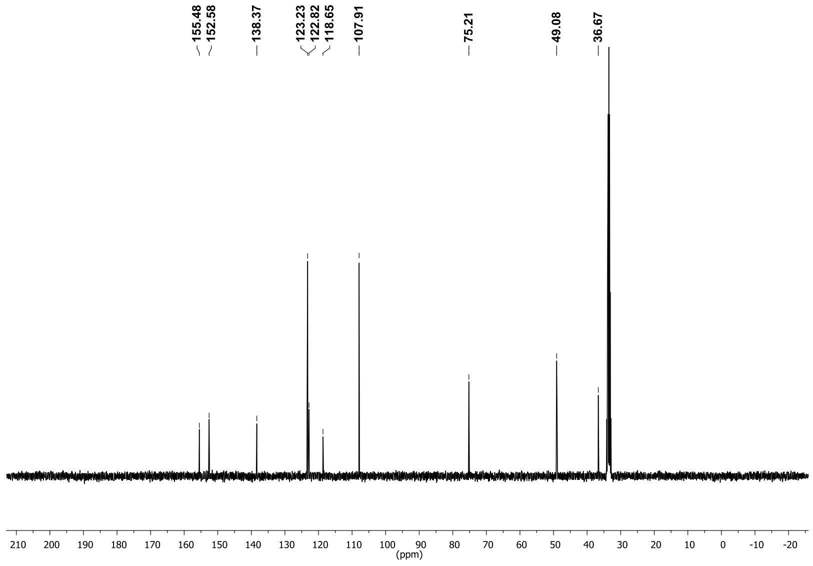

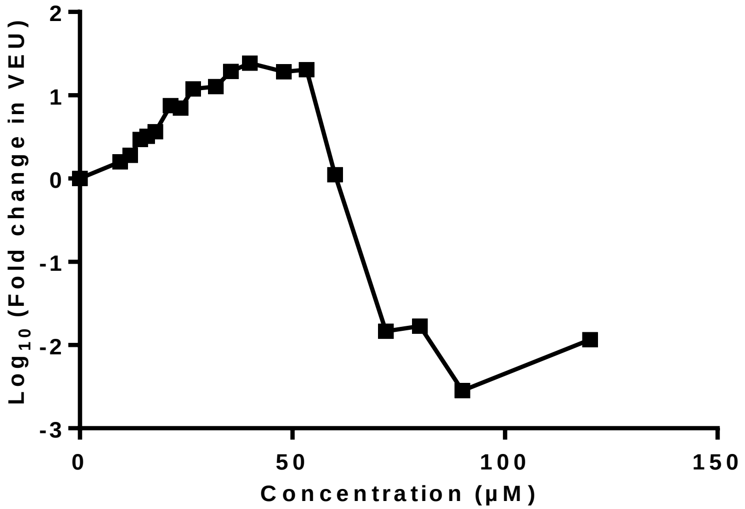

Compound ID: 43

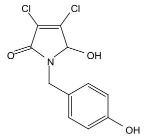

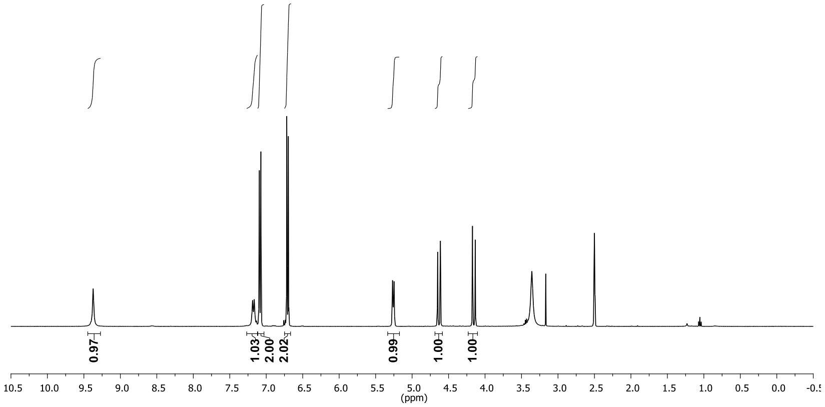

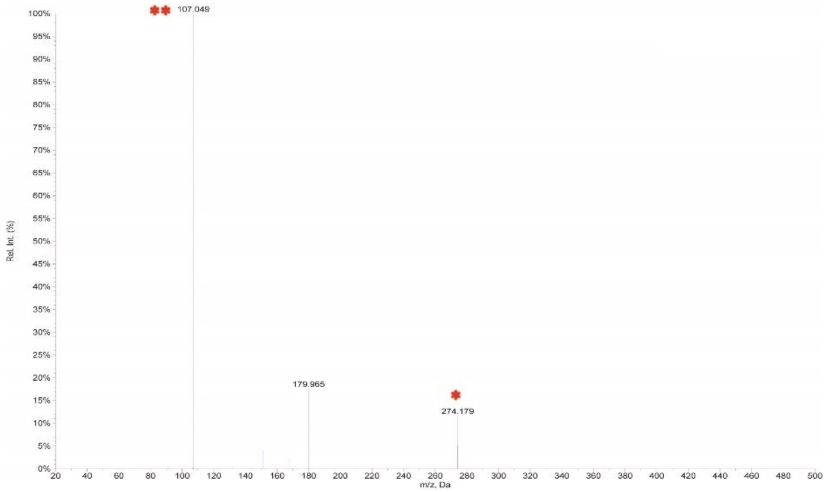

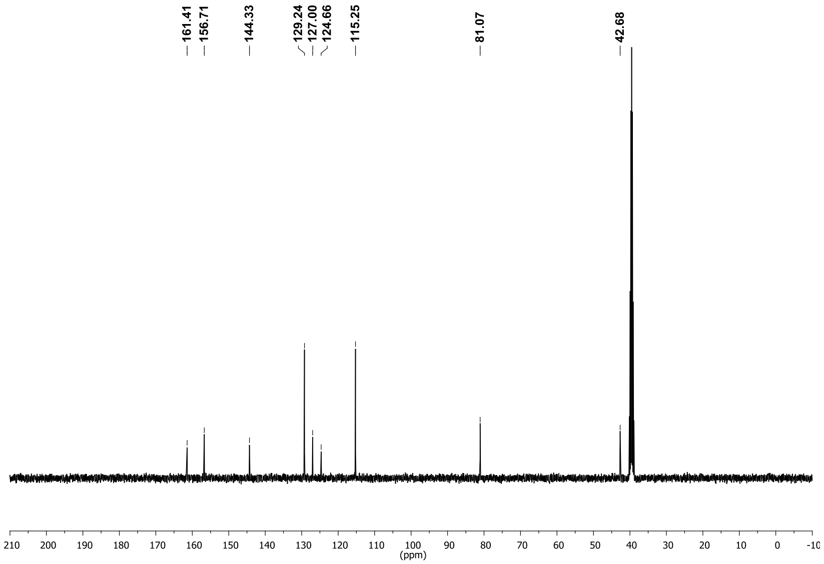

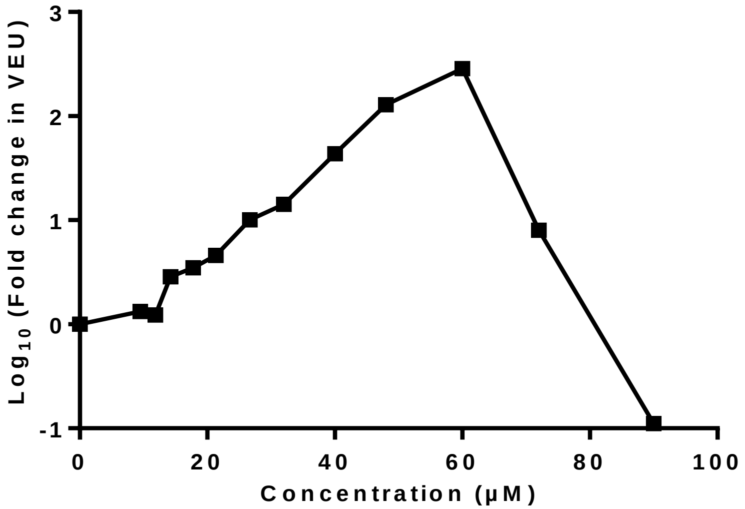

Compound ID: 44

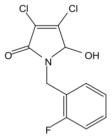

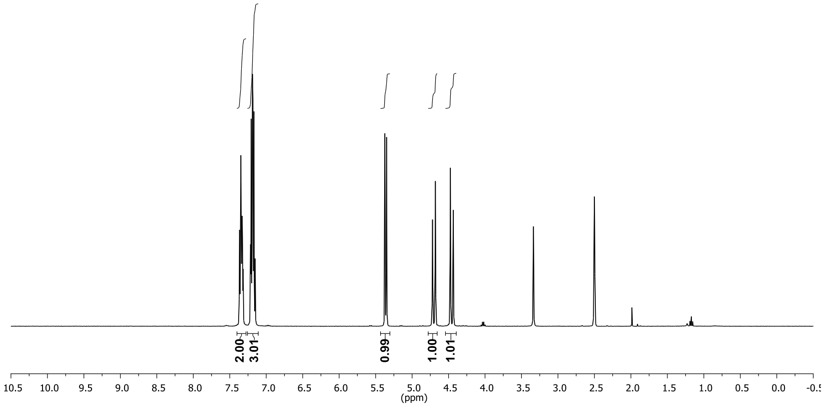

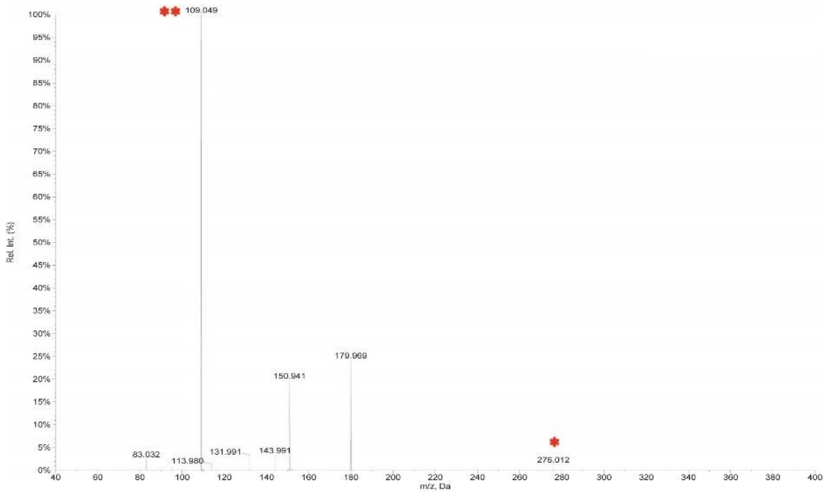

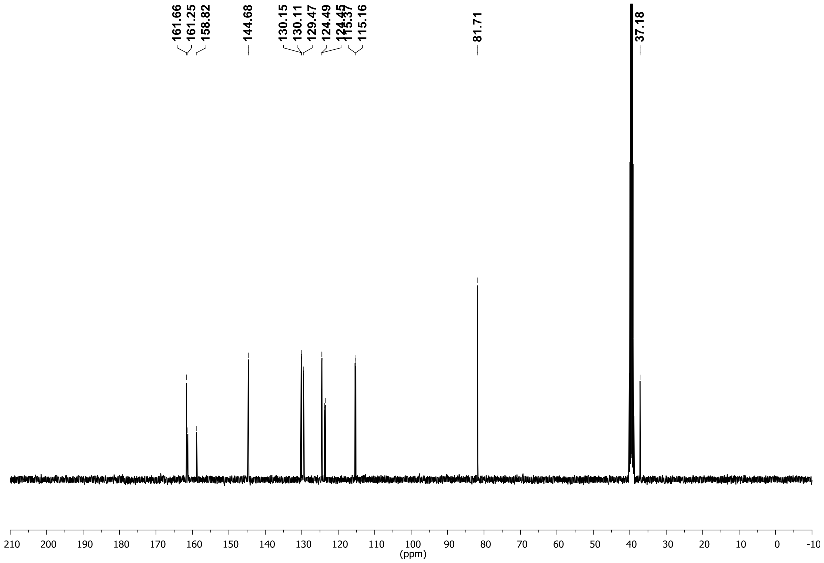

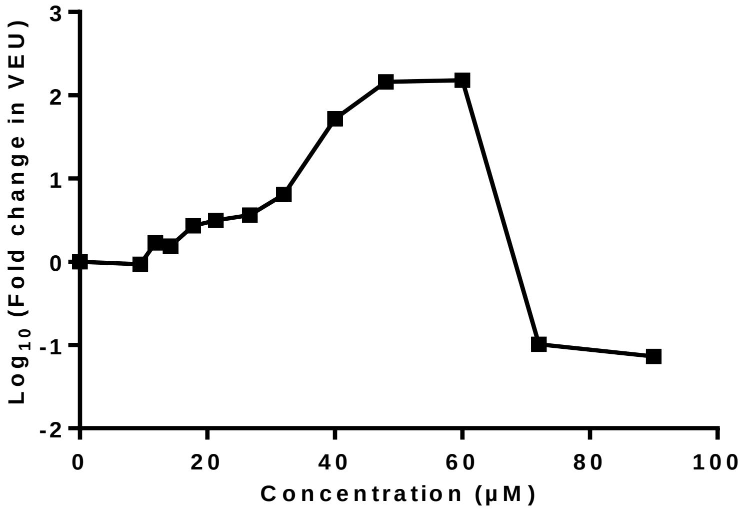

Compound ID: 45

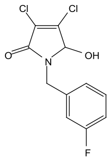

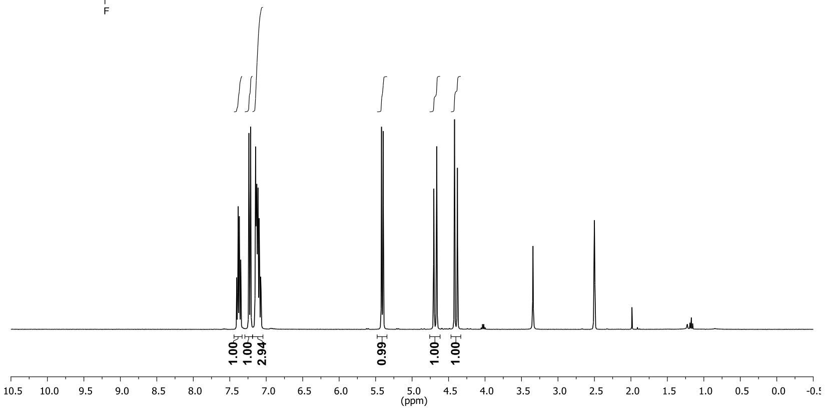

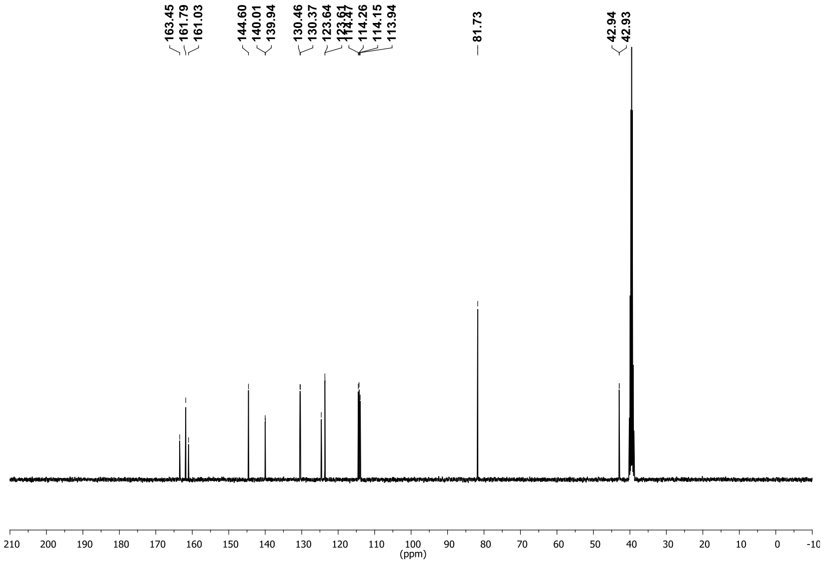

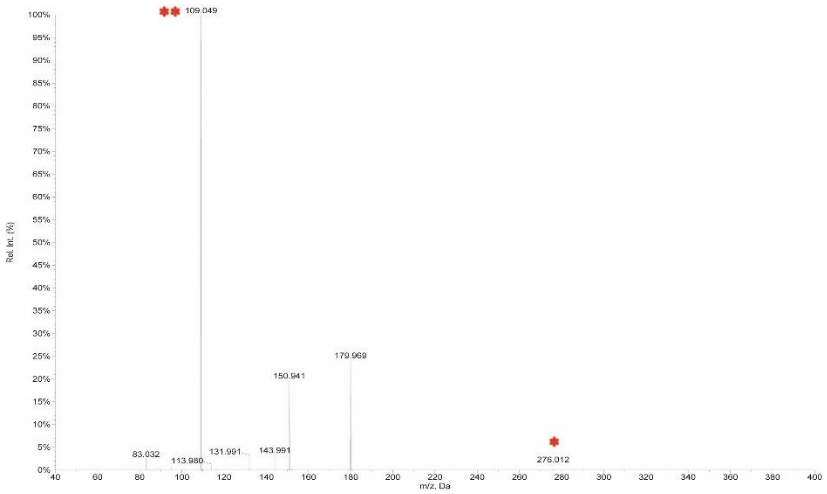

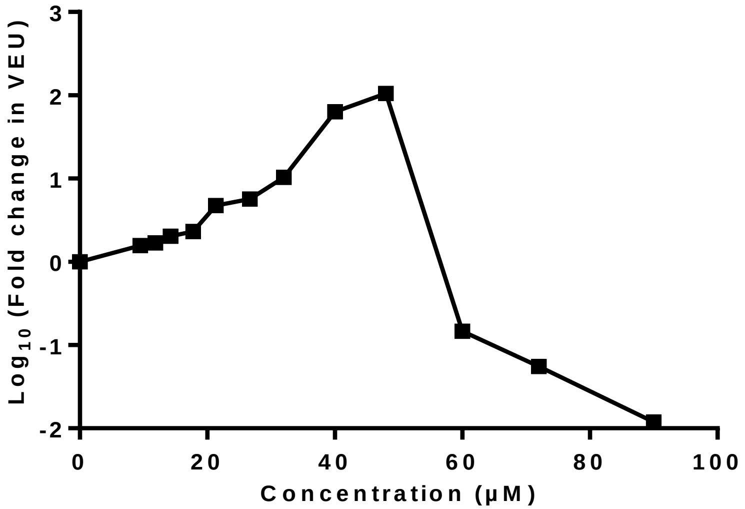

Compound ID: 46

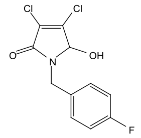

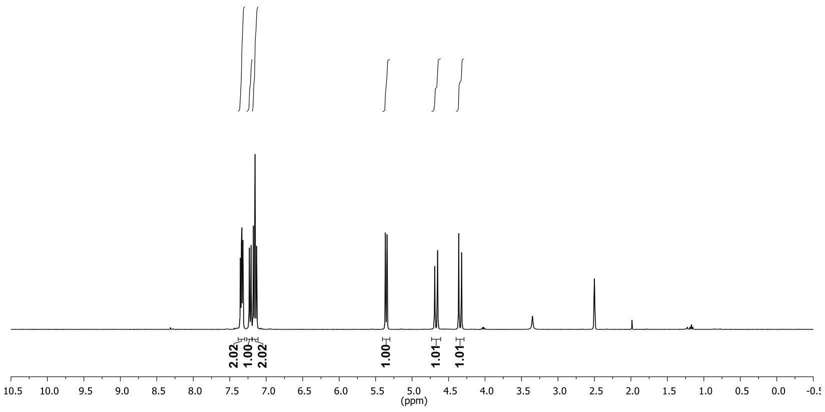

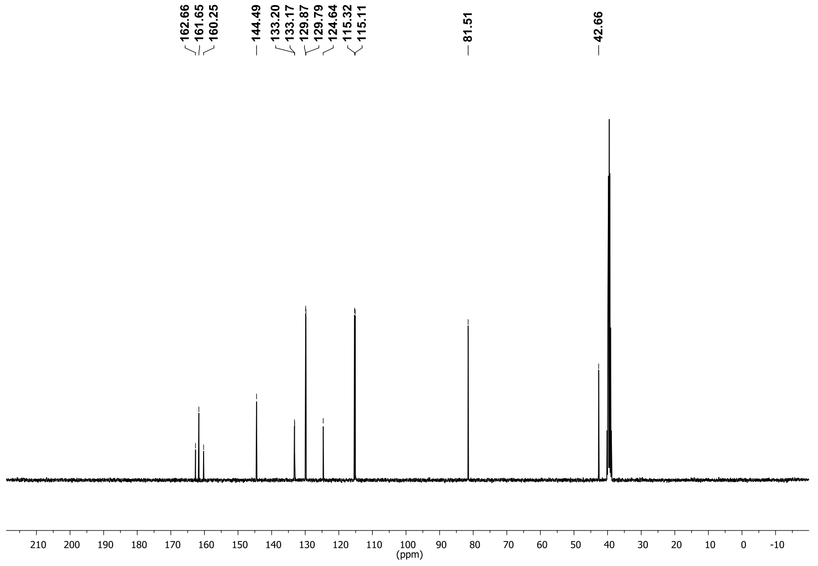

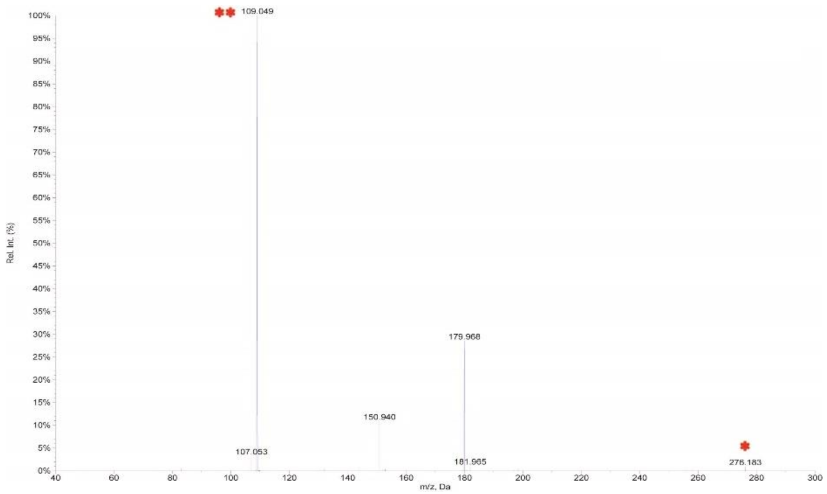

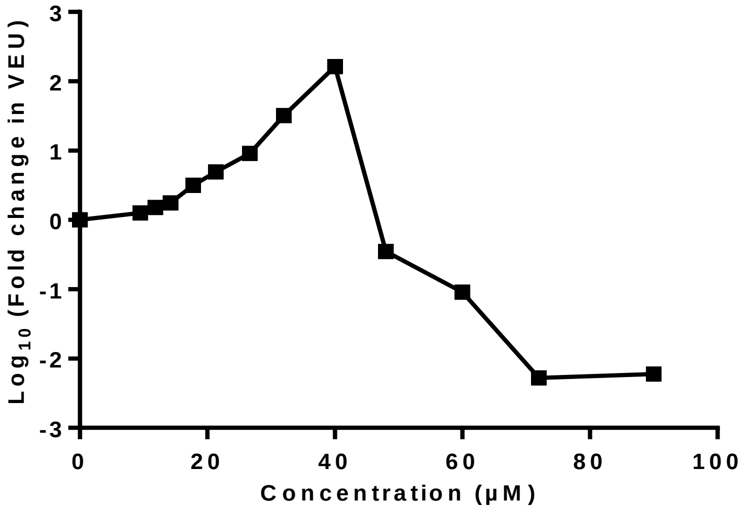

Compound ID: 47

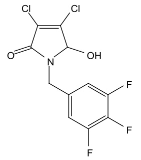

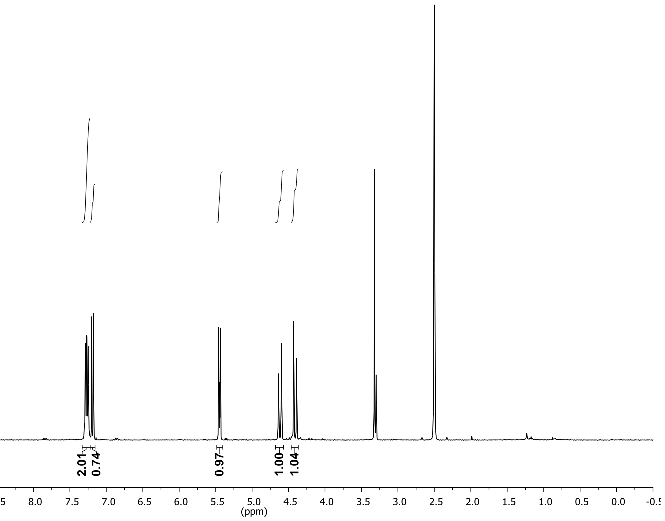

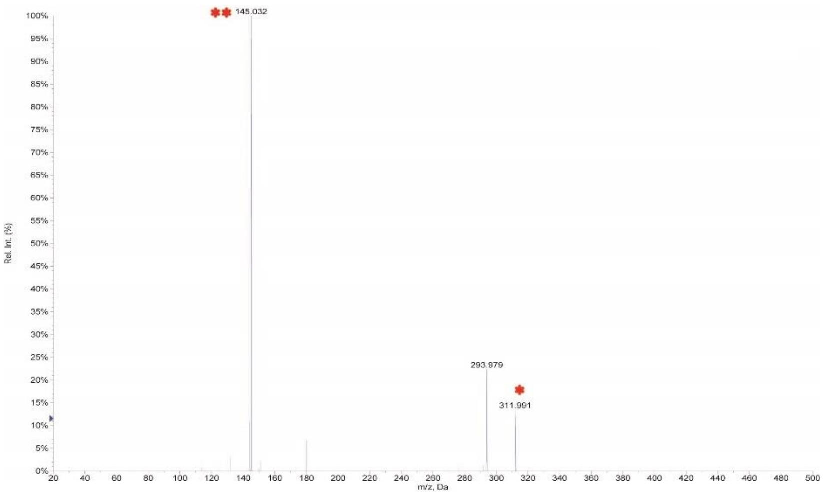

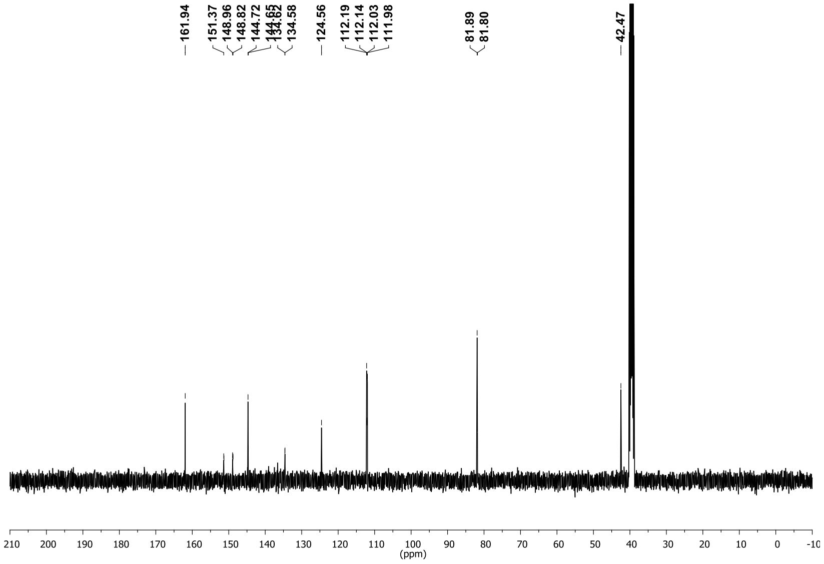

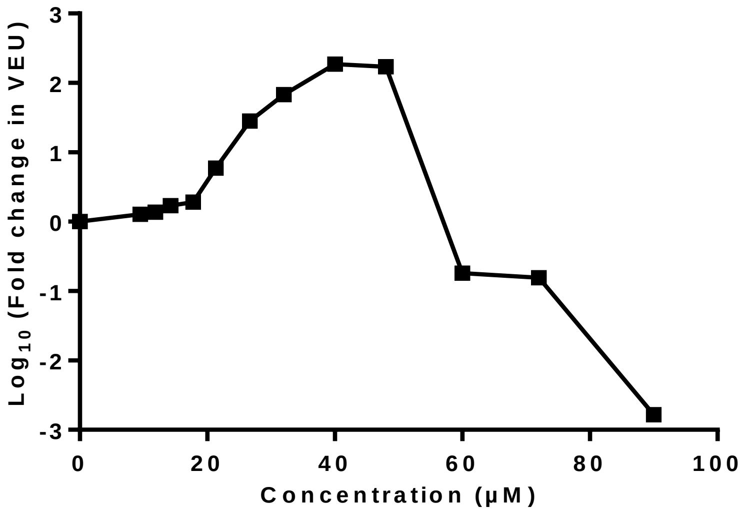

Compound 48

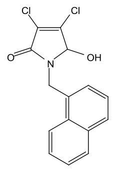
Cl
Cl
O
N
OH

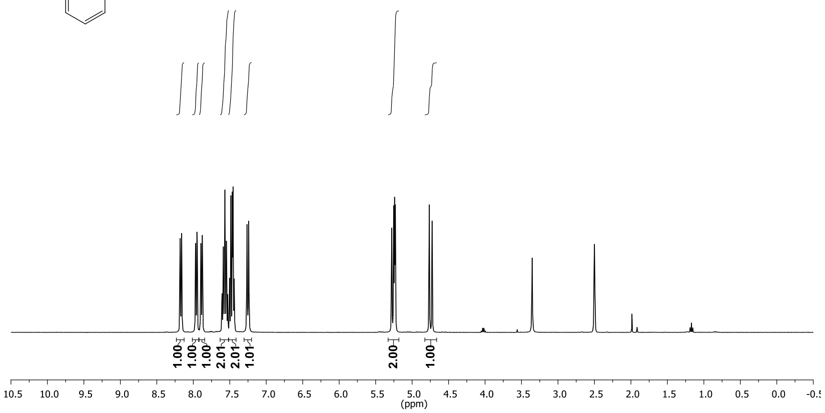
1.00
1.00
1.00
2.01
2.01
1.01
2.00
1.00
10.5 10.0 9.5 9.0 8.5 8.0 7.5 7.0 6.5 6.0 5.5 5.0 4.5 4.0 3.5 3.0 2.5 2.0 1.5 1.0 0.5 0.0
(ppm)

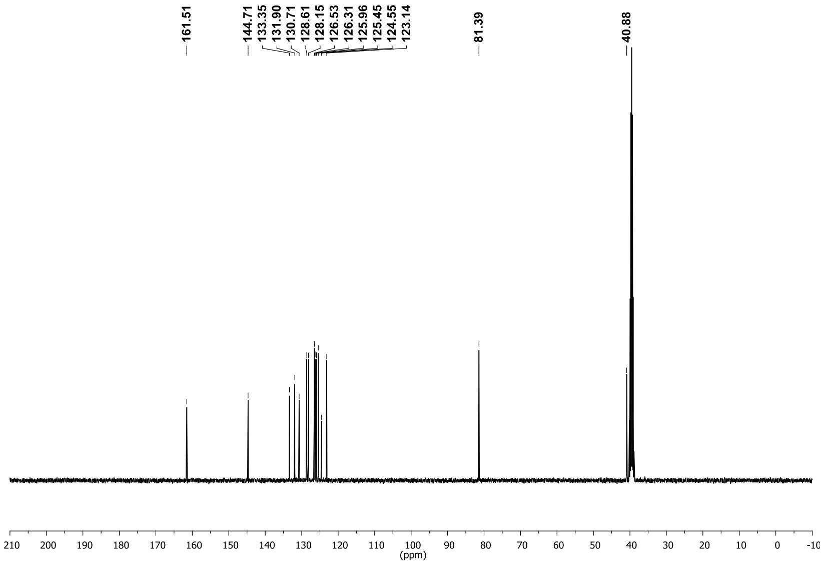
161.51
144.71
133.35
131.90
130.71
128.61
128.15
126.53
126.31
125.96
125.45
124.55
123.14
81.39
40.88
210 200 190 180 170 160 150 140 130 120 110 100 90 80 70 60 50 40 30 20 10 0
(ppm)

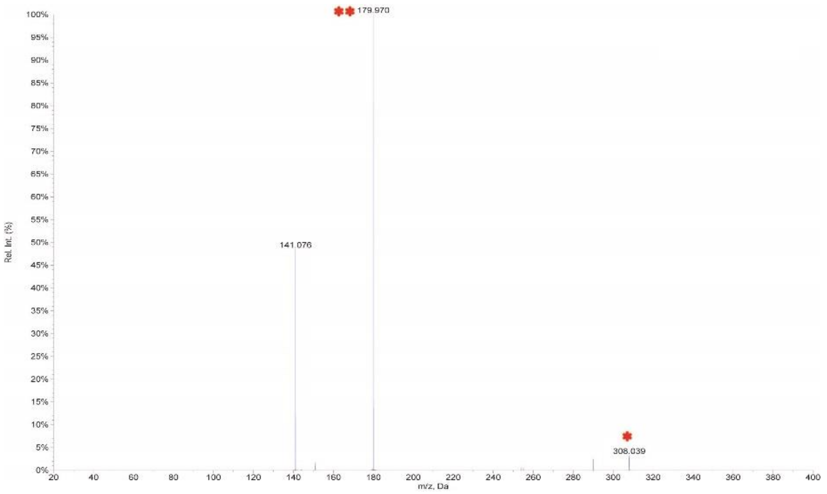
179.970
141.076
308.039
Rel. Int. (%)
m/z, Da

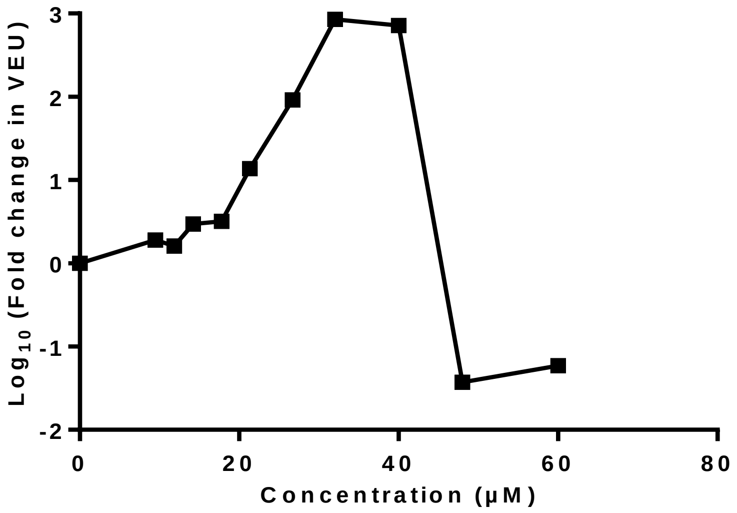
Log10 (Fold change in VEU)
Concentration (µM)
3
2
1
0
-1
-2
0
20
40
60
80

Compound 49

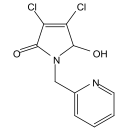
Cl
Cl
O
N
OH
N

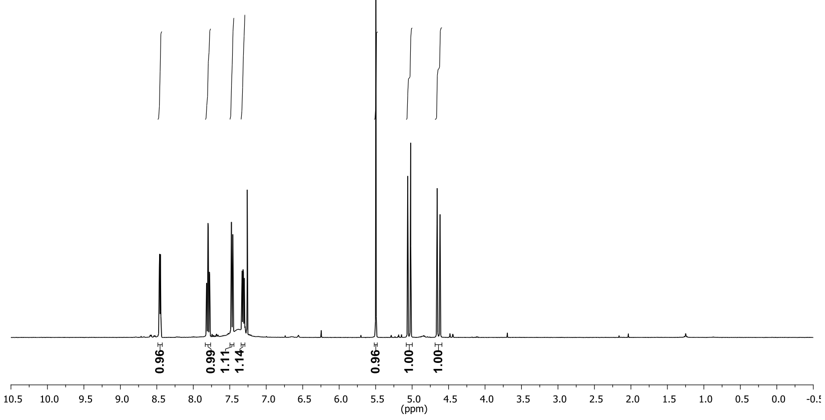
0.96
0.99
1.11
1.14
0.96
1.00
1.00
10.5 10.0 9.5 9.0 8.5 8.0 7.5 7.0 6.5 6.0 5.5 5.0 4.5 4.0 3.5 3.0 2.5 2.0 1.5 1.0 0.5 0.0
(ppm)

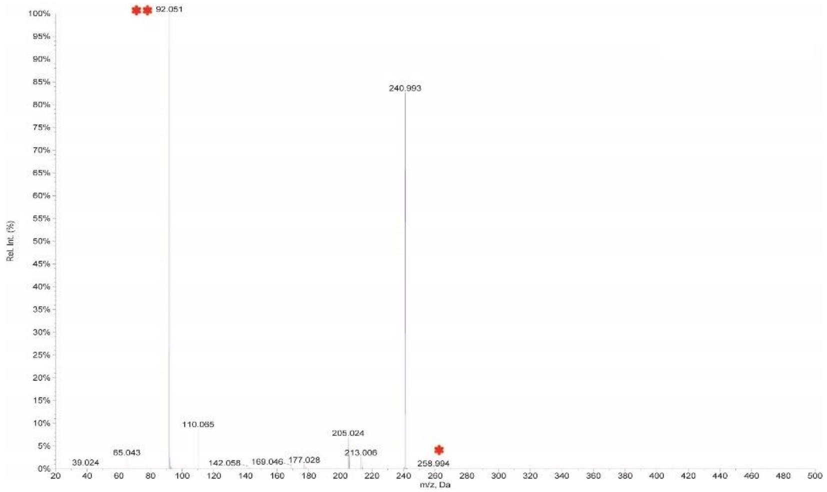
92.051
240.993
110.085
205.024
213.006
65.043
39.024
142.058
169.046
177.028
258.994
Rel. Int. (%)
m/z, Da

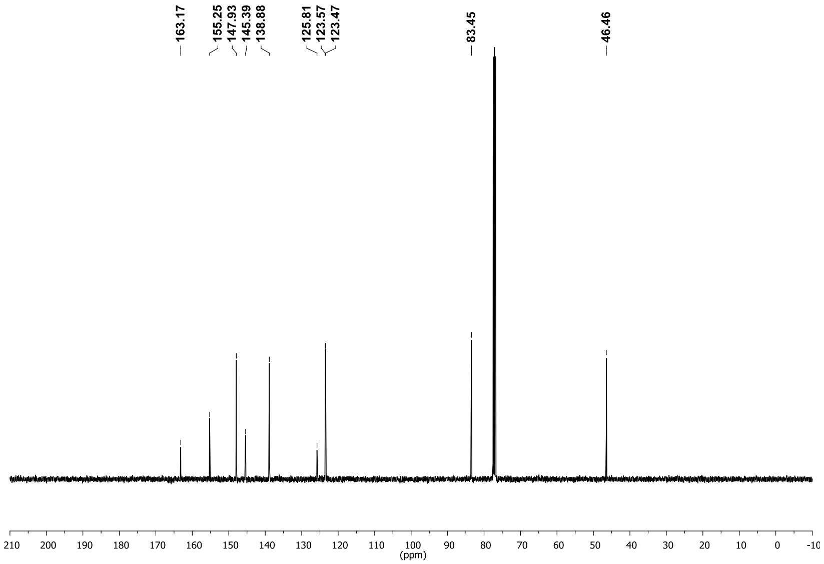
163.17
155.25
147.93
145.39
138.88
125.81
123.57
123.47
83.45
46.46
210 200 190 180 170 160 150 140 130 120 110 100 90 80 70 60 50 40 30 20 10 0
(ppm)

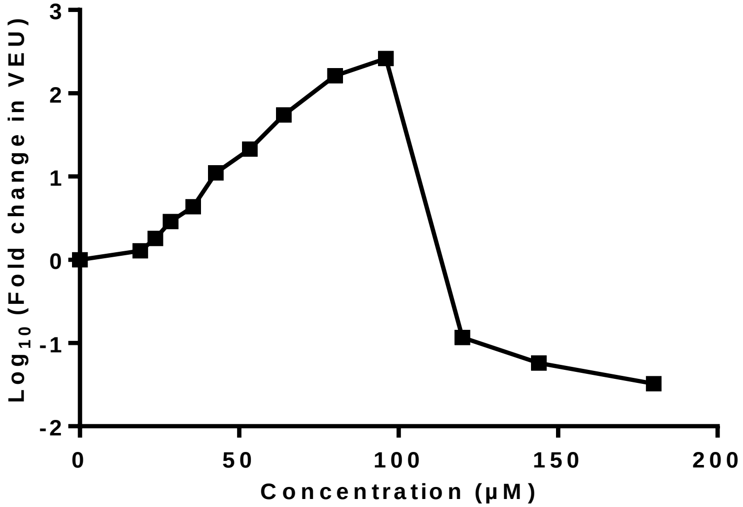
Log10 (Fold change in VEU)
Concentration (μM)
3
2
1
0
-1
-2
0
50
100
150
200

Compound 50

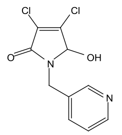
Cl
Cl
O
N
OH
N

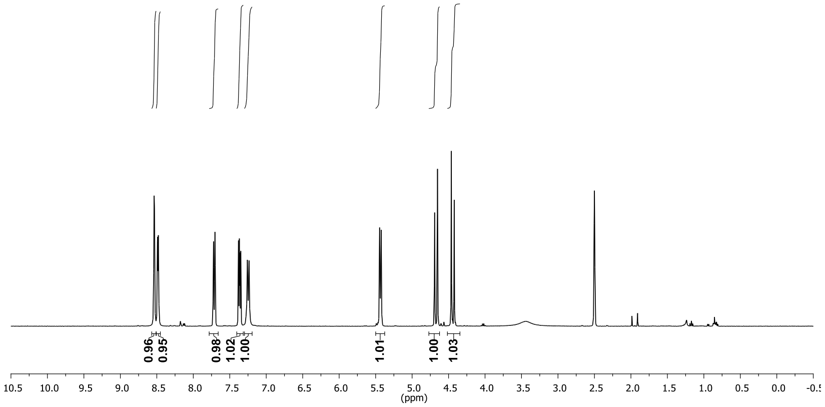
0.96
0.95
0.98
1.02
1.00
1.01
1.00
1.03
10.5 10.0 9.5 9.0 8.5 8.0 7.5 7.0 6.5 6.0 5.5 5.0 4.5 4.0 3.5 3.0 2.5 2.0 1.5 1.0 0.5 0.0
(ppm)

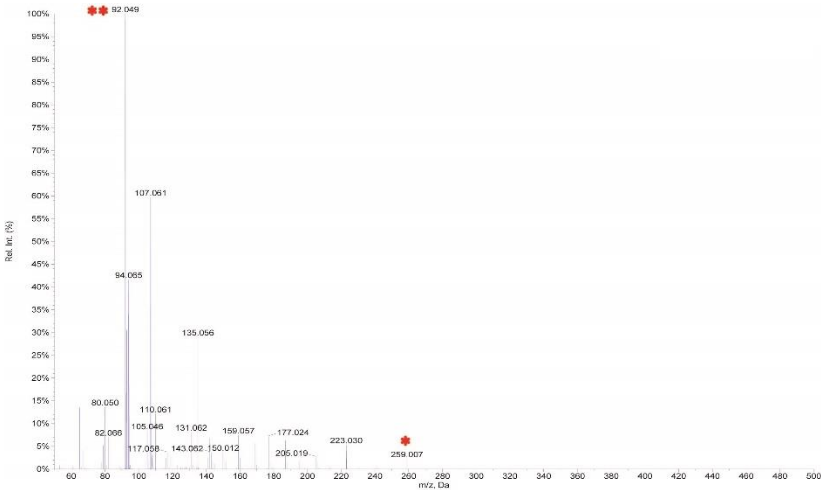
92.049
107.061
94.065
135.056
80.050
110.061
82.066
105.046
131.062
159.057
177.024
223.030
117.058
143.062
150.012
205.019
259.007
Rel. Int. (%)
m/z, Da

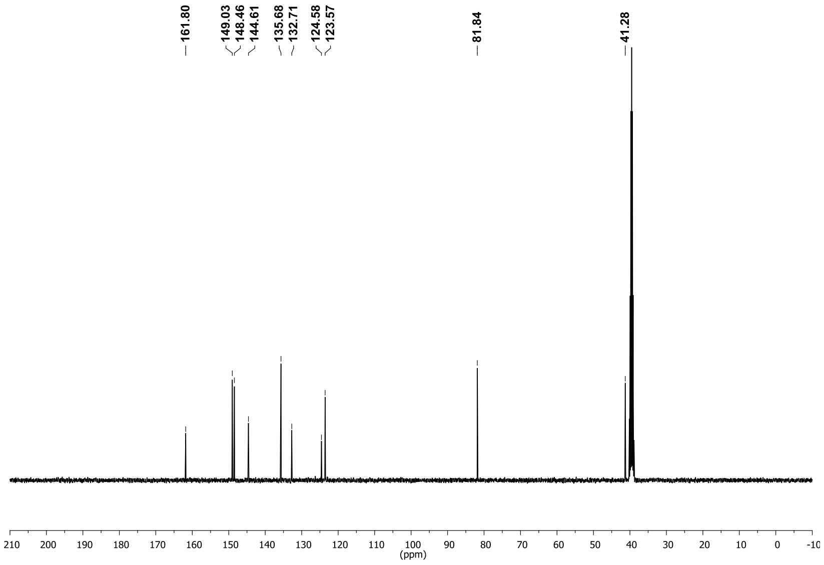
161.80
149.03
148.46
144.61
135.68
132.71
124.58
123.57
81.84
41.28
210 200 190 180 170 160 150 140 130 120 110 100 90 80 70 60 50 40 30 20 10 0
(ppm)

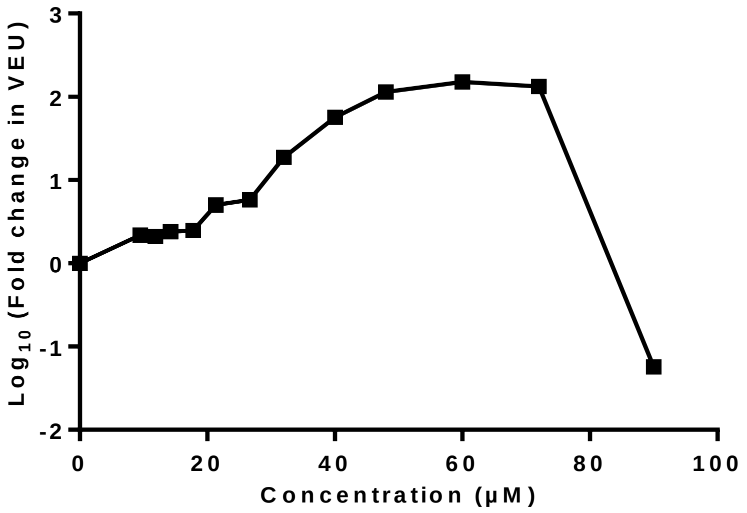
Log10 (Fold change in VEU)
Concentration (μM)
3
2
1
0
-1
-2
0
20
40
60
80
100

Compound 51

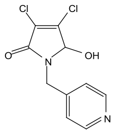
Cl
Cl
O
N
OH
N

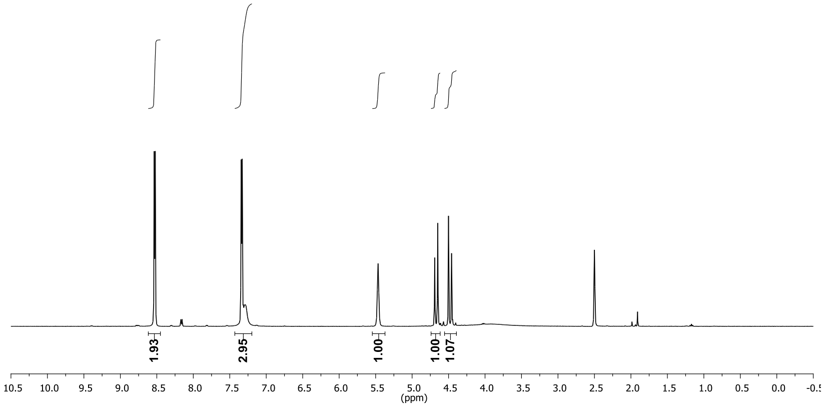
1.93
2.95
1.00
1.00
1.07
10.5 10.0 9.5 9.0 8.5 8.0 7.5 7.0 6.5 6.0 5.5 5.0 4.5 4.0 3.5 3.0 2.5 2.0 1.5 1.0 0.5 0.0
(ppm)

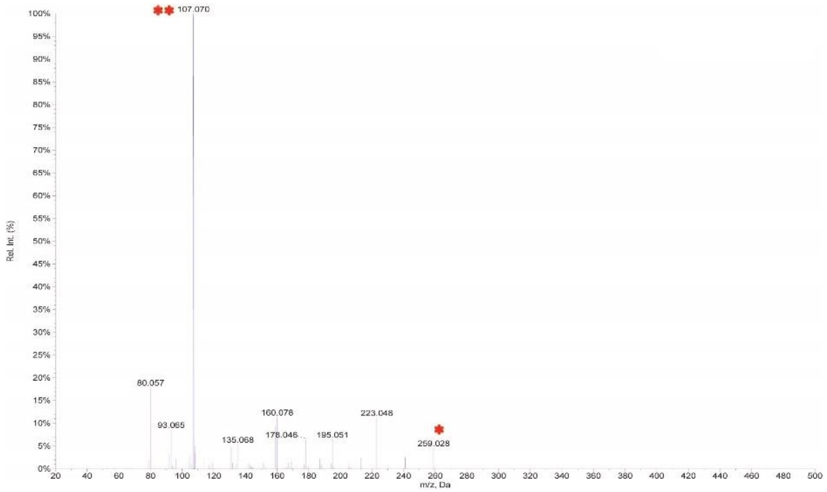
107.070
80.057
93.065
135.068
160.076
178.045
195.051
223.048
259.028
Rel. Int. (%)
m/z, Da

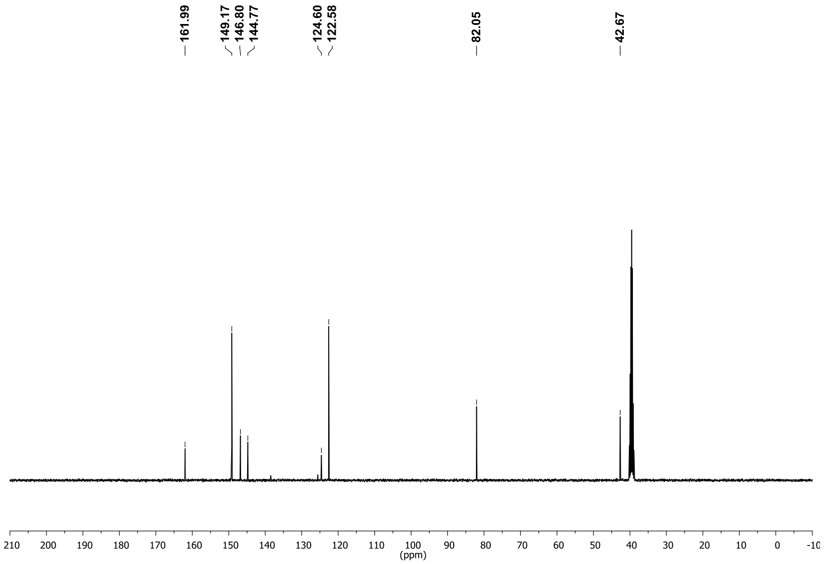
161.99
149.17
146.80
144.77
124.60
122.58
82.05
42.67
210 200 190 180 170 160 150 140 130 120 110 100 90 80 70 60 50 40 30 20 10 0
(ppm)

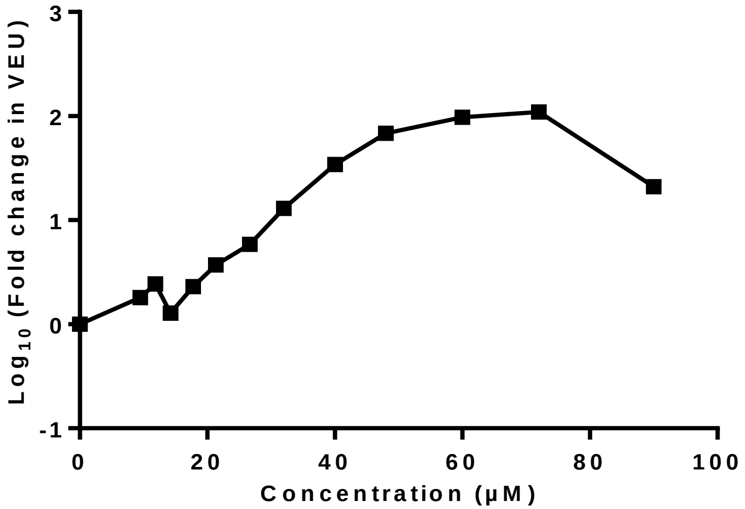
Log_{10} (Fold change in VEU)
Concentration (μM)

MRM Transition Data

Compound ID	Quantitative Transition		Confirmatory Transition		Collision Energy (eV)	LC-MRM Gradient
	Parent Ion (m/z)	Fragment Ion (m/z)	Parent Ion (m/z)	Fragment Ion (m/z)		
1	229.0	163.0	231.0	165.0	25	A
2	318.9	209.0	318.9	237.0	18	A
3	300.1	193.0	302.1	195.0	20	A
4	237.0	209.0	239.0	211.0	15	A
5	152.9	61.0	152.9	117.0	22	A
6	183.0	150.9	185.0	152.9	15	B
7	207.0	150.9	209.0	152.9	20	B
8	229.0	165.0	229.0	158.1	22	A
9	168.0	132.0	170.0	134.0	20	B
10	258.0	91.1	260.0	91.1	20	A
11	242.1	91.1	244.1	91.1	22	A
12	272.0	91.1	274.0	91.1	20	A
13	300.0	91.1	300.0	258.0	20	A
14	256.0	91.1	256.0	178.0	17	B
15	334.1	227.0	336.1	229.0	20	A
16	190.1	91.1	190.1	112.0	18	B
17	218.1	91.1	218.1	140.1	22	B
18	248.0	180.0	250.0	182.0	18	B
19	264.0	180.0	266.0	182.0	17	A
20	240.1	91.1	240.1	133.0	27	A
21	341.1	323.1	343.1	325.1	15	B
22	182.0	146.0	184.0	148.0	25	A
23	ND*					
24	210.0	132.0	212.0	134.0	20	B
25	208.0	150.9	210.0	152.9	23	A
26	208.0	150.9	210.0	152.9	23	A
27	206.0	134.0	208.0	134.0	23	A
28	281.1	178.0	283.0	180.0	25	C
29	272.1	105.1	274.1	105.1	25	A
30	286.1	164.0	286.1	117.1	20	A
31	300.1	131.1	302.1	131.1	23	A
32	ND*					
33	282.0	133.1	284.0	133.1	20	A
34	272.0	105.1	272.0	168.0	25	A
35	334.0	167.1	336.0	167.1	25	A
36	288.0	121.1	290.0	121.1	18	A
37	274.0	107.1	276.0	107.1	30	B
38	292.0	125.0	294.0	125.0	22	A
39	336.0	169.0	338.0	169.0	22	A

40	326.0	159.0	328.0	159.0	25	A
41	272.0	105.1	274.0	105.1	20	A
42	288.0	121.1	290.0	121.1	17	A
43	274.0	107.1	276.0	107.1	20	A
44	276.0	109.1	278.0	109.1	22	A
45	276.0	109.1	276.0	258.0	20	A
46	276.0	109.1	278.0	109.1	22	A
47	312.0	145.0	312.0	294.0	18	B
48	308.0	180.0	310.0	182.0	17	A
49	259.0	241.0	261.0	243.0	20	B
50	259.0	92.1	261.0	92.1	35	A
51	259.0	107.1	261.0	107.1	32	B

* - compound not analyzed

LC-MRM Gradient A:

Retention Time (min)	Flow Rate (μL/min)	Water (0.1% formic acid) (%)	Acetonitrile (0.1% formic acid) (%)
0	250	98	2
5	250	50	50
5.5	275	20	80
10	350	0	100

LC-MRM Gradient B:

Retention Time (min)	Flow Rate (μL/min)	Water (0.1% formic acid) (%)	Acetonitrile (0.1% formic acid) (%)
0	250	98	2
2	250	50	50
5.5	275	20	80
10	350	0	100

LC-MRM Gradient C

Retention Time (min)	Flow Rate (μL/min)	Water (0.1% formic acid) (%)	Acetonitrile (0.1% formic acid) (%)
0	320	100	0
4.1	325	75	25
7.8	375	50	50
9.7	425	25	75
11.6	450	0	100

References

1. Bellina, F., Anselmi, C., Martina, F. & Rossi, R. Mucochloric acid: a useful synthon for the selective synthesis of 4-aryl-3-chloro-2(5H)-furanones, (Z)-4-aryl-5-[1-(aryl)methylidene]-3-chloro-2(5H)-furanones and 3,4-diaryl-2(5H)-furanones. *Eur. J. Org. Chem.* **2003,** 2290–2302 (2003).

2. Ratts, K. W. & Phillips, W. G. Trimethyl Phosphite Displacement on Mucochloryl Chloride. *J. Org. Chem.* **39,** 3300–3301 (1974).

3. Zhang, J., Blazecka, P. G. & Davidson, J. G. First direct reductive amination of mucochloric acid: A simple and efficient method for preparing highly functionalized α,β-unsaturated γ-butyrolactams. *Org. Lett.* **5,** 553–556 (2003).

4. Holz, J. *et al.* A highly tunable family of chiral bisphospholanes for Rh-catalyzed enantioselective hydrogenation reactions. *Chem. - A Eur. J.* **12,** 5001–5013 (2006).

5. Yamashita, S., Mase, N. & Takabe, K. Chemoenzymatic total synthesis and determination of the absolute configuration of (S)-nebracetam. *Tetrahedron Asymmetry* **19,** 2115–2118 (2008).
